# Tunable Linkers for Dynamic Thiol-Based Bioconjugation Strategies

**DOI:** 10.1021/acs.biomac.5c02149

**Published:** 2026-01-08

**Authors:** G. Cianfoni, L. Pisano, D. M. Varouhaki, G. Centioni, A. Calcaterra, F. Ghirga, C. M. Athanassopoulos, B. Botta, S. Cammarone, P. Baiocco, D. Quaglio

**Affiliations:** † Department of Chemistry and Technology of Drugs, 9311 Sapienza University of Rome, P.le Aldo Moro 5, 00185 Rome, Italy; ‡ Department of Chemistry, 37795 University of Patras, GR-26504 Rio-Patras, Greece; § Department of Biochemical Sciences “Alessandro Rossi Fanelli”, Sapienza University of Rome, P.le A. Moro 5, 00185 Rome, Italy

## Abstract

Recent advancements in bioconjugation chemistry have increasingly focused on thiol-based strategies, offering reversible and stimuli-responsive mechanisms, particularly suited for biomedical applications. This review aims to provide a critical overview of the latest developments in thiol-containing linkers, such as disulfide bonds and photocleavable groups, emphasizing their role in enabling controllable and often reversible conjugation of biomolecules. The review will explore several applications, including peptide synthesis and peptide-stapling strategies, antibody–drug conjugates (ADCs), and responsive biomaterials, categorize key classes of cleavable thiol-based linkers, and analyze their mechanisms. Covering the literature from the past 15 years, focusing on innovations until 2024, this review addresses the chemical foundations and practical implementations of these systems, identifying current limitations and proposing future directions for designing selective, biocompatible, and functionally dynamic conjugation platforms.

## Graphical abstract

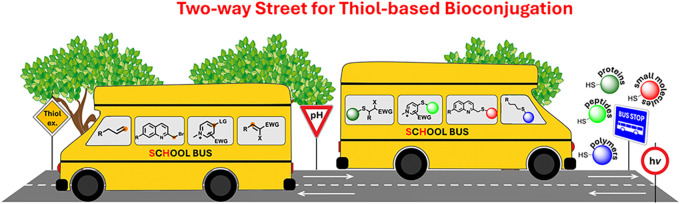

## 1 Introduction

The field of bioconjugation chemistry has made significant progress, enhancing the methodologies available for labeling, profiling, mapping, and enriching biomolecules in both *in vitro* and *in vivo* contexts.^1–8^ Recently, there has been increasing interest in conjugation strategies that allow for tunable downstream cleavage, facilitating the regeneration of unmodified biomolecules and the manipulation of their structures, functions, and dynamics.^9–14^ A variety of chemically cleavable or reversible bioconjugation techniques have been developed for site-specific chemical modification of biomolecules, *i.e.*, lysine, tryptophan, methionine, and tyrosine.^15^ Among them, cysteine is perhaps one of the most attractive and convenient targets due to its high nucleophilicity, relatively low natural abundance, and the ease of its introduction into a specific site by site-directed mutagenesis.^16–20^ Numerous papers in the literature document the development and characterization of reversible cysteine-reactive reagents, highlighting the remarkable structural diversity of the chemical linkers used.^10,21,22^ Reversible thiol conjugation leverages a wide array of electrophilic scaffolds and cleavage mechanisms, including: Michael acceptors (*e.g.*, maleimides, enones, ynones, and acrylates); 1,2-Addition systems, featuring electrophiles—like cyanopyridines and iminoboronates; aliphatic electrophiles, including benzylic and α-halo scaffolds, undergoing S_N_2 reactions; aromatic S_N_Ar-based linkers, using electron-deficient aromatic systems; disulfide linkers, which undergo thiol–disulfide exchange under reductive intracellular conditions; thiol–ene reactions, traditionally considered irreversible, now engineered for dynamic behavior.^4,21,23,24^ One notable application of tunable linkers for thiols is in the dynamic, reversible site-specific labeling of proteins by using photoswitchable systems.^25^ The use of cleavable linkers as regioselective thiol-protecting groups in the chemical synthesis of peptides was reported.^26^ Cleavable peptide-stapling techniques using thiols were explored, showing promise for the development of peptide modulators that aim to probe traditionally undruggable protein–protein interactions (PPIs) and investigate their dynamics.^27–29^ Furthermore, the exploitation of reversible cysteine modification within antibody-drug conjugates (ADCs) has demonstrated significant potential in advancing the clinical treatment of cancer.^30^ Beyond these applications, cleavable bioconjugation chemistry has been harnessed for probing enzymatic activities, creating protein ligands, and profiling proteomes.^31,32^ This review, covering the literature up to 2024, aims to provide a comprehensive and critical overview of recent advances in thiol-based bioconjugation strategies with a particular emphasis on reversible and stimuli-responsive systems. A central objective is to explore how thiol-selective linkers have been engineered to allow for precise, controllable, and often reversible conjugation of biomolecules, addressing the current needs in biomedical applications. Our exploration reveals that thoughtful design principles have a significant influence on reaction pathways, affect yields, and enhance the overall efficacy of bioconjugate formation. For each subgroup examined, we discuss the mechanisms involved in the deconjugation processes when relevant. This includes a thorough investigation of the specific conditions that can promote these reactions in the dynamic field of bioconjugation chemistry. Moreover, the implications for real-world applications are briefly explored. In the following Sections, thiol moieties from conjugated biomacromolecules (*e.g.*, peptides, proteins, antibodies, and nanobodies) are represented in the schemes with orange spheres to simplify and focus the reader’s attention on the reactive site of the dynamic linkers.

## 2 Thiol-Michael Addition Reaction

Bioconjugation reactions are fast chemical transformations that ideally work under mild conditions with high yield and selectivity. In 2001, and more recently with the award of the Nobel Prize in Chemistry 2022, Barry Sharpless shed light on *click* reactions, *i.e.*, chemical processes with the above-mentioned characteristics.^33^ Besides the most commonly known click reaction (Copper-catalyzed Azide–Alkyne Cycloaddition, CuAAC), Michael addition, and more specifically the thiol-Michael variant, plays a key role in the bioconjugation process of biomacromolecules. It was initially described as an addition of an enolate of an aldehyde or ketone (nucleophile, Michael donor) to the β-carbon of an α,β-unsaturated carbonyl compound (electrophile, Michael acceptor).^34^ Nowadays, the definitions of “donor” and “acceptor” are broadened, and they include other nucleophiles such as thiols, alcohols, amines, enamines, and Gilman reagents, as well as different electrophiles—like unsaturated nitriles, esters, amides, and nitroalkenes. An important feature on the electrophilic side is the presence of an electron-withdrawing group (EWG) conjugated to a double or triple bond, making it more electrophilic.

The reaction proceeds through a simple mechanism (Scheme 1A): (i) the nucleophile reacts with the electron-poor double or triple bond at the β-position leading to the formation of a carbanion in the α-position stabilized by the EWG; (ii) the carbanion intermediate can then react either with a second electrophile for further functionalization or with a protic species to give its protonated form.

**Scheme 1:**
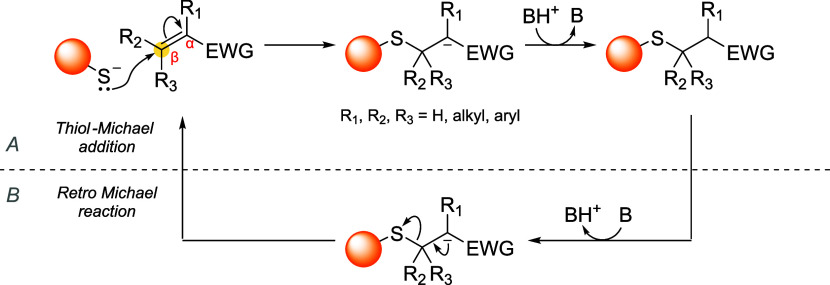
Reaction Mechanism of (A) Thiol-Michael Addition and (B) Retro-Michael Reaction^a^

a Bioconjugation site is marked with a yellow dot.

This reaction has been comprehensively studied over the last century, and a lot of efforts have been made to elucidate the insights of the thiol-Michael reaction in bioconjugation chemistry. This variant has proven to be an efficient method for the selective bioconjugation of cysteine residues in biomacromolecules. This effectiveness is attributed not only to the advantages of click reactions but also to the low abundance of cysteine and its nucleophilicity at physiological pH (p*K*_a_ ∼ 8).^4,35^ This unique property renders it the only naturally occurring amino acid with a p*K*_a_ value close to the physiological pH. Moreover, according to “Hard and Soft Acid Base” (HSAB) theory, thiols are soft nucleophiles and react faster with Michael acceptors, unlike amines and alcohols (*e.g.*, in lysine and serine). Thiol-Michael addition can be easily tuned by changing different parameters such as temperature and pH. It is well-known that the thiol-Michael reaction is slightly affected by temperature,^36,37^ producing stable thiol adducts that do not undergo thiol exchange at room temperature (except for molecules strongly prone to elimination reaction). A relevant demonstration of the dynamic nature of these adducts through different temperatures was reported by Zhang et al. in 2016, who showed that thioether linkages formed via thiol-Michael addition to acrylates can behave as thermally activated dynamic covalent bonds. Specifically, in their study, they reported polymeric networks cross-linked through thioether linkages, which displayed malleable properties upon thermal stimulus at 90 °C, at the same time maintaining mechanical stability and resistance at room temperature. From a mechanistic perspective, the reversibility was attributed to a temperature-induced shift in the equilibrium between the thioether adduct and its constituent thiol and Michael acceptor, without the need of any chemical triggers (Scheme 2).

**Scheme 2:**
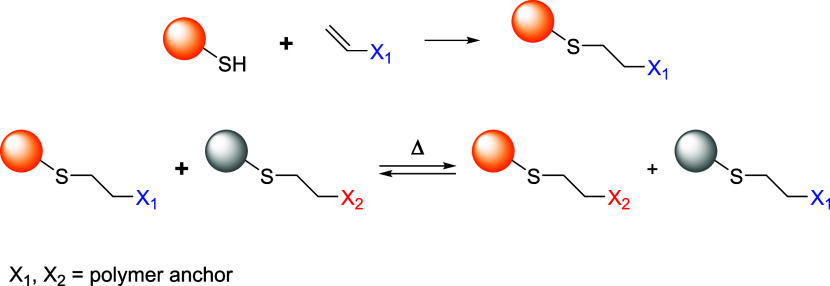
Thermally Induced Dynamic Equilibrium between Two Different Thiol-Adducts

On the other hand, pH is crucial for this reaction and affects both Michael and retro-Michael processes (Scheme 1A,B), which will be detailed later in this section. Schmidt et al. performed a kinetic study to elucidate the addition of glutathione (GSH) to α,β-unsaturated carbonyl systems under physiological conditions, highlighting an exponential increase in the reaction rate with increasing pH.^38^ As expected, the reaction is faster at physiological or higher pH values due to the increased thiolate to thiol ratio, which is negligible at lower pH. The authors pointed out a conversion of GSH in the conjugated form of around 70% in less than 10 min at pH 8, in contrast with a 10% yield at pH 5. Another interesting feature of thiol-Michael addition is its reversibility in basic media, given by the acidity of protons in the α-position of the resulting conjugated product.^39^

In the following sections, we explore the deconjugation reactions of thioether products that could occur after thiol-Michael addition reactions. Our discussion will be focused on the various types of linkers employed in these reactions (Figure 1), examining how the choice of one specific linker influences the reaction pathways, yields, and the overall effectiveness of the thioether formation. We will also analyze the mechanistic aspects of the deconjugation processes, including the conditions that facilitate these reactions and the implications for practical applications in bioconjugation chemistry.

**Figure 1:**
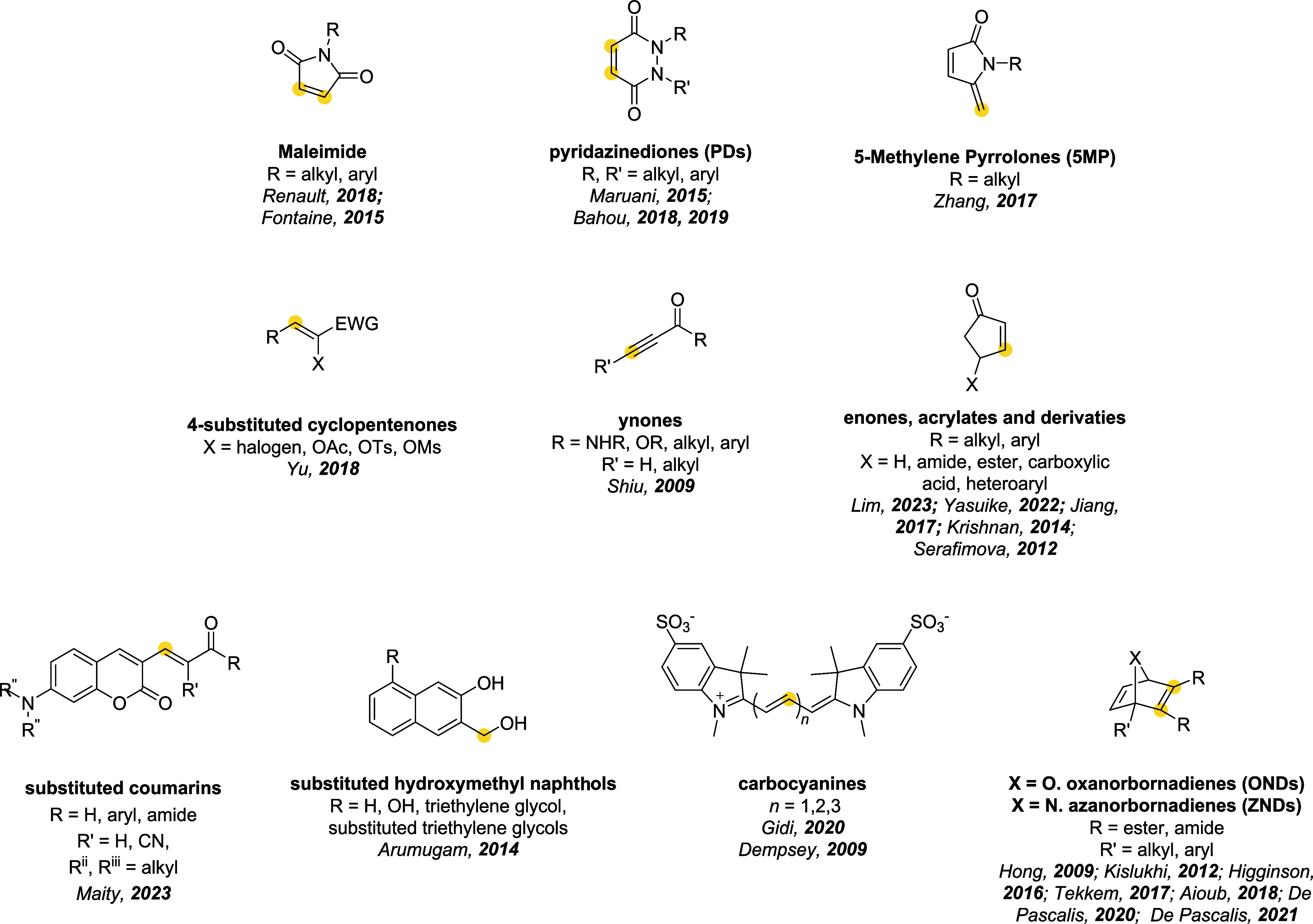
Structures of the thiol-Michael-based linkers. Bioconjugation sites are marked with a yellow dot.

### 2.1 Maleimide-Based Linkers

Maleimides are considered to be the *workhorse* of cysteine bioconjugation, affording excellent functionalization in terms of reaction rate, selectivity, and conversion yield. Moreover, functionalized maleimides are nowadays commercially available or synthetically accessible through efficient, short, and low-cost pathways, making them ubiquitous linkers in biological applications that require the presence of specific functionalities, *i.e.*, fluorophores (benzophenone, coumarin, fluorescein, pyrene, etc.), photoactive functional groups, bio-orthogonal functional groups (alkynes), hydrophilic moieties (*e.g.*, PEG), and pharmacological inhibitors.^40^ In particular, maleimides have been widely exploited for the fluorescence labeling of proteins, PEGylation of peptides, and production of antibody-drug conjugates (ADCs).

Among the huge variety of functionalities present in the wide categories of biomolecules, maleimides have proven to be highly selective toward thiols at nearly physiological pH values. An interesting feature of these linkers is their ability to undergo retro-Michael and thiol-exchange reactions in the presence of blood thiols, such as GSH and human serum albumin (HSA), under physiological conditions (Scheme 3). Fine-tuning of such processes allows access to interesting applications, *i.e.*, the controlled release of a specific conjugated drug. However, the degradation of the bioconjugate entity may be seen as a drawback, even though, on the other hand, the construction of a highly stable nondynamic bioconjugate could shut the doors to releasing conjugated bioactive molecules. Thus, many efforts were made in the opposite direction to develop strategies to increase the stability of maleimide-based conjugates. The degradation of these conjugates via retro-Michael and thiol-exchange reactions was demonstrated by Baldwin and Kiick, who analyzed the process through HPLC and NMR spectroscopy.^41^

**Scheme 3:**
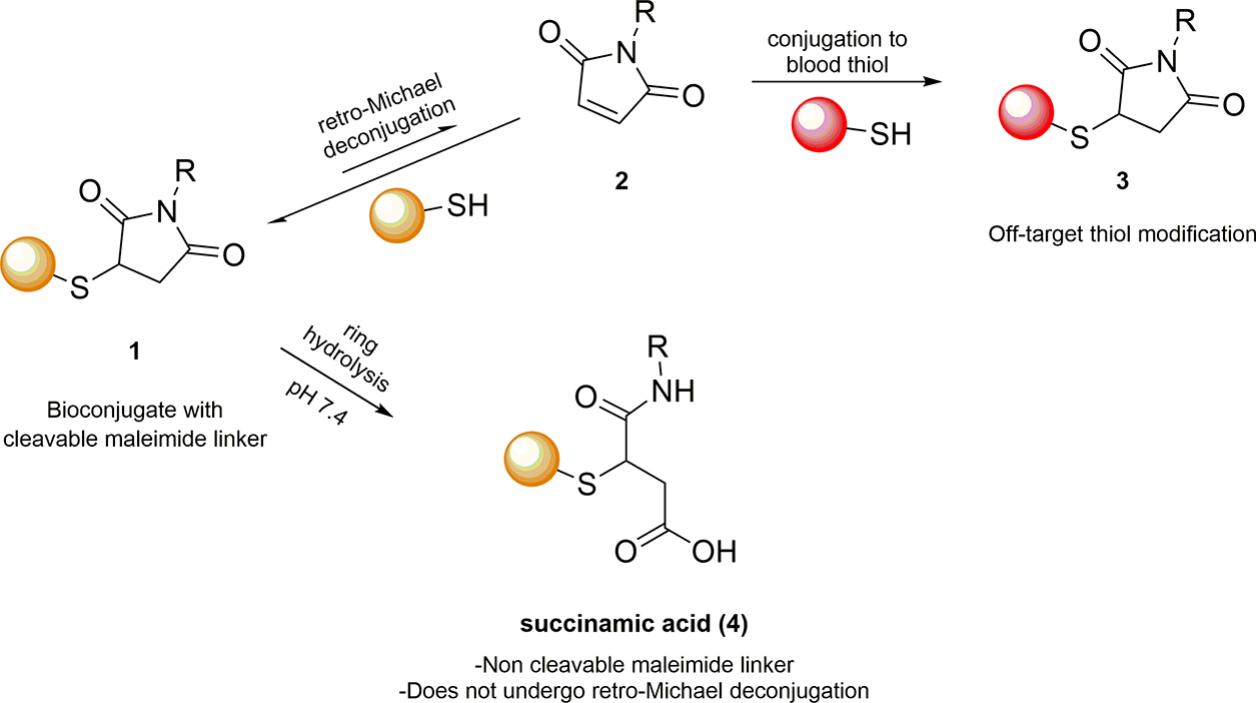
Retro-Michael and Hydrolytic Pathways of Thiol-Maleimide Bioconjugates

In this study, the authors carried out the conjugation of *N*-ethylmaleimide with 4-mercaptophenylacetic acid (thiol p*K*_a_ 6.6), *N*-acetylcysteine (thiol p*K*_a_ 9.5), and 3-mercaptopropionic acid (thiol p*K*_a_ 10.3) in the presence of GSH, highlighting a correlation between the acidity of the thiol and the rate of exchange with GSH. In fact, the rate of the reaction increases as the p*K*_a_ value of the thiol decreases, following the Brønsted relationship.^42^

Another reaction occurring on thiol-maleimide bioconjugates under physiological conditions is the hydrolysis of the maleimide moiety, leading to succinamic acid derivatives that are not prone to thiol-exchange reactions (Scheme 3).^43^ Several parameters were taken into account to study the stability of maleimide-based bioconjugates, such as pH,^44^ temperature, the employment of catalysts,^45^ and the conjugation site environment^46^ in the case of cysteine bioconjugation. However, the most effective strategy turned out to be the functionalization of the maleimide nitrogen. Many functional groups have been explored considering different stereoelectronic and other effects, such as inductive effects, resonance effects, intramolecular catalysis, and hydrophilic effects, summarized in Figure 2.

**Figure 2:**
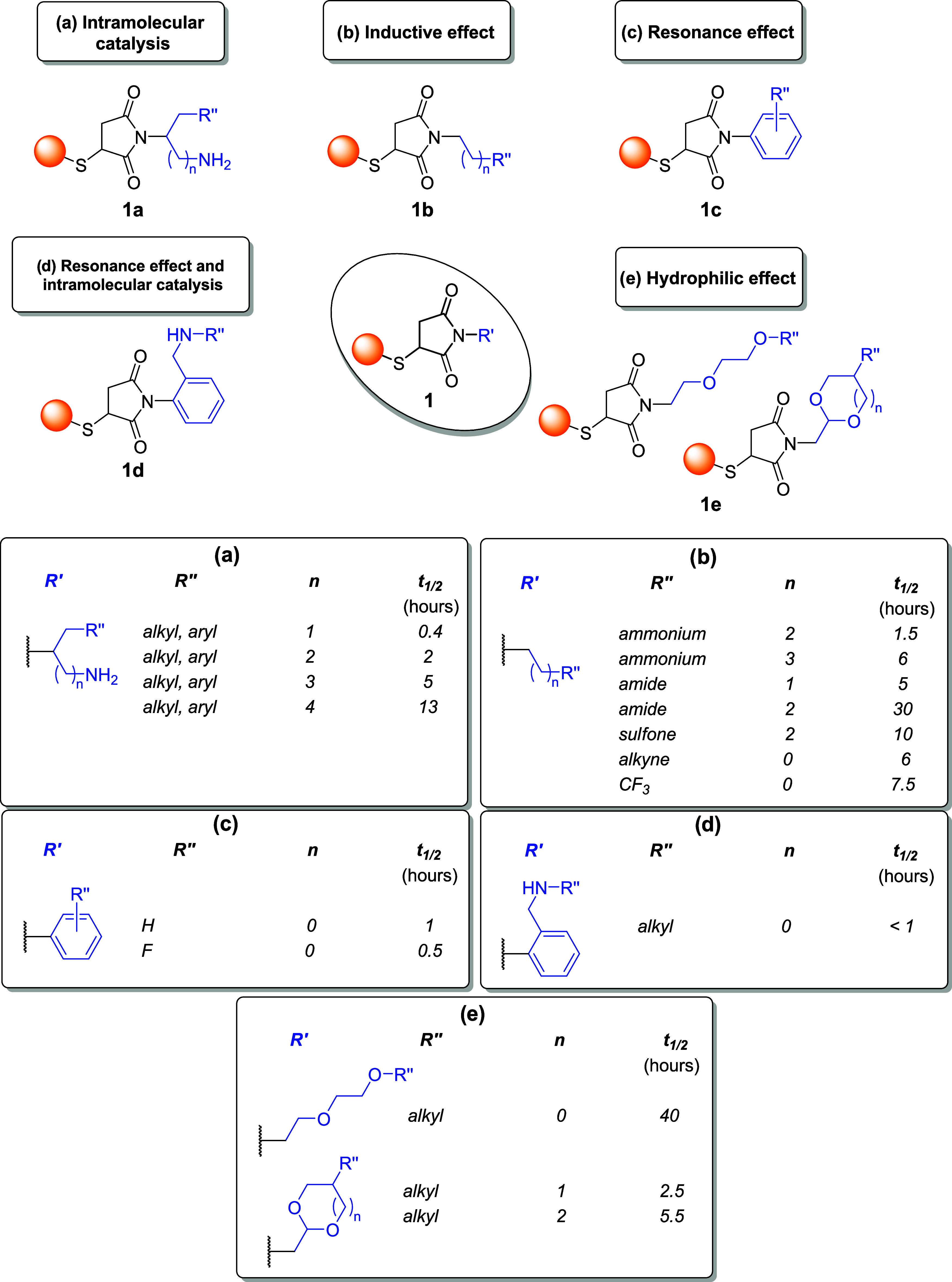
Effects influencing the hydrolysis reaction rate of thiol-maleimide bioconjugates.

Lyon et al. investigated the effect of terminal primary amines (**1a**), noticing a dramatic increase in the rate of hydrolysis of the maleimide moiety compared to *N*-ethylmaleimide due to intramolecular amine-based catalysis.^47^ Such an effect was further explored by Fontaine et al., who attributed the acceleration of the ring-opening hydrolysis rates to inductive effects rather than intramolecular catalysis. This statement was proved by introducing quaternary amines on the *N*-alkyl group of maleimides (**1b**), which lack any basic properties but increase the rate of reaction to a similar extent.^48^ The effect of aromatic rings was explored by Christie et al., who introduced phenyl rings directly on the nitrogen atom of maleimides (**1c**) to allow direct delocalization of the nitrogen lone pair and thus enhance the electrophilicity of the carbonyl and favor the hydrolysis reaction.^49^ In the same context, Kalia et al. explored the effect of the aminomethyl (*o*-CH_2_NH_2_) in the *ortho* position of *N*-arylmaleimides (**1d**), which further increased the rate of hydrolysis due to a combination of both resonance and base catalysis effects; the latter was compared with a derivative bearing an amino group directly attached to the aromatic ring (*o*-NH_2_) which produced a thiol conjugate that is highly resistant toward hydrolysis.^50^ In the end, the research groups of Tumey^45^ and Wagner^51,52^ developed PEGylated and acetal-based maleimides (**1e**), whose corresponding rings were hydrolyzed thanks to the ability of the substituents to coordinate water molecules.

To overcome the limitations discussed above, a new class of maleimides, namely, ″next-generation maleimides” (NGMs), was developed. The employment of NGMs piloted the development of pyridazinediones (**PDs**) and 5-methylene pyrrolones (**5MPs**) as reagents for tunable cysteine-selective bioconjugation reactions. Over the past decade, the group of Chudasama explored the employment of dibromo- and bromopyridazinediones as tools for the bioconjugation of cysteines in the development of ADCs,^30,53,54^ whose characteristics will be further discussed in paragraph 3.3. The most interesting feature of these systems is their chemical inertia toward ring hydrolysis, the most important drawback in the application of reversible maleimide-based bioconjugates (Scheme 3).

This important characteristic allowed the design of reversible cysteine-selective linkers, whose bioconjugation products undergo a retro-Michael reaction. Chudasama explored such an aspect by using *N,N*-diethyl pyridazinedione (**PD**, **6**) in a dynamic study for the bioconjugation of green-fluorescent protein (GFP) and the Fab fragment of monoclonal antibody Trastuzumab. The authors performed a preliminary evaluation of the PD platform using Boc-Cys-OMe to act as a model for thiol conjugation, highlighting first a rapid conversion into the conjugated product and then a slow deconjugation process under physiological conditions (Scheme 4). The system was then tested in a real bioconjugation of GFP and Fab fragment cited above, and the products were first incubated for 7 days under physiological conditions and then analyzed using LC-MS. As a comparative model, an *N*-methylmaleimide-based bioconjugate was produced and tested under the same conditions. MS spectra showed a significant release of the PD moiety from PD-based bioconjugates, in sharp contrast with maleimide-based ones, which were completely converted into succinamic acid derivatives and thus stable to retro-Michael deconjugation. The authors also compared PD-based and maleimide-based bioconjugates toward thiol-exchange with blood thiols, resulting in no transfer of PD to either GSH or HSA, in contrast with the maleimide-based system. This evidence has suggested the use of a PD linker as a novel platform for the construction of reliable bioconjugates that provide a slow release of payload without concern over cleavage in off-target sites.

**Scheme 4:**
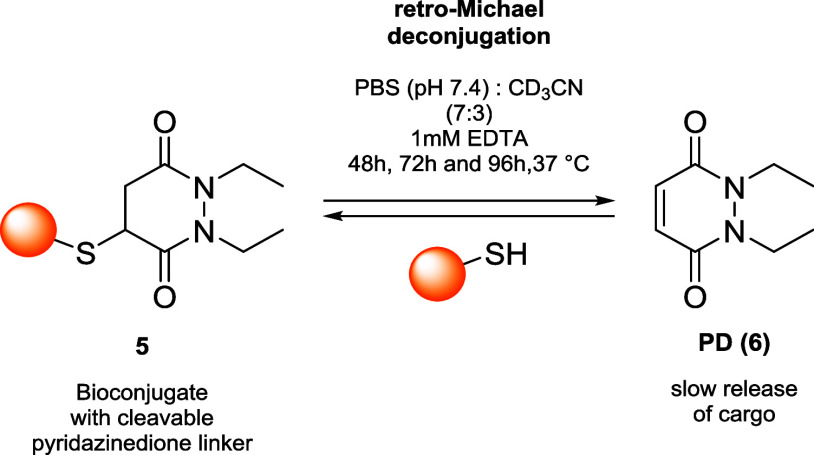
Deconjugation strategy for thiol release by the thiol-PD (**5**) bioconjugate

The **5MP** systems can also be used as an alternative to maleimides for the reversible and specific bioconjugation of cysteines. Even though they are very similar, the replacement of the carbonyl group of the maleimide with a methylene makes 5MP stable toward ring-opening hydrolysis even at pH 9.5, as reported by the group of Zhou (Scheme 5).^55^ For instance, the suppression of the ring-opening hydrolysis pathway leads to the construction of fully reversible bioconjugates that release the payload via retro-Michael- or thiol exchange reactions. Moreover, unlike maleimides, the thiol-Michael reaction on the exocyclic double bond does not generate a stereocenter, thus simplifying product analysis. Zhou and co-workers pointed out that the retro-Michael reaction is triggered at pH 9.5, which is not compatible with intracellular and some *in vitro* applications. However, the authors highlighted the presence of a thiol exchange mechanism at neutral pH, demonstrated by incubating a 5MP-protein bioconjugate with excess GSH at pH 7.5. UPLC-MS analysis of the reaction mixture revealed the complete conversion 5MP-protein bioconjugate into the 5MP-GSH adduct, demonstrating that an excess of thiol can regenerate the native protein under mild conditions.

**Scheme 5:**
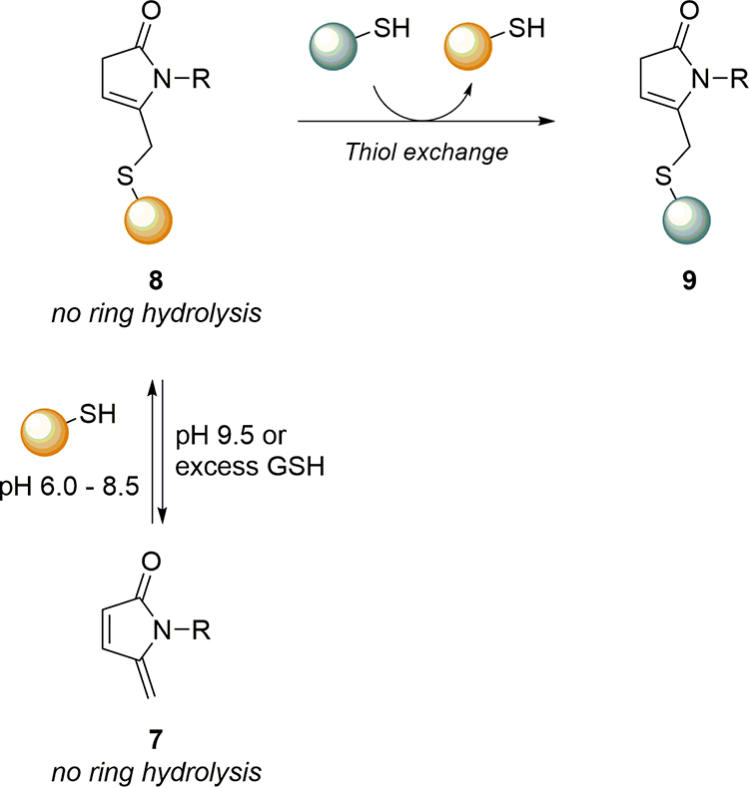
Reversible Thiol Conjugation with **5MP** (**7**) and Release through Thiol Exchange Reaction

### 2.2 Enones, Ynones, and Acrylates

Enones, ynones, and substituted acrylates were employed for the covalent modification of different targets by exploiting the nucleophilicity of noncatalytic cysteines. Among the various reagents employed for this purpose, 4-substituted cyclopentenones have been studied by the group of Yin and co-workers.^56^ Unlike other reagents, 4-acetoxy cyclopentenone emerged as a highly specific labeling platform for cysteine bioconjugation at nearly physiological pH, endowed with excellent reactivity and highly tunable stability toward deconjugation. This last characteristic is strictly related to its structure, as 4-substituted cyclopentenone can be the direct precursor of cyclopentadienone, an unstable nonaromatic compound that tends to dimerize.^57^ Thanks to this structural feature, 4-substituted cyclopentenones and their bioconjugates are highly stable and do not undergo direct retro-Michael deconjugation. The authors hypothesized a mechanism where 4-acetoxy cyclopentenone **10** fast reacts with the target thiol, giving the conjugated adduct **11**, which is involved in rapid β-elimination of AcOH (Scheme 6). The newly formed 4-substituted cyclopentenone **12** is a highly stable bioconjugate since β-elimination would lead to unstable cyclopentadienone formation. In fact, β-elimination to regenerate the parental thiol can occur only from adduct **13**, which is obtained after the addition of an extra Michael donor (*e.g.*, an extra thiol).

**Scheme 6:**
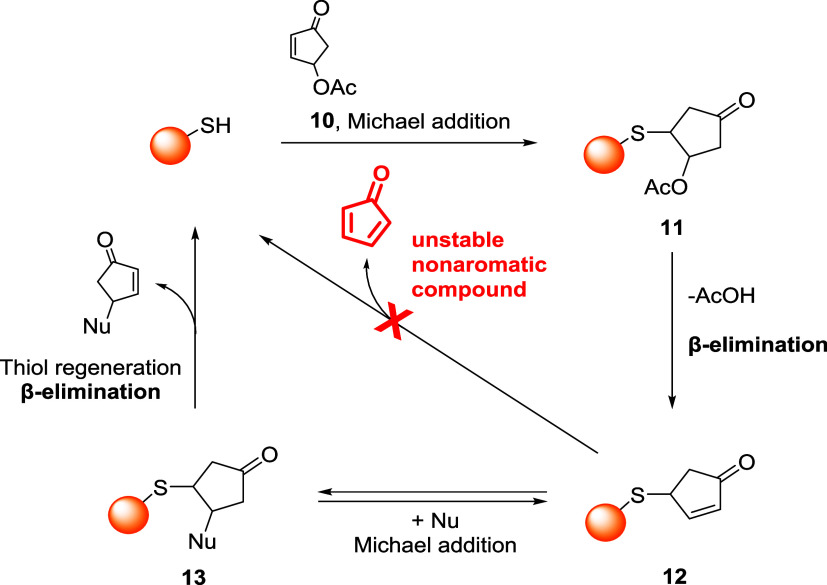
Reversible Thiol Conjugation by Means of 4-Acetoxy Cyclopentenone **10**

The proposed mechanism was confirmed by carrying out the bioconjugation in PBS buffer (pH 7.4) between **10** and a model peptide containing all naturally occurring nucleophilic amino acids. MS analysis showed the formation of adduct **11** after 2 min, before proceeding to completion with the formation of **13** in 12 min. UBXD—a model protein containing one cysteine and 6 lysines—was tested for selective cysteine bioconjugation with **10** in PBS buffer at different pH values. LC-MS^2^ analyses on the fragmented peptides obtained after trypsin digestion highlighted the selective conjugation on the single cysteine rather than on lysines, whereas a negligible impact on the structure or conformation of UBXD was observed by UV–vis and circular dichroism spectra. The same reaction was carried out with cyclopentenone and maleimide, leading to adduct decomposition via retro-Michael and ring-opening hydrolysis, respectively. The reactivity of **10** was also tested toward different protein targets containing both accessible and hindered cysteines. In any case, **10** showed a high reactivity compared to iodoacetamide (**IAA**), also achieving multiple functionalization in the case of MERS C3-like protease, where 7 residues out of 8 were successfully modified. The authors eventually evaluated the application of reversible bioconjugation of a protein by means of compound **10**. For this purpose, the selected biological target was EV 71 3C, a protease whose activity relies on a cysteine residue in the active site.^31^ Reaction trials performed by reacting 3C protease and **10** or IAA, which inactivated the enzyme, highlighted how the catalytic activity was restored upon treatment with β-mercaptoethanol (βME) in exchange for cyclopentenone, whereas the IAA-modified protein remained inactive.

Linear α,β-unsaturated carbonylic compounds have also been investigated for the bioconjugation of cysteine residues. The work carried out by the group of Wang and Che^58^ started from the study by Tsou, which explored the irreversible inhibition of Epidermal Growth Factor Receptor (EGFR) kinase by alkynoic amides.^59^ Wang, Che and co-workers exploited the formation of vinyl sulfide linkages to study the reversible conjugation of cysteine by means of alkynoic amide **14**, ester **15**, and ynones **16–20** (Scheme 7). The authors first tested the reactivity of **14** with a model peptide at different pH values, confirming the evidence reported in other works: since the reaction rate increases at higher thiolate concentrations, the best performances were obtained at higher pH values, *i.e.*, pH 8.0 and pH 9.0. Among the remaining linkers tested on the same peptide at pH 8.0, **16** and **17** gave the best performances, reaching 100% conversion in 30 min. The reaction between model *N*-Boc cysteine ethyl ester and linkers **14–19** in different solvent systems, at different pH values, allowed us to evaluate the stereochemistry of the vinyl sulfide adducts. The results obtained were consistent with previously published studies, where it was observed that *Z/E* ratios for the conjugation of protected amino acids with electron-deficient alkynes are influenced by the operative solvent systems.^60^ In any case, the *Z*-isomer is predominant and, in this case, it was found that the weaker the electron-withdrawing ability of the activating group, the lower the *Z/E* ratio observed. Moreover, the authors observed that the vinyl sulfide linkage could be cleaved by treatment with thiols with an addition/elimination mechanism. It was also pointed out that an accurate choice of substituents on the alkynoic moiety allows for control of the cleavage of the vinyl sulfide adduct. In fact, treatment of vinyl sulfide linkages **14–19** with thiophenol afforded the unmodified peptide upon formation of the thioacetal only in the case of adducts **16** and **17** (Scheme 7). This behavior was explained by the authors by taking into account the rate constants of the reactions: since generally *k*_1_ ≫ *k*_2_, it is unlikely for those linkers with low *k*_1_, *i.e.*, **14**, **15**, **18**, and **19**, to undergo a second thiol addition upon formation of the corresponding vinyl sulfides.^61^ Therefore, the formation of alkynoic amide-, alkynoic ester-, and internal alkynone-modified peptides is less favorable, which accounts for their stability toward deconjugation in the presence of excess thiol. This ynone-based thiol conjugation has found applications in the macromolecular chemistry of self-healing hydrogels, such as in the example reported by Fan et al. in 2020, through the introduction of dynamic thiol-alkynone double addition cross-links.^62^ These hydrogels were synthesized from multiarm PEG thiols and small-molecule alkynone cross-linkers, producing a stable injectable formulation for therapeutic applications. In their work, the authors showed that when the hydrogel is forced through a syringe, the applied shear stress temporarily disrupts the dynamic cross-links, allowing polymer chains to slide past each other. As a result, the material behaves like a viscous fluid under stress, enabling injections through the needle.

**Scheme 7:**
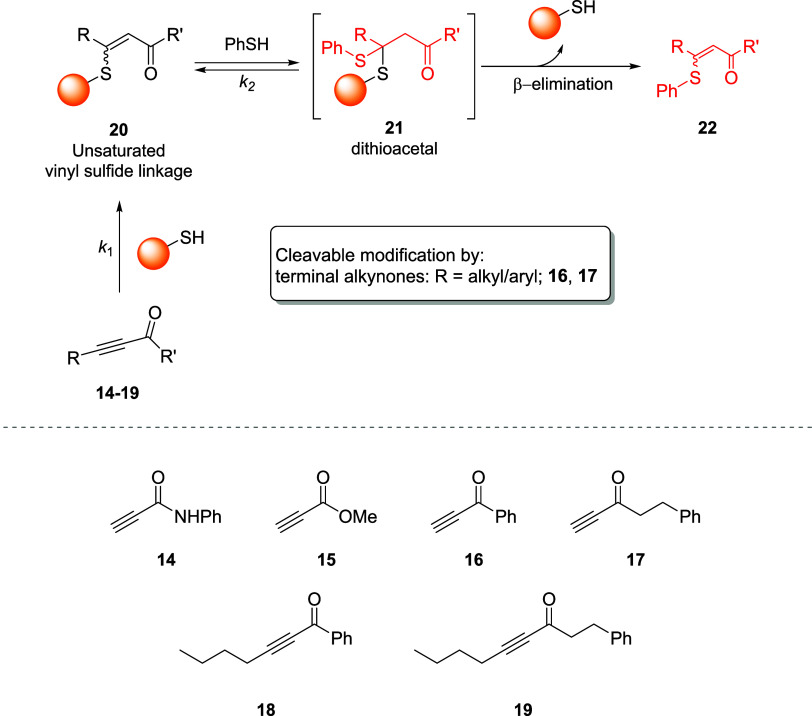
Reversible Conjugation of Cysteines by the Formation of Vinyl Sulfide Linkages with Amide **14**, Ester **15**, and Ynones **16–19**

The importance of kinase inhibition for chronic diseases and cancer therapies led to the discovery of several irreversible kinase inhibitors endowed with an acrylamide scaffold. In addition, kinases are the second largest family of drug targets with 518 members—whose majority have an accessible cysteine residue close to the active site of the enzyme—and nowadays seven acrylamide-based cysteine-targeted kinase inhibitors have been approved by the FDA for the treatment of cancer.^63^ However, these compounds can react irreversibly with GSH and other hyper-reactive cysteines, resulting in an undesired off-target effect. Moreover, the irreversible inhibition may result in covalent adduct formation whose potential toxicological effects cannot be predicted by currently used preclinical models.^32^ In contrast with acrylamide, in the 1960s, 2-cyanoacrylates showed complete reversibility upon reaction with simple thiols at physiological pH.^64^ In fact, in addition to the ester moiety, the nitrile substituent is a strong EWG that facilitates both Michael and retro-Michael processes by increasing, respectively, both the electrophilicity of the β-carbon and the acidity of the protons in the α position of the conjugated adduct. In this context, the group of Taunton designed and synthesized three Michael acceptors (*i.e.*, a methyl acrylate (**23**), an acrylonitrile (**24**), and a 2-cyanoacrylate (**27**)) to evaluate the reversibility of Michael addition with βME (Scheme 8).^65^

**Scheme 8:**
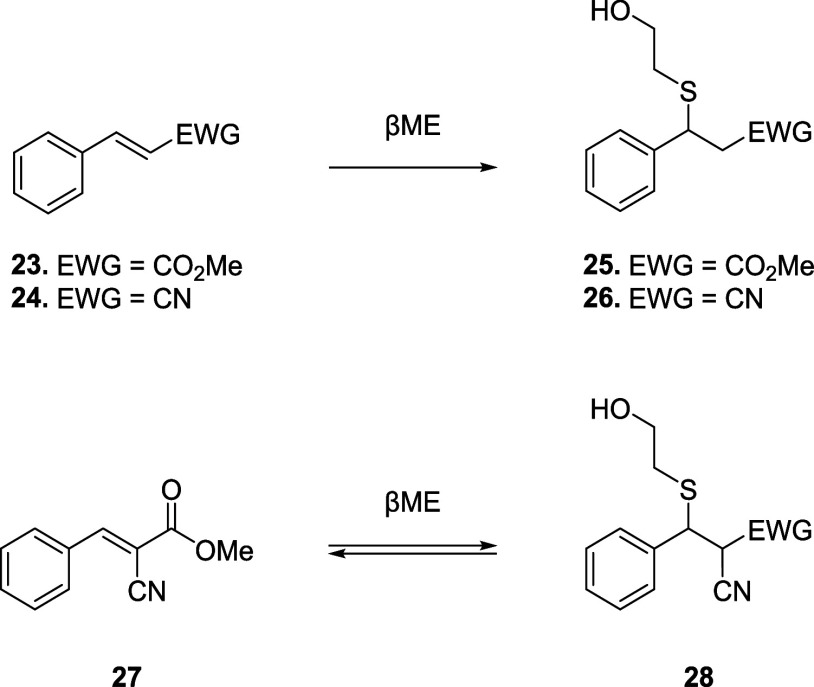
βME Addition of Methyl Acrylate **23**, Acrylonitrile **24**, and Methyl Cyanoacrylate **27**

UV–Vis and ^1^H NMR analyses showed the formation of stable thiol adducts from **23** and **24**, in sharp contrast with **27**, which showed complete reversibility upon dilution. The authors eventually explored the properties of electrophilic pyrrolopyrimidines for the inhibition of the *C*-terminal kinase domain (CTD) of p90 ribosomal protein S6 kinase RSK2. These compounds feature acrylate, acrylonitrile, cyanoacrylate and cyanoacrylamide scaffolds (Figure 3), and among them, only cyanoacrylate and cyanoacrylamide derivatives did not afford LC/MS detectable adducts, resulting in reversible conjugation of Cys436 in the RSK2 active site. Moreover, the inhibition with *N*-isopropyl cyanoacrylamide derivative **32** was long-lived (*t*_1/2_ = 245 min) but fully reversible compared to derivative **31** (*t*_1/2_ = 42 min).

**Figure 3:**
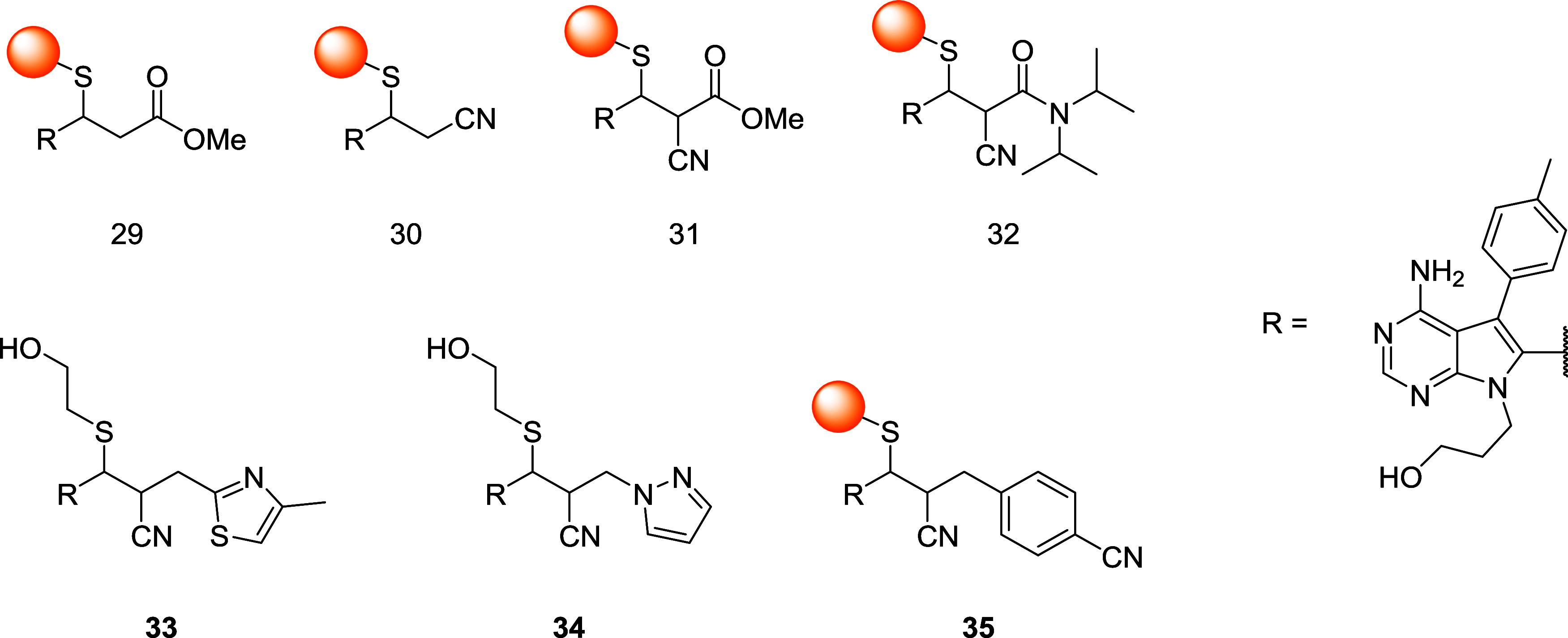
Structures of pyrrolopyrimidine-based thiol adducts **29–35**.

These findings can be explained based on the results from the stabilization of the thioether adduct within the active site of the enzyme. In fact, the authors demonstrated that as soon as the protein is unfolded by the addition of SDS or guanidine, the thioether adduct can be cleaved, regenerating the unmodified cyanoacrylamide. In the context of RSK inhibition, the same group developed a library of 10 acrylonitriles substituted in the α position with aryl or heteroaryl EWG and in the β-position with cyclopropane or pyrrolopyrimidine to better elucidate the structural requirements for reversibility.^66^ After conjugation with βME, NMR or LC/MS analyses highlighted an immediate β-elimination with methylthiazole (*t*_1/2_ < 1 min) as a substituent (**33**), whereas the pyrazoyl adduct (**34**) exhibited a more “irreversible” character (*t*_1/2_ > 58 h). Among the tested compounds, although cyclopropyl derivatives exhibited 2–3 times slower elimination rates, the results given by NMR analyses demonstrated that the pyrrolopyrimidyl scaffold is not essential for reversibility. Computational studies also allowed the evaluation of the proton affinity in aqueous solution for the α-carbanion of each βME/adduct. The most interesting result is the linear correlation of calculated proton affinity and rate constants in a Brønsted-type plot, allowing the prediction of deconjugation rates even for novel and uncharacterized acrylonitriles. In the kinase assay, all acrylonitriles exhibited inhibition of the RSK2 *C*-terminal kinase domain, with derivative **33** being the most potent (IC_50_ = 12 nM) and *p*-cyanophenyl compound **35** being the least potent (IC_50_ = 770 nM). The formation of a covalent thioether adduct was first observed by a decrease in potency of all acrylonitriles against Cys to Val mutant and then confirmed by X-ray structure.

Cyanoacrylate reversible linkers have recently been exploited for different applications. Among them, the group of Woolley employed the cyanoacrylate scaffold to induce helix folding/unfolding in peptides and proteins.^67^ This was possible thanks to the introduction of photoswitchable functionalities in the molecule, such as an azobenzene moiety. The linker was designed with two cyanoacrylate moieties in the *para* positions of the azobenzene to make it selective toward peptide sequences with properly spaced Cys residues (Scheme 9).

**Scheme 9:**
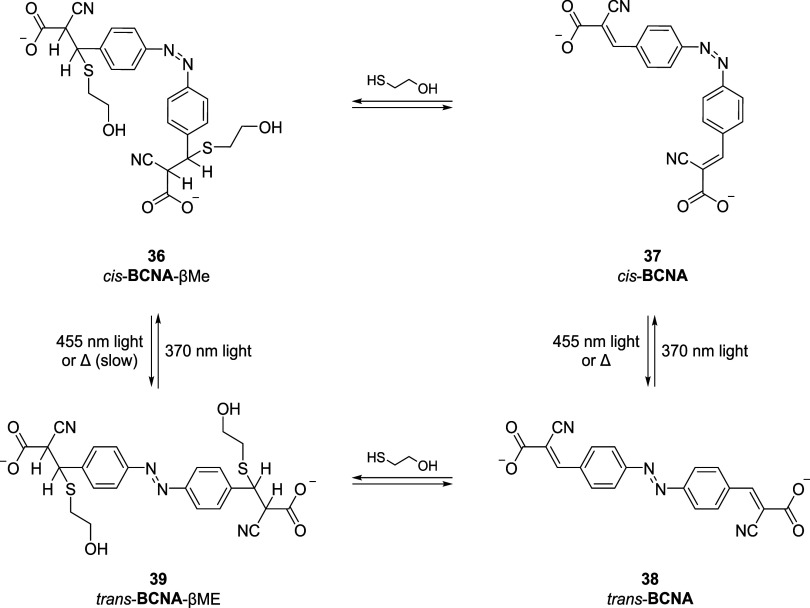
Photoinduced *cis* to *trans* Isomerization of *cis*-BCNA-βME (**36**) to *trans*-BCNA-βME (**39**), Which May proceed via Dissociation and Reassociation of βME

The authors first examined linker reactivity toward monothiols by UV–Vis titration, which also confirmed reversibility upon dilution.^66,68^ They then incubated the linker with peptides containing two cysteine residues, selecting sequences with S–S distances of 13–14 Å. The Z domain (i,i+7) and SS7L, with S–S distances of 10–15 Å, gave the strongest binding, followed by Z domain (i,i+11), all more reactive than βME (∼1 mM). Finally, the authors also observed that UV irradiation at 370 nm of the SS7L and Z domain (i,i+7) adducts reduced helicity by *trans–cis* isomerization of the azobenzene, inducing peptide conformational changes.

Cyanoacrylates have also been studied in the context of thiol-mediated uptake (TMU), a process regulated by covalent cascade exchangers (CAXs), widely explored by the group of Matile.^69–71^ These compounds have been explored not only for the delivery of different substrates to cytosol, such as genes, proteins, and even quantum dots, but also for the inhibition of cell motility and entry of lentiviruses.^23^ Tetrel-centered CAXs emerged as effective TMU inhibitors, in contrast with chalcogen-centered CAXs, such as 1,2-dithiolanes, which readily penetrate cells. Unlike chalcogen-centered CAXs that walk through a disulfide arrays^72^ by means of thiol/disulfide exchange reaction (see Section 6), tetrel-centered CAXs exchange exclusively with thiol/ate arrays.^71^ The authors also reported that the moderate inhibition activity of tetrel-centered Michael acceptors is even increased in dimeric Michael acceptors, identified as strong TMU inhibitors.^73^ This process proceeds through subsequent thiol-Michael and retro-Michael reactions: in the case of monomeric tetrel-centered CAXs (**40**) (Scheme 10a), one electron-poor double bond undergoes conjugation and deconjugation processes, “hopping” (Scheme 10a′) along thiol/ate arrays without permanent contact during the entire cascade; on the other hand, dimeric tetrel-centered CAXs (**41**) (Scheme 10b) react with both electron-poor double bonds in subsequent conjugation and deconjugation processes, resulting in “thiol/ate walking” without losing covalent contact with the arrays (Scheme 10b′). The authors also explored γ-thiolactones as tetrel-centered CAXs thanks to the well-established exchange reaction between thioesters and thiolactones with thiol/ates. The increasing interest in these CAXs is justified by their application in protein folding and DNA transcription mimicry and in the biosynthesis of some natural compounds. Unlike Michael acceptors, γ-thiolactones “hop” along the thiol/ate arrays (Scheme 10c′′) rather than “walking” on disulfide arrays (Scheme 10c′) due to the ring-closing reaction that competes with thiol exchange (Scheme 10c). On the other hand, γ-thiolactones dimers (**42**) could walk on thiol/ate arrays thanks to the ring-closing reaction mechanism (Scheme 10d) that allows partial detachment of the molecule, thus resulting in walking maintaining constant covalent contact with the array, which translates into TMU inhibition (Scheme 10d′).

**Scheme 10:**
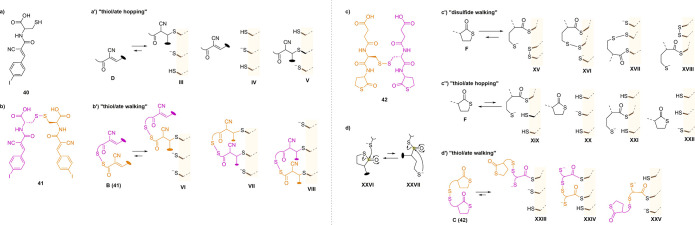
Reactivity of Dimers **41** and **42** ^a^

a Left: (a′) “thiol/ate hopping” mechanism without permanent contact with the thiolate array; (b′) “thiol/ate walking” mechanism along the thiolate array with permanent contact. Right: (c′) unfavored “disulfide walking” mechanism with permanent contact with the thiolate array; (c′′) “thiol/ate hopping” mechanism without permanent contact with the thiolate array; (d) ring-closing mechanism that favors “thiol/ate walking” mechanism (d′) along the thiolate array with permanent contact.

To evaluate inhibitory performance, the authors synthesized cyanoacrylate and γ-thiolactone dimers (**41**, **42**) and their thioacetal analogues (**43**, **44**) (Figure 4).

**Figure 4:**
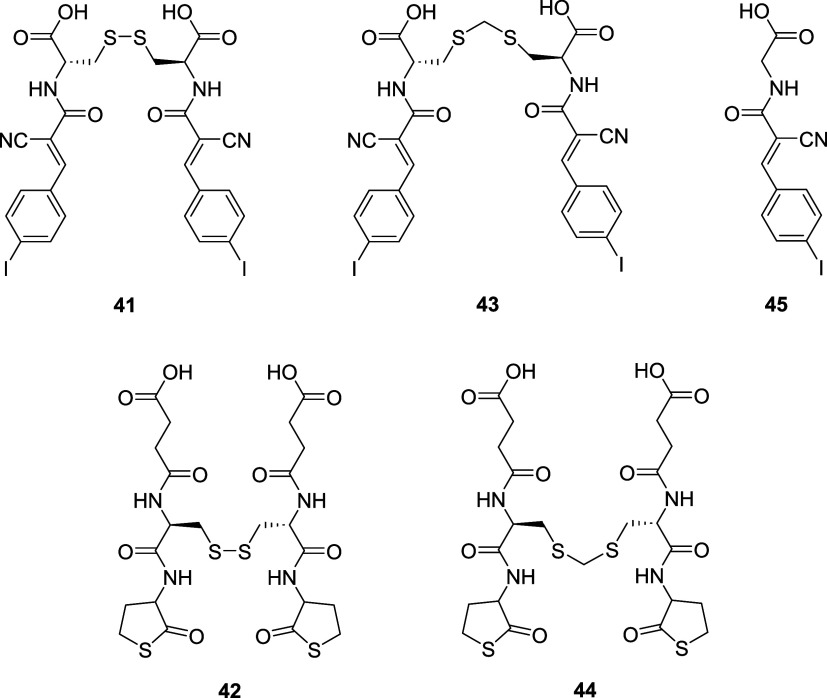
Structures of dimeric CAXs **41** and **42**, their corresponding thioacetal derivatives **43** and **44**, and monomeric **45**.

TMU inhibition was assessed via a fluorescence decrease with an epidithiodiketopiperazine penetrative fluorescent probe. Dimer **41** showed an impressive TMU inhibition (IC_50_ = 4.7 ± 0.5 μM) as well as the analogous thioacetal **43** (IC_50_ = 8.6 ± 0.9 μM), thus confirming the presence of a Michael addition cascade with negligible contributions from disulfide exchange. Monomer **45** was far weaker, showing an IC_50_ = 90 ± 20 μM, consistent with the hypothesis of its “hopping” mechanism. γ-Thiolactone dimer **42** and its corresponding thioacetal analogue **44** gave similar but weaker profiles compared to those of **41** and **43** (IC_50_ = 25 ± 3 μM for dimer **42**). FITC-labeled assays on HeLa Kyoto cells further revealed that Michael acceptor dimers mainly remained at the cell surface and acted as inhibitors, in contrast with monomers that enhanced uptake, and γ-thiolactones, which showed limited activity in both directions.

The acrylate scaffold has also been studied by Jiang and colleagues, who designed a ratiometric fluorescent probe, namely **RealThiol** (**RT**) (Figure 5), for the quantitative real-time imaging of GSH in living cells.^74^ A ratiometric fluorescent probe is a sensing tool that measures a target substance by quantifying the ratio of fluorescent intensities at two different wavelengths.

**Figure 5:**
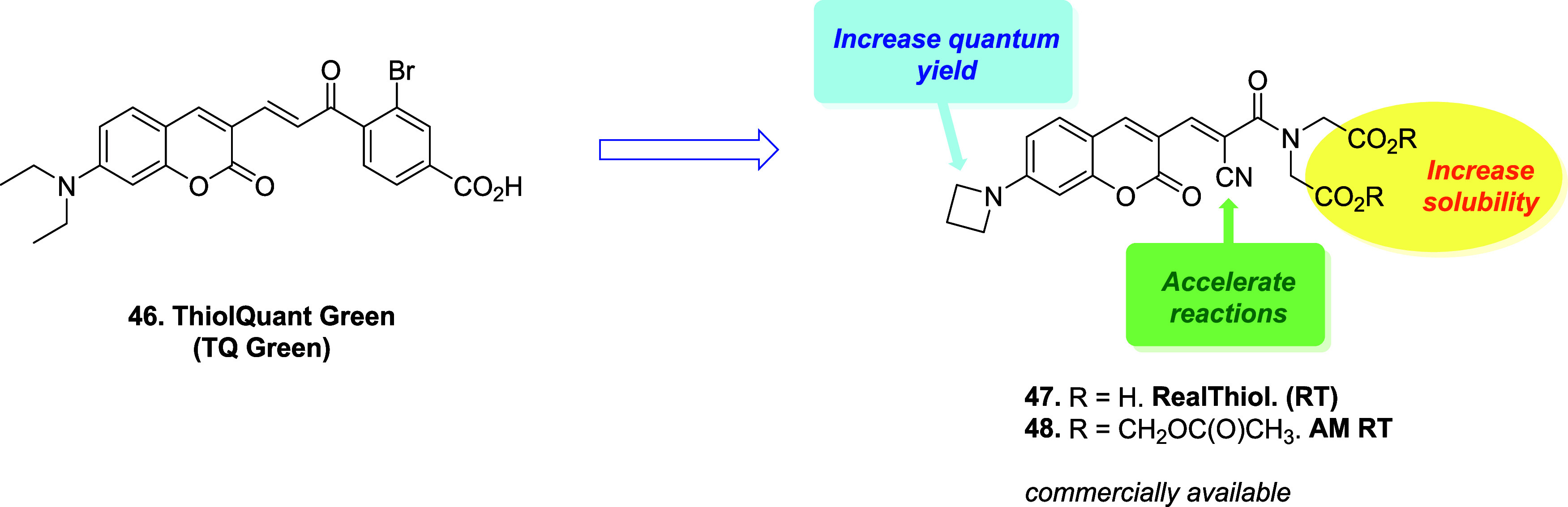
Rational design of ratiometric fluorescent probes **47** and **48** from **46**.

The realization of the **RT** probe builds on a previously published system, **ThiolQuant Green** (**TQ Green**),^75^ but it offers higher *K*_D_ (3.7 mM vs 1.6 mM) and higher quantum yields (2.90 vs 0.94, respectively, and 86.0 vs 0.59 for the corresponding GSH adducts). **TQ Green** is a modular system that consists of 3 main moieties: a 7-amino coumarin portion with fluorescence properties suitable for confocal imaging; a substituted aromatic ring, which extends the absorption wavelength of the coumarin scaffold; and a central part, which features the Michael acceptor portion. The performance of **TQ Green** increased in **RT** by replacing the diethylamino substituent with an azetidine, which improves photostability and quantum yields. Moreover, the presence of the α-cyano substituent and the replacement of the ketone with an amide balanced the kinetics of both the direct and reversed Michael reaction. In addition, the introduction of carboxylic groups ensures aqueous solubility, thus reducing the interactions with hydrophobic cellular structures. To improve cell permeability, the authors converted **RT** into the acetoxymethyl ester (**AM RT**) derivative, which is readily converted into the corresponding acid by the esterases. Moreover, as mentioned above, quantum yields in **RT** are increased with respect to **TQ Green**, and this was made possible by replacing the diethylamino substituent in position 7 with an azetidine, as reported by Lavis.^76^ The reversibility and GSH sensitivity of **TQ Green** were examined through controlled reactions with varying GSH concentrations. Reaction with excess GSH produced characteristic spectral shifts (decreased absorbance at 488 nm and increased absorbance at 405 nm), which reverted upon GSH depletion, demonstrating reversibility. Similar results across a range of GSH concentrations confirmed an isosbestic point at 426 nm, indicating clean interconversion without irreversible side reactions. The fluorescence ratio *F*_405_/*F*_488_ responded selectively to GSH over other thiols and reactive oxygen or nitrogen species under physiological conditions, demonstrating the selectivity of the probe toward GSH. **RT** was successfully employed for the real-time quantitation of GSH in living cells by means of lysate-based liquid chromatography–mass spectrometry (LC-MS). After treatment with H_2_O_2_ (500 μM) to induce oxidative stress, GSH level in HeLa cells decreases from 5.0 to 4.1 mM, which was then re-established after addition of GSH ester (100 μM), demonstrating the ability of the **RT** probe to monitor real-time GSH fluctuations. For further applications of this probe, see ref 74.

The coumarin scaffold was also employed for the labeling of human cellular retinol-binding protein II (hCRBPII) by Geiger and Borhan.^77^ For such a purpose, the author used a fluorescent probe (**CM1V**, **49**) featuring an α,β-unsaturated aldehyde attached to a 7-diethylamino coumarin scaffold (Scheme 11). The main feature of this system is its ability to switch between a fluorescent (ON) and a dark (OFF) state upon reaction with the α,β-unsaturated moiety. The existence of the dark state is caused by the disruption of the extended conjugation, which is responsible for fluorescence in such systems, and it is crucial in single-molecule localization microscopy (SMLM) techniques that can image biological structures at the molecular scale. Other fluorescent probes, *e.g.*, red carbocyanine dyes, act in the same way, and they will be discussed later in this paragraph. The authors engineered the binding cavity of hCRBPII with a cysteine residue to promote Michael addition and hence photoswitching to the dark state of **CM1V** (hCRBPII-CM1V conjugate). Additionally, the engineered cysteine is properly oriented toward a proximal lysine residue, which anchors the probe by Schiff base formation with the aldehyde moiety of **CM1V**, thus facilitating C–S conjugation. The authors also synthesized an *N*-butyl Schiff base (**CM1V-SB**, **50**) and protonated *N*-butyl Schiff base derivatives (**CM1V-PSB**, **51**) to investigate the spectroscopic properties of the system in organic solvent upon reaction with βME, as a model of the final bioconjugate (Scheme 11).

**Scheme 11:**
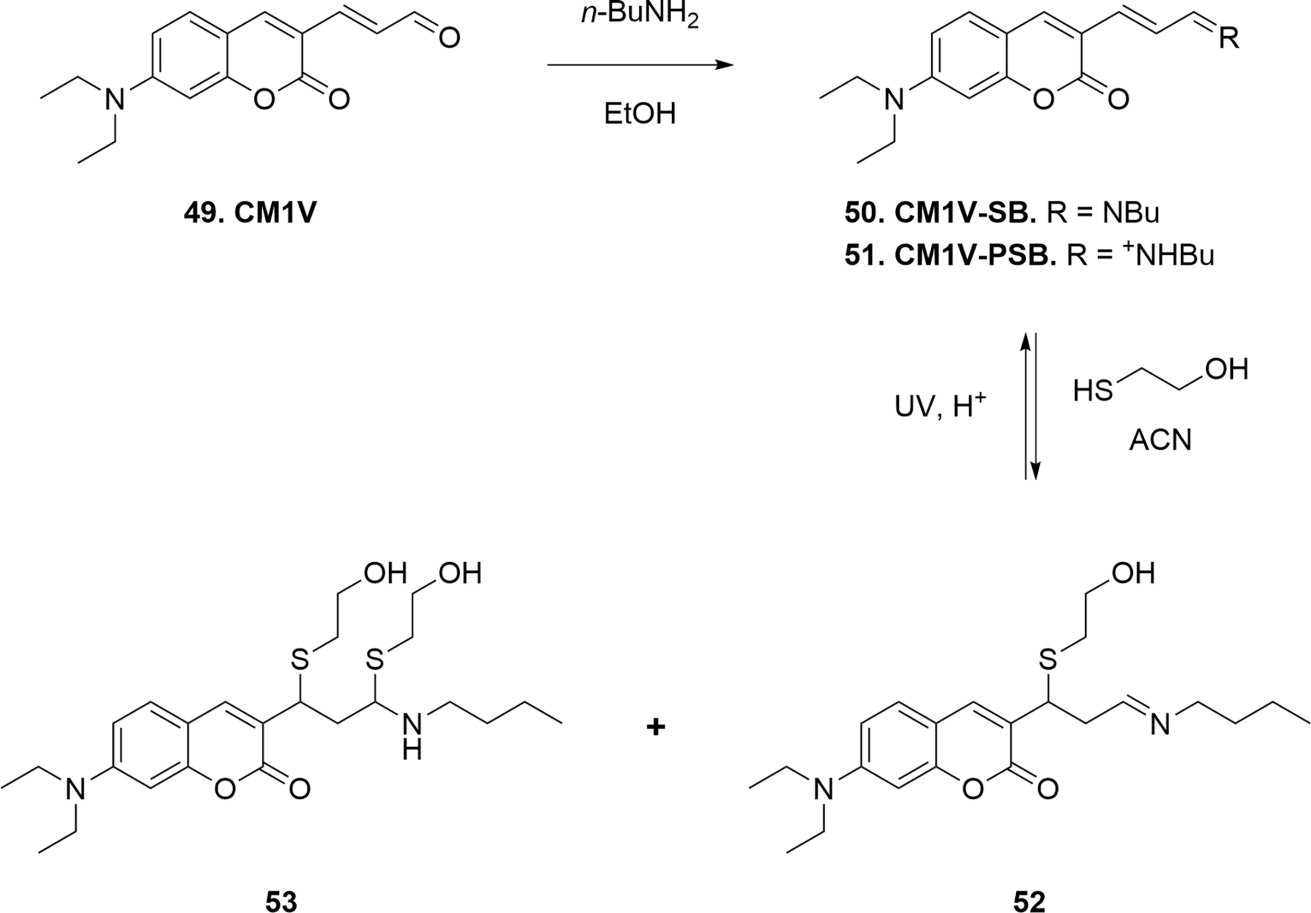
Synthetic Strategy for the Preparation of Model Compounds **50** and **51** and Reaction with βME for the Evaluation of Their Spectroscopic Properties

Both **CM1V-SB** (λ_abs_ = 432 nm) and **CM1V-PSB** (λ_abs_ = 510 nm) were tested independently toward βME, pointing out a blue shift in the absorption spectra (∼370 nm) upon conjugation, as expected for these systems. UV irradiation reverted the thioether adducts **52** and **53** back to the parent **CM1V-PSB**, which is red-shifted to ∼500 nm. The next phase involves engineering a protein, hCRBPII, to bind with **CM1V**. Mutations were introduced to prepare different protein variants. One of the first tested, called mutant M1, includes a lysine at position 108, but no reactive cysteine. When M1 is incubated with **CM1V**, both SB (425 nm) and PSB (550 nm) forms are observed, showing successful binding but without covalent attachment to a cysteine. To enable that covalent attachment, a second mutant, M2, is prepared by introducing a cysteine at position 51. In this case, a Michael addition occurs between dye and cysteine, causing a blue shift to 395 nm and indicating loss of conjugation. At acidic pH, PSB formation was favored in M2, but unlike the SB form, it does not revert thermally after UV exposure. This is likely due to a different dye orientation or reduced reactivity of cysteine under acidic conditions. To improve the stability, a third mutant, M3, was designed by restoring the native glutamine at position 4. UV–Vis spectra of M3 showed both PSB (550 nm) and Michael adduct (390 nm) signals, implying incomplete but functional conjugation. Structural analysis suggested this is due to alternative conformations within the protein, only some of which bring the reactive groups close enough for conjugation. Finally, M3 was shown to undergo reversible ON/OFF switching with light: the fluorescent PSB state (ON) can be converted to a nonfluorescent cysteine-bound state (OFF) and back again (Scheme 12). This switch remains efficient over at least 20 cycles with minimal fluorescence loss, demonstrating strong photostability, an important feature for advanced microscopy techniques.

**Scheme 12:**
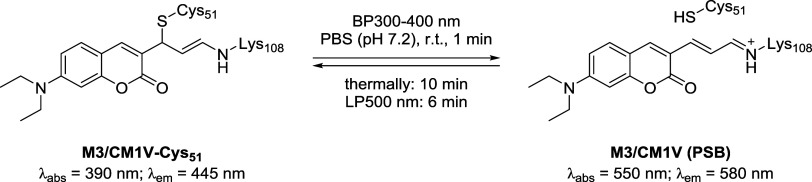
Photoactivated Reversible Cysteine Deconjugation from the **M3/CM1V-Cys**_**51**_ Adduct

Cyanine dyes are another class of labeling probes that show a switchable fluorescent state upon irradiation in the presence of a thiol. These compounds feature a polymethine chain that is a conjugated spacer between two heteroaromatic moieties, making cyanines intrinsically fluorescent, unlike the above-mentioned **CM1V** linker. Although the employment of cyanine dyes in association with nucleic acids for their detection is dated to early 90s and the photoswitching phenomenon had already been observed for these compounds, it was only in late 00s when Dempsey reported the reversible photoswitching of **Cy5** (**54**) dye upon reaction with a simple thiol like βME.^78^ Although Dempsey observed the conversion of **Cy5** into a dark state, the mechanism of this process has been unraveled recently by the group of Cosa.^25^ To formulate a mechanism, the authors had to take into account: (*i*) the role of the triplet excited state of the dye; (*ii*) the photostabilizing role of aliphatic thiols which quench the triplet excited state via photoinduced electron transfer (P*e*T); (*iii*) the presence of a back electron transfer (B*e*T) competing process, due to thiyl radical-assisted triplet-to-singlet intersystem crossing (ISC).

The authors first explored the photoswitching of **Cy5** in the presence of increasing concentrations of iodide, a catalyst for excited singlet-to-triplet ISC. According to the results obtained by Zhuang,^79^ the group of Cosa observed a linear correlation between the rate of **Cy5** photoswitching and the concentration of iodide. The mechanism for Cy5-thiol adduct formation relies on a radical combination. After P*e*T (*k*_P*e*T_^3^ Scheme 13), a new geminate radical pair (GRP) between the thiyl radical and the reduced Cy (**Cy** ^**–•**^) is formed, leading to the formation of Cy5-thiol adduct in small yields upon thiyl radical-assisted triplet-to-singlet ISC. The authors also considered other pathways for the formation of the dark state, such as **cis-Cy5**, **Cy5^+^** ^•^, and **Cy5^–^** ^•^, but transient absorption spectroscopy revealed micro- to submillisecond lifetimes, incompatible with the long-lived observed dark state. Transient species detected with Cy5B, the locked analogue unable to photoswitch, showed similar lifetimes, further excluding their contribution. Uncaging of the Cy5-thiol adduct was reported to occur either by direct irradiation at short wavelengths (337–532 nm)^80^ or indirectly through a nearby **Cy3** fluorophore.^79^ The authors hypothesized that regeneration of the GRP occurs via homolytic cleavage of the Cy5-thiol bond, regardless of whether the process takes place via a direct or indirect mechanism. Uncaging studies also revealed that the rate of **Cy5** recovery scales linearly with excitation power even at 647 nm, indicating a one-photon process, with a nonzero intercept, pointing to a parallel thermal uncaging reaction. The rate of this thermal process increased with decreasing ionic strength at pH 8.0 without affecting the slope of von versus power, suggesting a rate-limiting step involving a positively charged species, attributed to adduct protonation for activation of the leaving group. Consistent with this hypothesis, a linear correlation between the rate of **Cy5** restoration and pH, with slope 1 confirmed an acid-catalyzed bimolecular thiol elimination. High pH values enhance thiolate concentration, P*e*T, and adduct formation but slow thermal uncaging, whereas lower pH accelerates uncaging, underscoring the pronounced sensitivity of the Cy5-thiol system to pH.

**Scheme 13:**
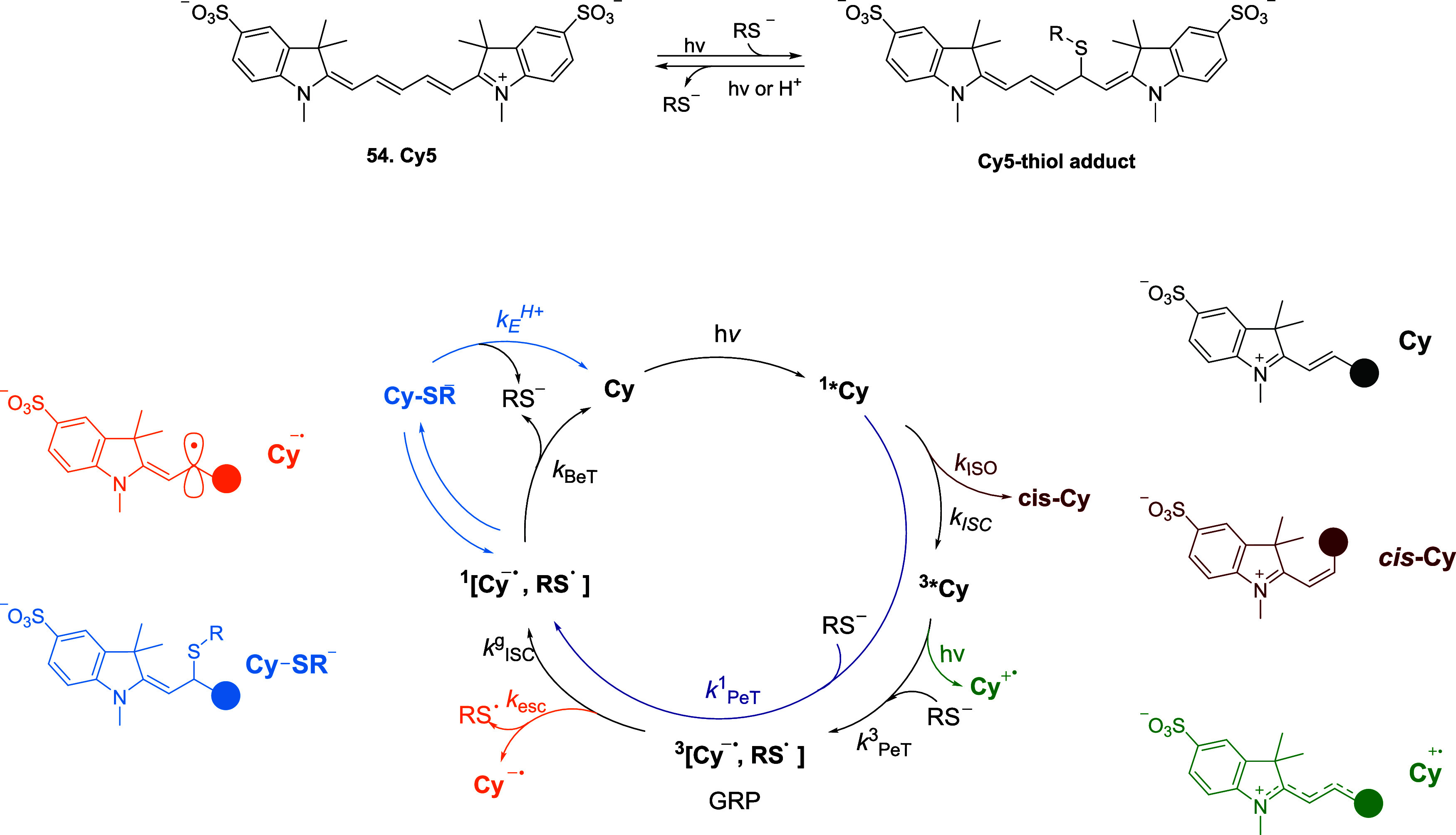
Reversible Mechanism for the Photoinduced Thiol Addition to the **Cy5** Probe (**54**)

Another interesting example of photochemical-assisted Michael reaction for the conjugation of thiols, including peptides and proteins, is reported by Boons and Popik.^81^ In their study, the authors exploited the photoconversion of 3-(hydroxymethyl)-2-naphthols (**55**) into *o*-quinone methides (*o*-NQMs, **56**) (Scheme 14) for the conjugation of peptides and proteins.

**Scheme 14:**
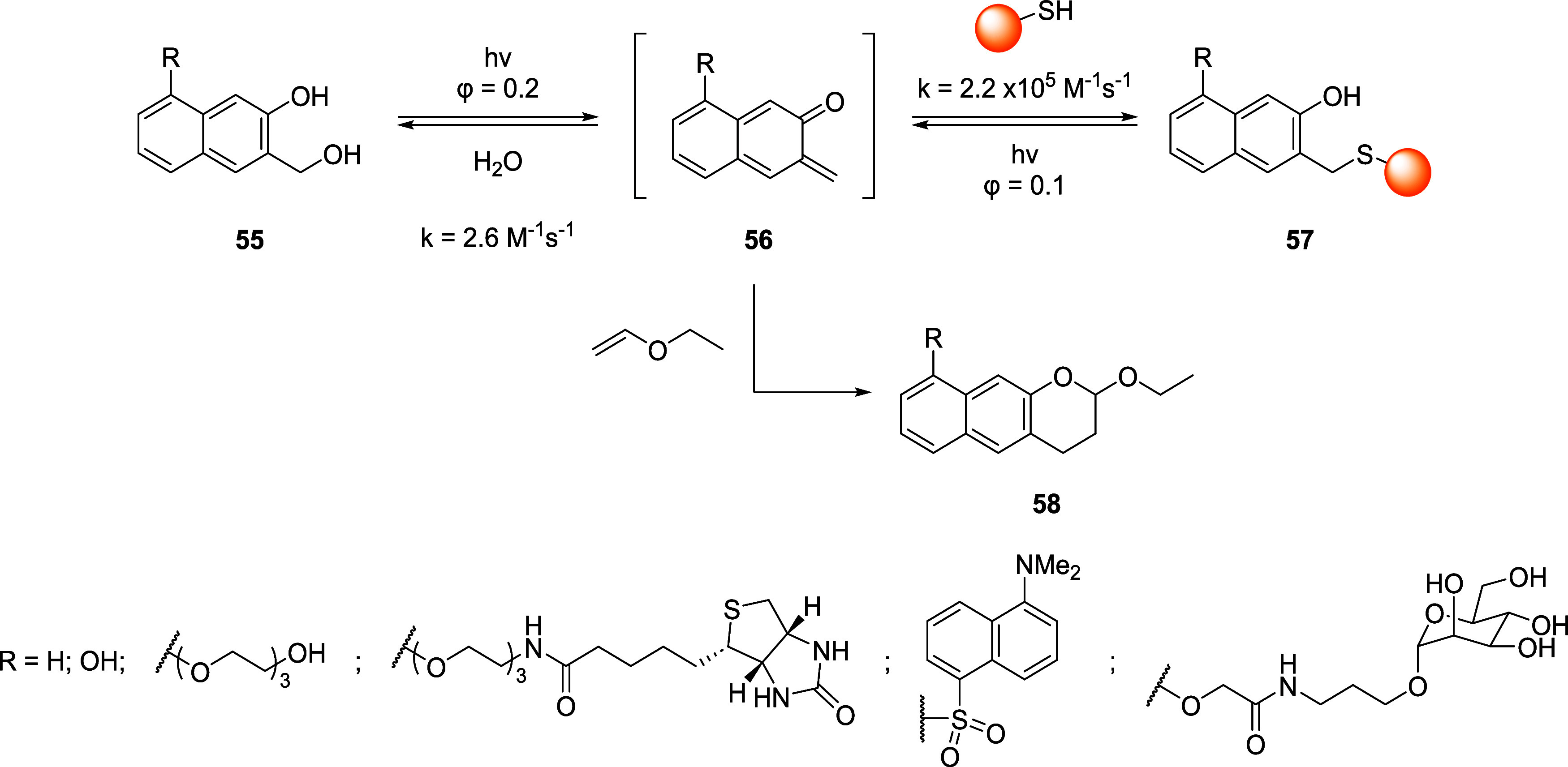
Labeling Strategy for the Bioconjugation of Peptides and Proteins with *o*-NQMs

The advantage of the employment of such a linker lies in its reversibility for peptide regeneration and its high specificity for thiols. In fact, in a previous work, Popik demonstrated the selectivity of *o*-NQMs toward thiol in the presence of other nucleophiles, such as water and azide ions.^82^ These compounds feature characteristic UV absorption bands at around 270 and 320 nm, the latter extending past 360 nm, making it suitable for stimulation with a 350 nm fluorescence lamp to promote photochemical-assisted dehydration. The selectivity of *o*-NMQ toward cysteine was tested with peptides that contain other nucleophilic amino acids, such as Lys, Tyr, His, Asp, and Ser. The authors observed by HPLC and MS/MS measurements that the labeling with TEG-*o*-NMQ occurred only on the cysteine residue, whereas no reaction was observed on the other amino acids when cysteine was replaced with methionine or oxidized to a disulfide bridge. The authors also optimized the reaction between peptide and *o*-NMQ with the optimal ratio of 1:4 to achieve a quantitative yield after 2 min of irradiation at 300 nm, with higher ratios leading to a decrease in yields up to 20% for a 1:1 ratio between the two reactants. On the other hand, peptide photoregeneration can be achieved at any concentration of adduct when vinyl ethyl ether is used as *o*-NMQ trapping agent, reacting via Diels–Alder cycloaddition and giving the photostable benzochroman (**58**) as product. Given the encouraging results obtained with peptides, the authors explored the labeling of the solvent-exposed cysteine residue (Cys34) of BSA, both in the dark and under irradiation at physiological pH. In all cases, the reaction was successful only under irradiation and in the presence of a free cysteine residue, whereas no reaction was observed either in the dark or in the case of prior modifications on the target amino acid. Irradiation at 350 nm for 2 min efficiently restored BSA, confirming the reversibility of the reaction even in the case of protein labeling. For more details, we kindly invite the reader to look up ref 81.

### 2.3 Oxanorbornadienes (ODNs)

The last scaffold that will be described in this section belongs to the norbornadiene family, more specifically, the alkyl oxanorbornadiene-2,3-dicarboxylates (labeled in this review as “oxanorbornadienes”, ONDs, for simplicity). Even though they were isolated for the first time by Otto Diels and Kurt Alder in 1931,^83^ OND experienced a golden age as linkers in bioconjugation, which started in 2009 with research carried out by the group of M. G. Finn. As first described by Diels and Alder, these compounds are obtained by [4 + 2] cycloaddition, *i.e.*, Diels–Alder reaction, between furan and electron-deficient alkynes, such as alkyl acetylenedicarboxylates. It was also reported that, in certain conditions (*e.g.*, high temperatures) that these compounds can undergo cycloreversion, *i.e.*, retro Diels–Alder (rDA) reaction, to give the parent compounds.^84,85^ The first cycloreversion process at mild conditions was described in 2000 by Deloisy, who observed rDA reaction of ONDs upon reaction with thiophenol, obtaining furan derivatives and two sulfur-containing diastereomeric olefins (*Z*/*E* = 75/25).^86^ With this approach in mind, Finn’s group extensively explored the chemistry of ONDs over the last 20 years, focusing on their application in several fields, from bioconjugation chemistry to synthetic chemistry. In fact, the OND scaffold contains an electron-deficient carbon–carbon double bond that is highly reactive toward thiol nucleophilic addition. This reactivity is further enhanced by ring strain and the electronic nature of the substituents on the OND core. The first attempt of bioconjugation by means of dialkyl oxanorbornadienes-2,3-dicarboxylate was carried out with fluorogenic derivatives, obtained by introducing dansyl group (Dn) in the molecule.^87^ The OND derivatives reported in this study were synthesized by the Diels–Alder reaction between *N*-dansylfurfurylamine or *N*-dansyl­(5-methylfurfuryl)­amine and dialkyl acetylenedicarboxylates. In this way, the author obtained fluorogenic ONDs (Scheme 15) with both symmetric maleate moieties featuring methyl (**59–61**), ethyl (**62**), or propargyl esters (**63** and **64**) and asymmetric maleate moieties featuring a combination of esters and amides (**65** and **66**) or trifluoromethyl and esters (**67**). Like fluorescent dyes attached to maleimides, dansyl-substituted ONDs undergo quenching of the chromophore. Thus, disruption of the conjugation of the maleate moiety after Michael addition restores the fluorogenic properties of the dansyl group.

**Scheme 15:**
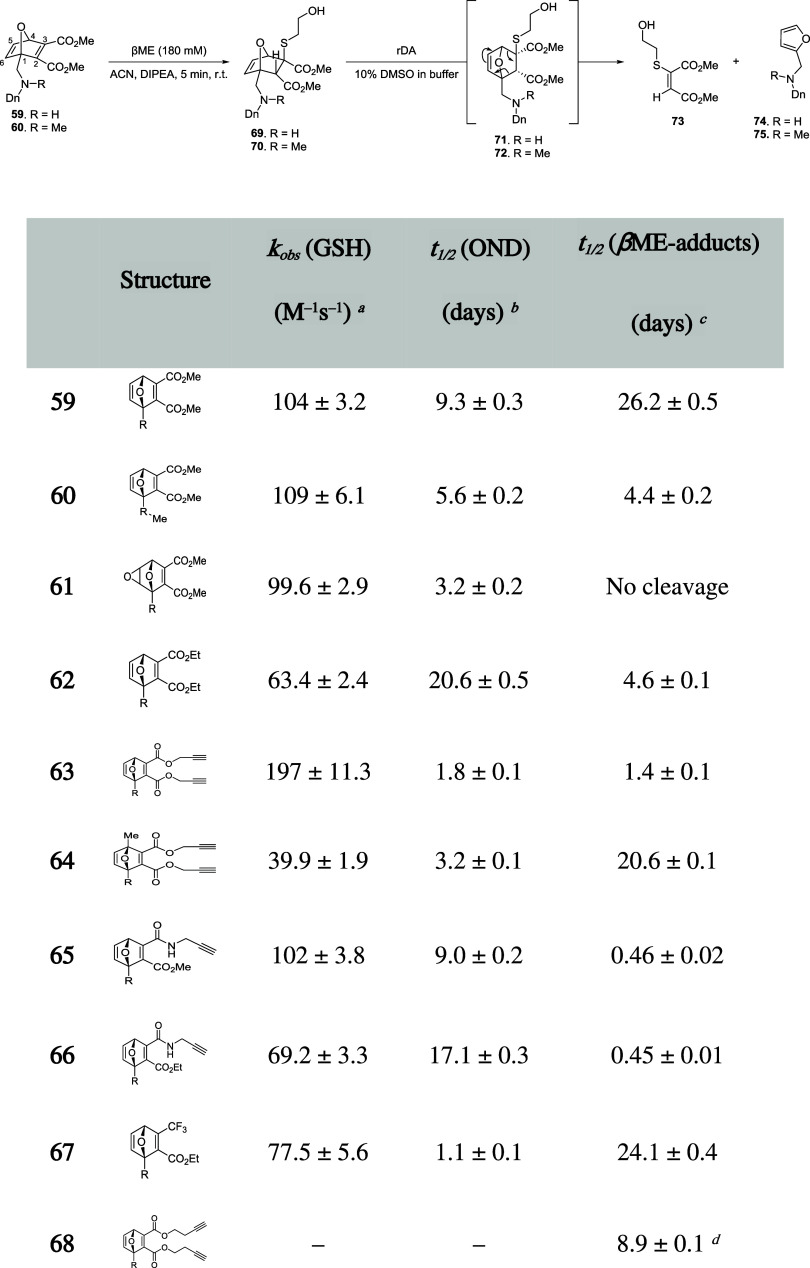
General Synthethic Strategy for Thiol Conjugation by OND Electrophiles and Release by the rDA Reaction

a Observed reaction rate of thiol-Michael addition with GSH, calculated by means of fluorescence analyses.^87^

b Calculated from the first-order rate constants.^87^

c Calculated by periodic HPLC analysis, following the disappearance of the βME-adduct peak with time.^87^

d Calculated by ^1^H NMR in CDCl_3_ at 25 °C.^88^

The reactivity of OND reagents was tested against a discrete number of nucleophilic amino acids, resulting in high selectivity for thiols—yielding an intense increase of fluorescence—and slightly reactive toward lysine and histidine, contributing to a small (<2%) increase of fluorogenic properties of the dansyl group. The reactivity of OND electrophiles was also tested against glutathione at pH 7 and the reaction was followed by the increase of fluorescence at 550 nm, resulting in a second-order kinetic, as expected from Michael addition, with rate constants in the range of 40–200 M^–1^ s^–1^. In this way, the authors could also have an impact on OND stability in aqueous media, whereas the addition of βME allowed us to evaluate cycloreversion rates. As shown in Scheme 15, all OND electrophiles exhibit a good reactivity profile toward GSH, with **63** being the most reactive of the series, whereas the reactivity is inhibited in compound **64** because of the introduction of the bridgehead methyl. Half-life values in aqueous media of these compounds are extremely low—especially for the less reactive **64**—due the occurrence of noncanonical side reactions (ester hydrolysis and water addition are the most common pathways).^89^ The most interesting results were obtained with compounds **59**, **62**, **65**, and **66**, which feature high reactivity and high stability toward aqueous deactivation. Moreover, the regioselectivity of the reaction was well-defined, leading to an *exo*-*syn* addition with the thiol group attached on the less hindered position (C3)—as single diastereoisomer—when no substituent is present in the adjacent bridgehead position (**71** and **72** in Scheme 15). Considering the adduct half-life values referred to rDA reaction, epoxidation of double bond in position 5 (**61**) led to an extremely stable adduct due to the impossibility of cycloreversion to afford a furan moiety. Moreover, conducing this reaction with *N*-methylated derivative of **59**, *i.e.*, **60**, gave 6-fold higher rDA rates due to the absence of hydrogen bonding between the dansyl secondary amino group and the maleate moiety (**72** in Scheme 15). For thiol-adducts derived from compounds **65** and **66**, the rDA rates are identical despite the differences in the thiol reactivities of these two OND electrophiles. This reactivity could be explained by the presence of the common intermediate **78**, which is produced by intramolecular cyclization of nonisolated compounds **76** and **77** promoted by the release of conformational strain upon thiol addition. Compound **78** readily undergoes cycloreversion, affording thiol-maleimide **79** and furan **74** (Scheme 16). **79** is a key intermediate as thiol-maleimides are prone to thiol exchange reaction, as shown in the next section, allowing protein conjugation and its recovery in the presence of an additional thiol.

**Scheme 16:**
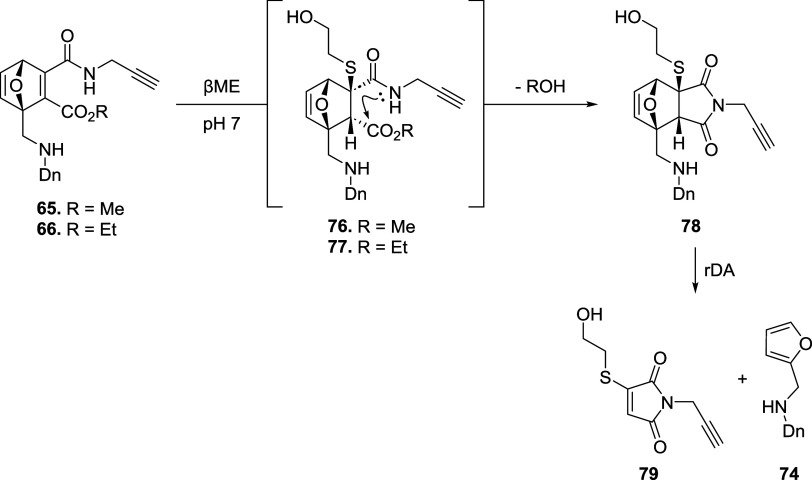
Intramolecular Cyclization of ONDs **65** and **66** to Form the Common Succinimidyl Intermediate **78**

The most promising OND electrophiles, *i.e.*, **59**, **65**, and **66**, were then employed for bioconjugation experiments with a peptide or BSA. Despite the presence of different nucleophilic amino acids—arginine, glutamic acid, lysine, cysteine, and threonine—the peptide was labeled within 1 min by the above-mentioned ONDs as confirmed by the detection of a strong fluorescence signal. Thiol selectivity was assessed by pretreating the peptide with *N*-ethylmaleimide, leading to no reaction with the OND electrophiles. BSA conjugation was carried out with ONDs **59**, **65** and **80**, a fluorescent derivative of **66**, with complete labeling of Cys34 in 2 h, as highlighted by dansyl fluorescence following denaturing gel electrophoresis (Scheme 17). BSA adduct of **65** was less stable than the one obtained with **59**, as expected by considering the tendency of **65** to undergo rDA reaction. The most interesting results were obtained with compounds **61** and **80**, which allowed to perform permanent labeling of BSA (Scheme 17).

**Scheme 17:**
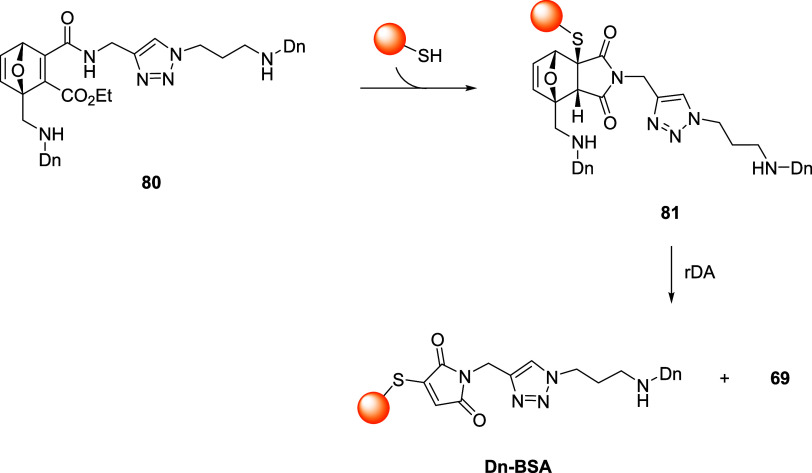
Synthetic Strategy for the Permanent Fluorescent Labeling of BSA through OND Degradation by the rDA Reaction

To deeply understand the criteria that rule stability of OND electrophiles, the same group explored new combinations of substitutions between position 1 and the electrophilic maleate moiety.^89^ Even in this case, the authors confirmed the regioselectivity of the reaction between symmetrical OND electrophiles and βME, obtaining the 3-*exo*-*syn* adduct as the sole product in near quantitative yield with ONDs with a single bridgehead substituent, as highlighted by ^1^H NMR. On the other hand, asymmetrical 1,4-disubstituted ONDs give a mixture of 2-*exo*-*syn* and 3-*exo*-*syn* adducts characterized by high cycloreversion rates, rapidly decomposing into the corresponding 2,5-disubstituted furan and thiomaleate. Half-life values were evaluated by NMR spectroscopy for ONDs derived from furfuryl amines, sulfonamides, amides, ureas, carbamates, and alcohols. The authors also explored the reaction between ONDs and other nucleophiles. Small phosphines, such as trimethylphosphine and tris­(carboxyethyl)­phosphine (TCEP), yielded labile adducts, whereas bulkier phosphines, like triphenylphosphine, and tertiary amines were found to be unreactive. On the other hand, nontertiary amines yielded diastereomeric adducts with surprisingly high half-lives, except for the 1,4-disubstituted OND **78**. Quenching the reaction with trifluoroacetic acid (TFA) destabilized the adducts, drastically reducing half-life values of both *syn* and *anti* diasteroisomers (Scheme 18).

**Scheme 18:**
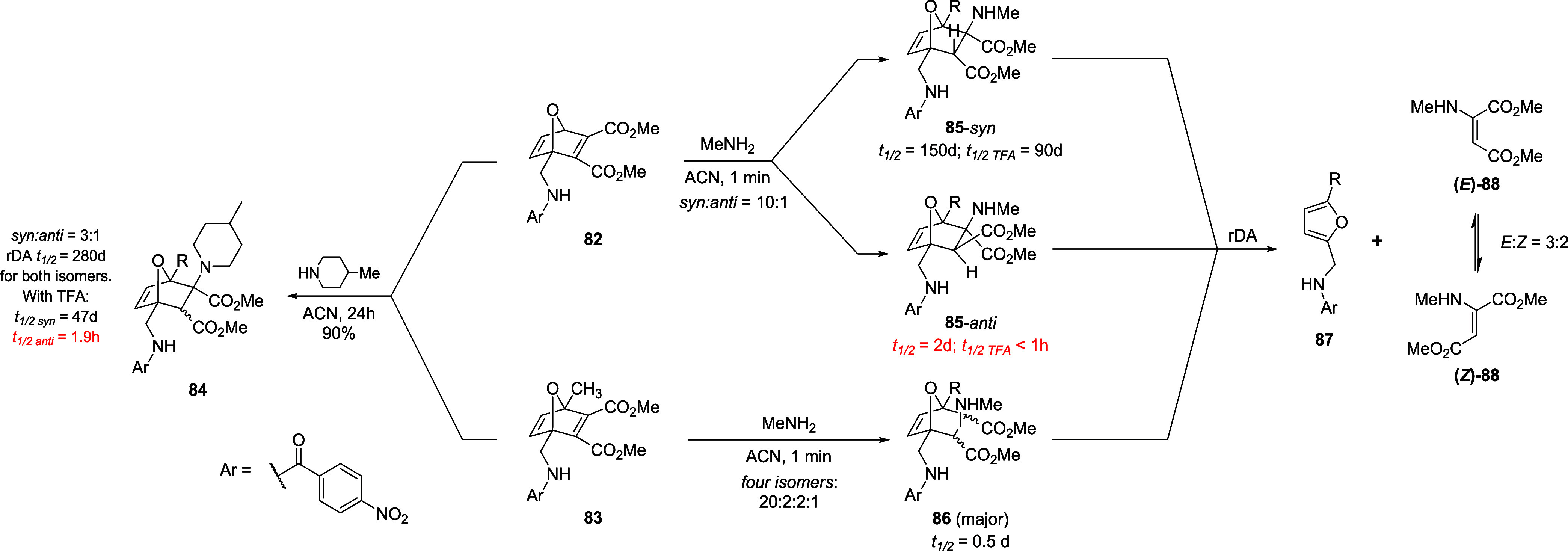
Synthesis and Cycloreversion of Amine Adducts from ONDs **82** and **83**

The faster cycloreversion of the *anti* adduct is attributed to an intramolecular hydrogen bond between the protonated amine and the proximal ester group which promotes a coplanar alignment, triggering rDA reaction (Figure 6A). In general, amine adducts are more stable than thiol adducts due to a less tendency of the nitrogen atom to stabilize the transition state by n→σ* donation (Figure 6B). In fact, this type of interaction is favored in the case of aromatic thiol-adducts which undergo rapid cycloreversion; and it is less favorable with trifluoromethyl substituents, accounting for the high stability of OND adducts featuring this functionality. Moreover, Additional stabilization comes from a hydrogen bond interaction between the furfuryl-derived NH and the ester in C2, as mentioned in the dansyl series (Figure 6C). In the case of *para*-benzamide substituents, the authors observed a decreased stability along with decreased acidity of the amide, as the electron-donating ability of the *para* substituent increases.

**Figure 6:**
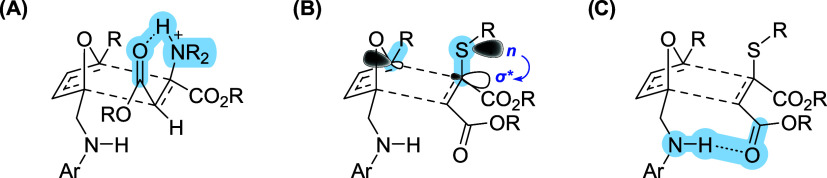
Illustration of the interactions that influence the stability of thiol or amide OND adducts.

In 2021, De Pascalis et al. synthesized several OND derivatives to evaluate by ^1^H NMR the effect of different substituents on the rate of cycloreversion of the thiol adducts (Figure 7).^90^

**Figure 7:**
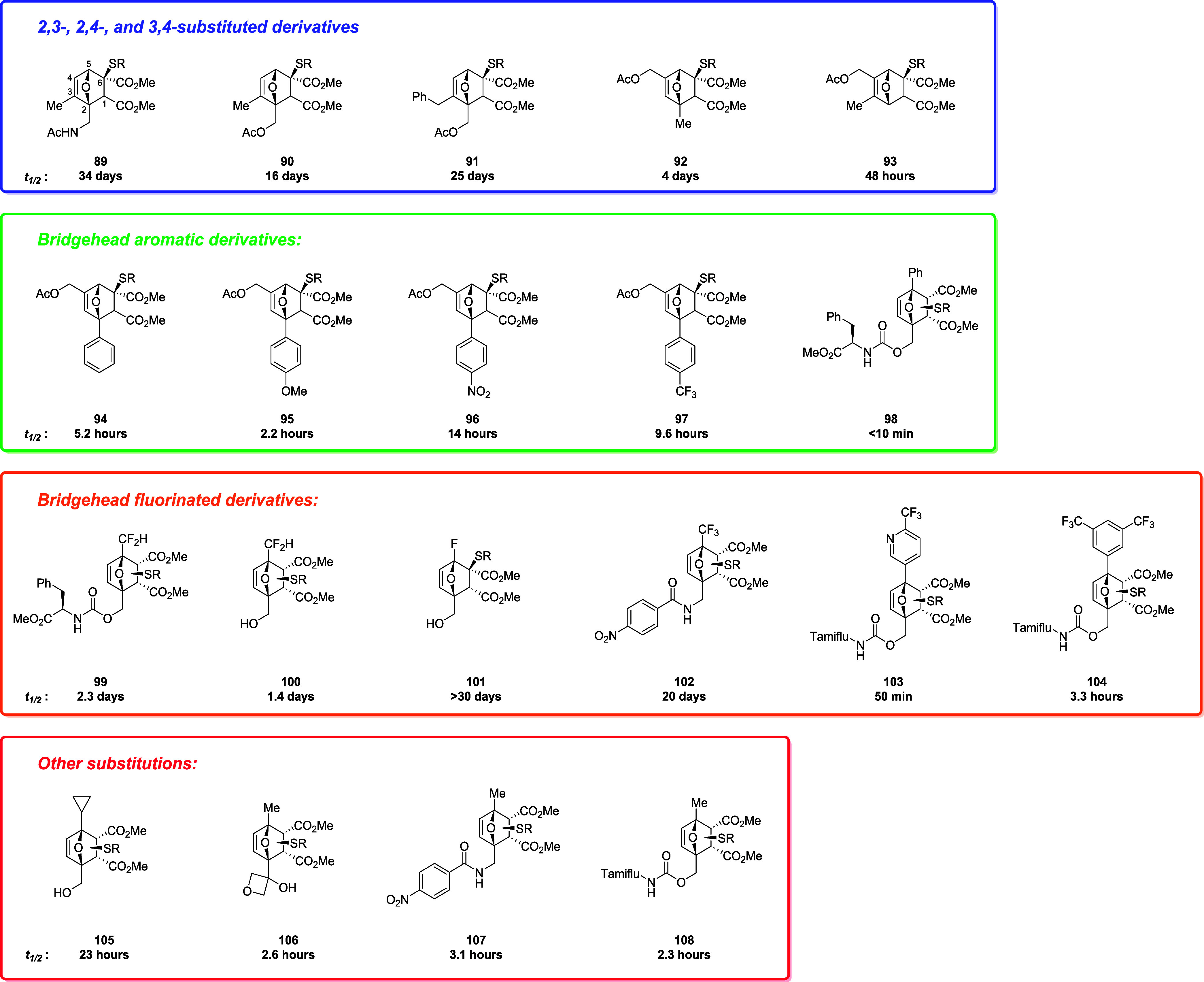
Structures and half-life values of compounds **89–108**.

Among the different patterns of substitutions, 2,4- and 3,4-disubstituted OND adducts showed short half-life values in contrast with 2,3-disubstituted derivatives (**92** and **93** vs **89–91**) which feature half-lives ranging from 16 to 34 days. Moreover, bridgehead aromatic substitution (**94–98**) accelerates the rDA reaction thanks to electronic effects, as confirmed by Hammett *σ^+^* values. Bridgehead substitution with fluorinated substituents (**99–104**) was also explored, highlighting a stabilization of the thiol adduct with the trifluoromethyl derivative (**102**) being more stable than the difluoromethyl ones (**94** and **95**). Among these, aromatic fluorinated compounds (**103** and **104**) showed indeed higher stability compared to similar OND derivative **98**. In the end, the cyclopropyl substituent in the bridgehead position increased the half-life of derivative **105** compared to 5-methyl substitution in compounds **106–108**. Calculation of Hirshfeld charges provided an explanation for the observed half-lives, considering that stabilization of positive charges in the transition correlates with higher rDA rates. In fact, the most relevant examples supporting this observation are aromatic and fluorine substitutions: the presence of electron-donating groups like in compound **95** decreases thiol adduct half-life, in contrast with trifluoromethyl substituent in **96** and more evidently with **97**, which feature electro-withdrawing groups, and thus longer thiol-adducts half-lives. On the other hand, electron-withdrawing fluorinated substituents stabilize the thiol-adduct, like in compounds **99**, **100**, **102**, and **101**, where the fluorine atom is directly attached to the bridgehead carbon, accounting for the highest stabilization effect observed in this work. Aromatic derivatives showed lower stability than the aliphatic ones also because of additional stabilization of the conjugated furan system, which is mostly restored in the transition state, favoring the rDA reaction. The authors then evaluated the reactivity of **68** toward *N*-acetylcysteine, tri­(glycine), *N*-benzoyl histidine, and 6-aminocaproate, as model compounds of cysteine, *N*-terminal glycine, histidine, and lysine, respectively.^89^ As expected, the reaction between **68** and *N*-acetylcysteine exhibited the fastest reaction rate with a second-order kinetic constant between three and 5 orders of magnitude greater than those observed for the other model compounds. Protein labeling was then performed with 4 different fluorogenic dansyl-functionalized ONDs toward BSA (thiol content of 11%) and reduced BSA (rBSA) (thiol content of 95%)—reduced with DTT—and monitored by measuring the increase of fluorescence upon Michael addition. All tested fluorogenic ONDs exhibited similar reaction profiles, each of them constituted by two different contributions: the first is an initial burst of fluorescence linked to thiol addition, as measured by the Ellman assay; the second shows a slower, nearly linear increase in fluorescence related to amine addition, similar to OND reaction with unreduced BSA. By monitoring the release of furan moiety by means of fluorescence spectroscopy, the authors found that both bridgehead substituents and methylation of the sulfonamide produce short-living adducts, with amine adducts being more stable than thiol adducts, as seen in the preliminary studies with primary amines. These results led the group of Finn to employ ONDs for the bioconjugation of rat serum albumin (RSA) *ex vivo*.^91^ For this purpose, electrophiles with OND scaffolds similar to those of **59**, **61**, and **67** were employed. These compounds were functionalized with Gd-DOTA (a gadolinium-based MRI contrast agent) to mimic the release of a hydrophilic cargo. The authors highlighted good labeling values for the dimethyl ester **59** and for the fluorinated derivative **67**, whereas the epoxidized derivative **61** was unreactive toward RSA and exhibited the same elimination rate as the furan parent compound, probably due to rapid hydrolysis and excretion from the organism. It is worth mentioning that the authors observed a high reactivity of the fluorinated derivative not only toward Cys34, but also toward several amine residues in both thiol-capped and untreated RSA. The group of Finn explored different applications of OND electrophiles, like the release of pharmaceutically relevant cargos,^92^ the design of modular degradable hydrogels,^93^ and the protection of amines.^94^ For the first application, several ester–amide OND (EA-ONDs) electrophiles were synthesized as models for the evaluation of the release of pharmaceutically relevant alcohols (Figure 8).^92^

**Figure 8:**
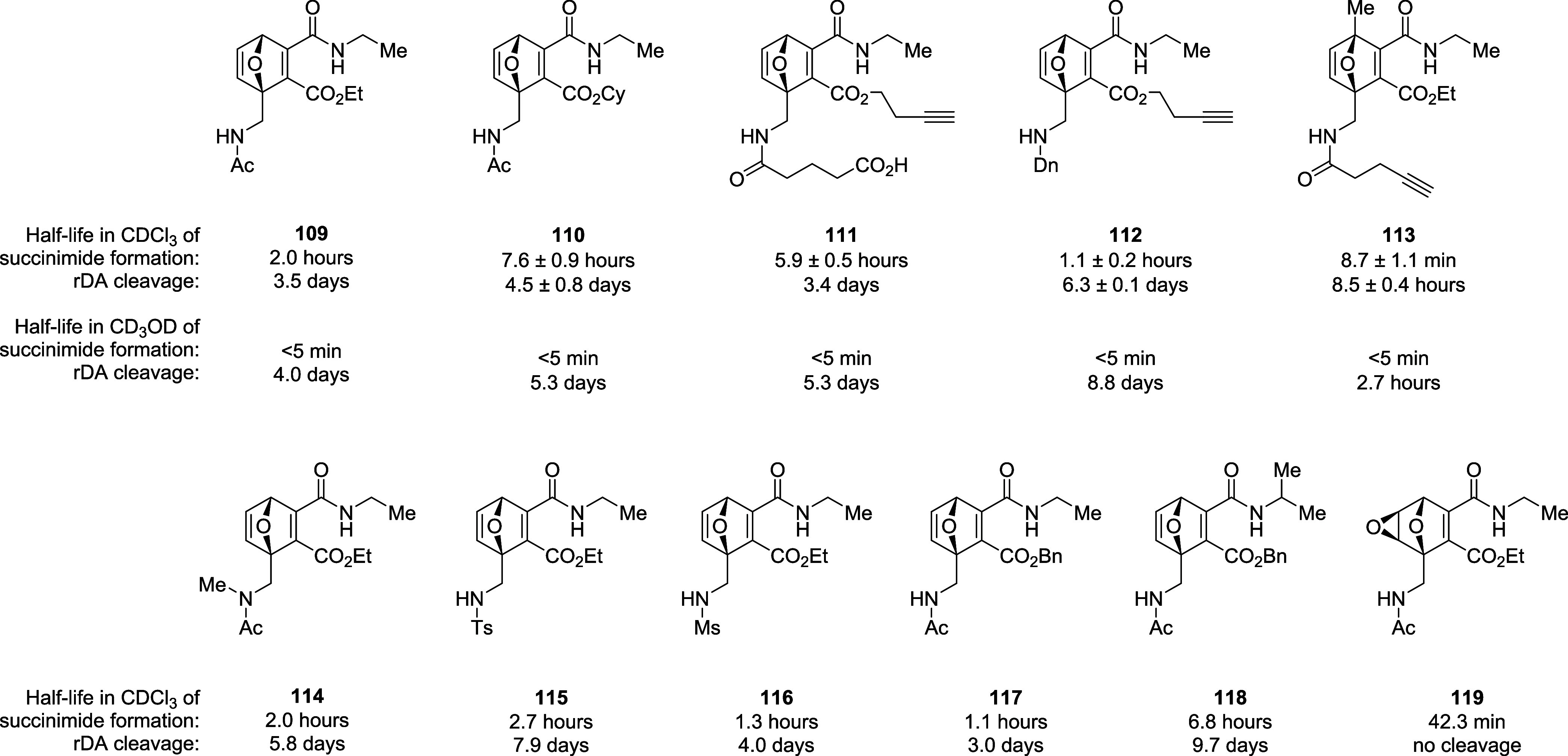
Structures and half-life values of EA-OND electrophiles **109–119**.

Like EA-ONDs **65**, **66**, and **80**, these compounds undergo intramolecular cyclization upon reaction with βME to afford the corresponding succinimide, releasing the alcohol as a byproduct (see Schemes 16 and 17). The authors investigated the releasing kinetics of these adducts by means of ^1^H NMR analysis in chloroform-*d* and methanol-*d*_4_, observing a strong solvent effect: methanol-*d*_4_ drastically accelerates succinimide formation with a negligible effect on rDA reaction (Figure 8). Moreover, the nature of the substituent attached to the furfuryl amine nitrogen does not affect the rate of cyclization, but *N*-methylation decelerates succinimide formation due to the lack of intramolecular hydrogen bonding (**109** vs **114**). Increasing the size of the amide chain slows down the ring closure reaction by a factor of 3, whereas the nature of the ejected alcohol has a higher impact on the reaction rate, with primary alcohol being eliminated more easily than bulkier alcohols (**109**, **112–119** vs **110**). Since EA-ONDs are prepared from an asymmetric alkyne, two possible regioisomers (**120–122** and **124–126**) are formed upon Diels–Alder reaction, which show different reactivities: the 3-amide regioisomers (**120–122**) give faster cyclization and rDA than the 4-amide ones (**124–126**) (Scheme 19). This reactivity was confirmed using cholesterol as a model alcohol, observing faster succinimide formation and cycloreversion in CDCl_3_ for the 3-amide regioisomer (180-fold and 195-fold, respectively). As expected, ^1^H NMR analysis in methanol-*d*_4_ highlighted fast cholesterol release even for the 4-amide regioisomer, suggesting that this process might be fast even in the context of the biological environment for bioconjugation.

**Scheme 19:**
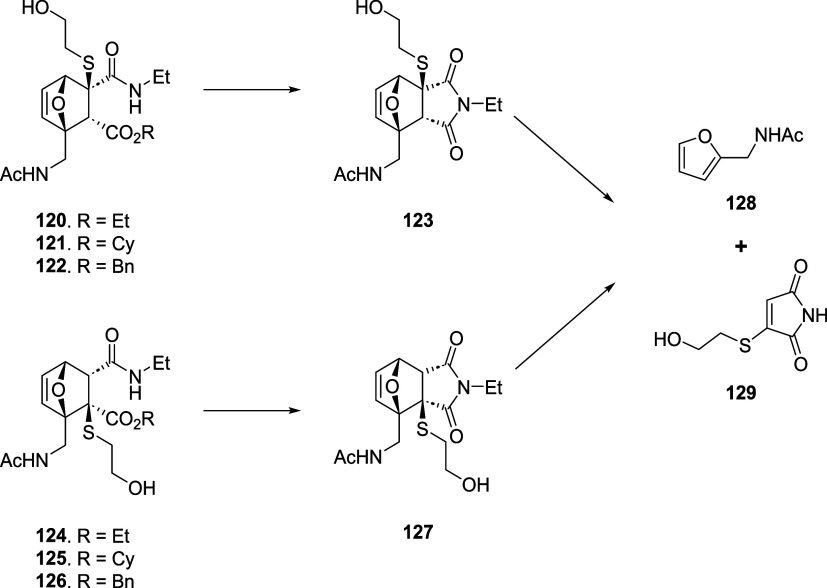
Structure and Cycloreversion of 3-Amide and 4-Amide βME-OND Adducts (**120–122** and **124–126**, Respectively)

The group of Finn finally explored the chemistry of structurally related compounds, *i.e.*, azanorbornadienes (ZNDs).^95^ Like ONDs that are synthesized via the Diels–Alder reaction starting from furan, ZNDs represent the pyrrole-derived counterpart, sharing with ONDs the reactivity with thiols and the tendency to undergo cycloreversion. However, although ZNDs showed lower reactivity toward thiol addition if compared to the structurally related ONDs, the advantage of their employment in bioconjugation is the lack of potential furan-associated metabolic toxic effects caused by P450 processes.^96^ To evaluate rDA rates, the authors synthesized 6 electrophiles (**130–135**) and monitored βME addition and cycloreversion by means of ^1^H NMR (Figure 9).

**Figure 9:**
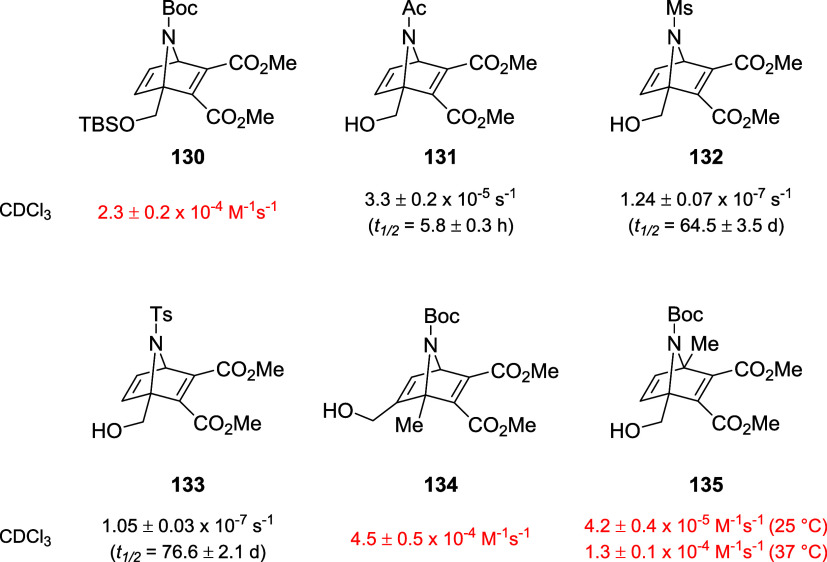
Structure of ZND electrophiles and rDA half-life values of the βME-adducts.

Half-life values immediately suggest that rDA reaction occurs at slower rates compared to OND electrophiles, with *N*-mesyl and *N*-tosyl derivatives being far more stable than the *N*-acyl derivative (**132** and **133** vs **131**). This behavior might be explained by taking into account the hybridization of the involved N atom, which is a *sp^2^*-*N* center in **131**, which might have a larger contribution to the restoration of pyrrole aromaticity in the rDA process. For compounds **130**, **134**, and **135** half-lives of thiol adducts could not be obtained since the corresponding pyrroles and maleates were immediately identified in the NMR spectra, suggesting that the rate-determining step is thiol addition due to steric hindrance of the Boc protecting group. Two mixtures of two ZNDs (**131** and **132**) and their corresponding OND derivatives were employed for a competition experiment, in which furan or pyrrole formation upon cycloreversion was monitored by ^1^H NMR. In the case of **131** and the corresponding OND derivative, both furan and pyrrole were detected after 33 days after treatment with 0.5 equiv of βME. On the other hand, in the case of **135**, furan was the sole product detected after consumption of βME after 48 h, confirming in both cases the higher reactivity of ONDs.

## 3 Addition–Elimination Reaction

Bromomaleimides, indenedione-based linkers, and bromopyridazinediones form a class of thiol-selective reagents that have been documented for reversible protein conjugation via an addition–elimination mechanism. Specifically, a thiol adds to an electron-deficient carbon–carbon double bond and, via the E1cb mechanism, expels a leaving group (LG) located at the β position, yielding a thioether-linked adduct. The resulting linkage is stable under physiological conditions and can be cleaved by a thiol exchange. Specifically, an incoming free thiol can attack the thioether bond and displace the original one, regenerating the electrophilic alkene and liberating the first thiol (Scheme 20).

**Scheme 20:**
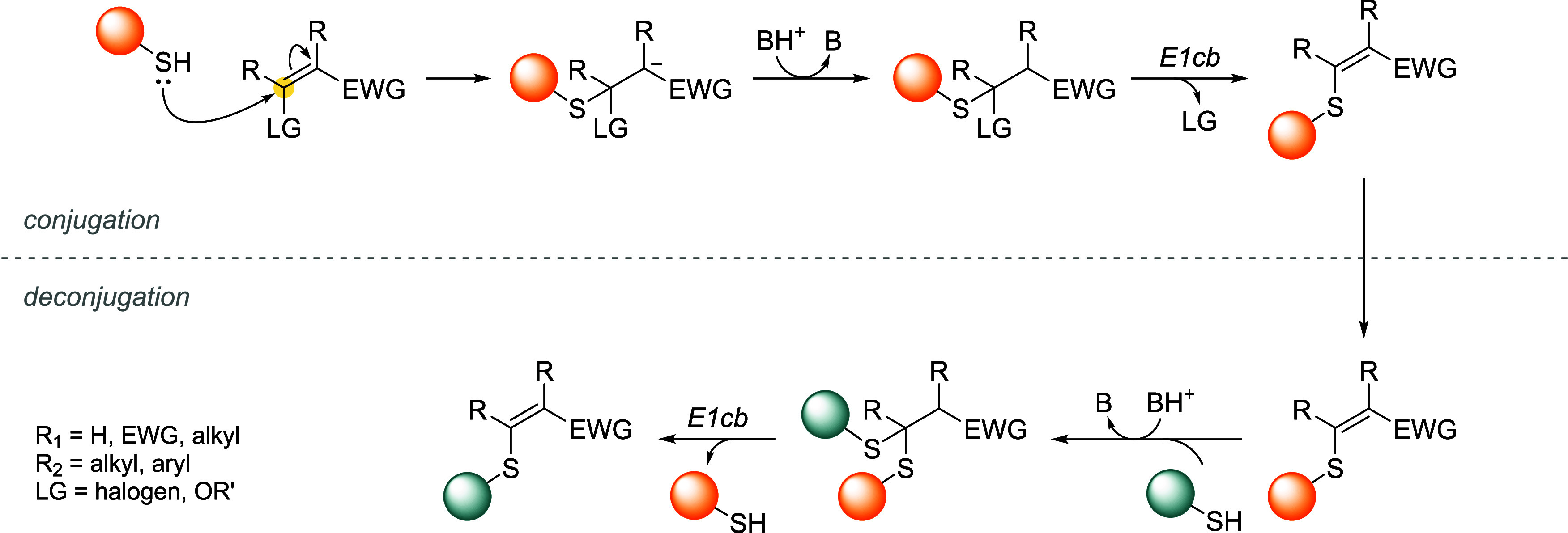
Addition–Elimination Reaction General Mechanism^a^

a Bioconjugation sites are marked with a yellow dot.

This process preserves the thiol-adduct in the absence of competing thiols but can be detached or swapped in the presence of excess competing thiols, allowing the controlled release or exchange of functional payloads. Bromomaleimides were the first to exemplify this strategy, achieving a high cysteine selectivity. The following sections detail the mechanism and applications of each reported linker type (Figure 10), focusing on how their structural features influence the reversibility of bioconjugation.^28,97,98^

**Figure 10:**
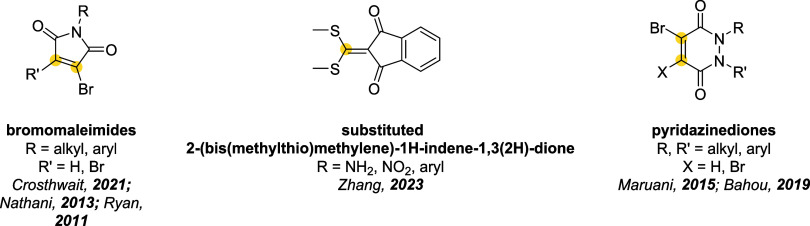
Different classes of thiol-selective scaffolds documented for reversible conjugation via an addition–elimination mechanism. Bioconjugation sites are marked with a yellow dot.

### 3.1 Bromomaleimides

Bromomaleimides are maleimide derivatives bearing a bromine substituent on the double bond (Figure 11). Thiol addition proceeds through a Michael-type addition on the β-carbon of the bromomaleimide and extrusion of bromide as the leaving group. Crucially, this conjugation is reversible: the thiomaleimide lacks an acidic α-proton (having formed *via* bromide elimination), so it does not undergo spontaneous retro-Michael cleavage. Instead, reversibility can be achieved through thiol-exchange. Furthermore, the hydrolytic stability of the adduct has been tested at pH 8 (37 °C) showing an increased resistance if compared to the maleimide derivatives (Figure 11).

**Figure 11:**
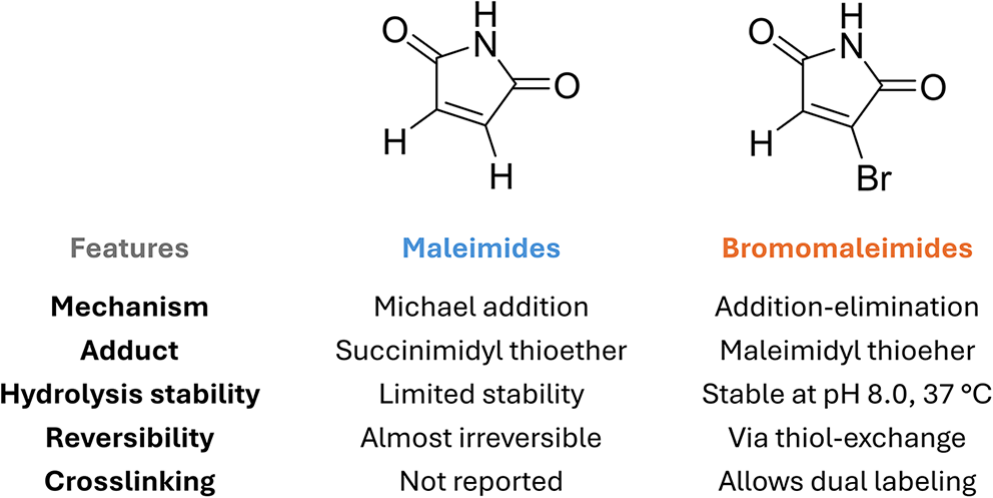
Comparison between the features of maleimide and bromomaleimide scaffolds in bioconjugation chemistry.

In 2010, Smith and co-workers demonstrated a reversible cysteine modification and disulfide bridging on proteins using bromomaleimides.^99^ Specifically, single-cysteine protein Grb2-SH2 was functionalized with a *N*-methylbromomaleimide (MBM) in a phosphate buffer with TCEP at pH 8 (mild conditions), yielding a quantitative bioconjugation in 1 h at 0 °C. A similar procedure was used with dibromomaleimide, that has been reacted with a second thiol (*e.g.*, GSH, thioglucose) to give divalent conjugates that cleanly released the native protein with excess of thiol (*e.g.*, 100 equiv of dithiothreitol-DTT or βME) whereas TCEP was ineffective, consistent with the thiol exchange mechanism required for the cleavage process. In one another relevant example, Nathani et al. confirmed that a protein–maleimide conjugate can be quantitatively cleaved by treatment with excess glutathione or βME, regenerating the free cysteine.^98^ In their work, they selectively conjugated the biotinylated bromomaleimide to a target cysteine residue on a protein present on a streptavidin-coated surface. The covalent adduct can be cleaved in the presence of DTT, thereby releasing the unmodified functional protein without resorting to harsh or denaturing conditions (Scheme 21).

**Scheme 21:**
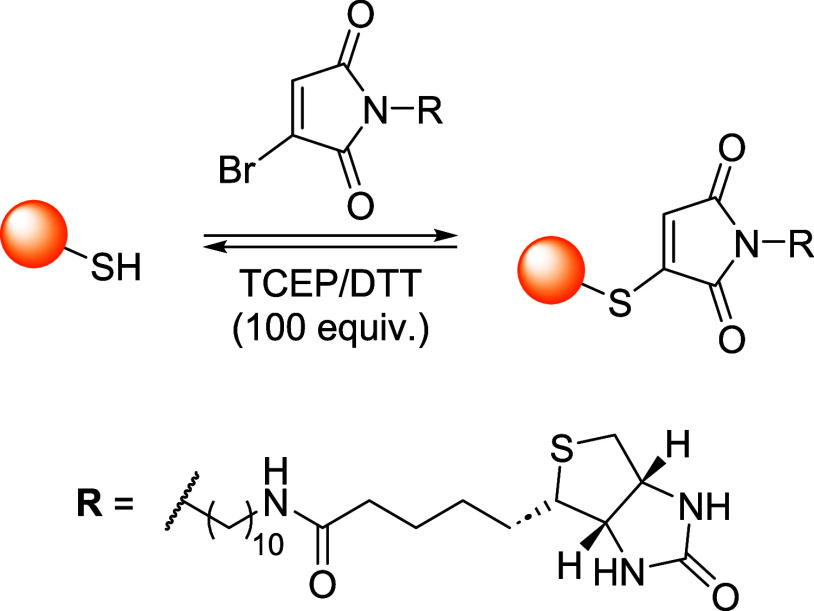
Conjugation of Biotinylated Bromomaleimide with a Cysteine Residue of a Protein

Thus, maleimide conjugates are thermodynamically stable and kinetically labile in the presence of competing thiols, allowing them to serve as versatile handles for cysteine labeling. Another use of this selective linker was reported by Lindsey-Crosthwait et al., who developed a reversible peptide-stapling strategy based on dibromomaleimide cross-linkers.^28^ Their work consisted of forcing peptides into α-helical conformations to inhibit protein–protein interactions by chemically linking two thiol-containing residues (*e.g.*, cysteine or homocysteine) using dibromomaleimide; in this way, they investigated how different amino acid configurations could influence the stapling of the peptides. Importantly, as in the previous case, the dibromomaleimide-based staples could be reversed in the presence of DTT, restoring the original peptide.^99^

### 3.2 Indenediones

Indenedione-based linkers (exemplified by Indane-1,3-dione derivatives) show a similar Michael-type addition mechanism, but with a relevant difference: the electrophilic alkene is conjugated to a cyclic 1,3-diketone (indenedione) scaffold that can form an exceptionally stabilized enolate upon thiol addition by the extended conjugation of the 1,3-diketone system.^100^ Consequently, the cysteine–indenedione conjugate is persistently locked in place under physiological conditions with no significant dissociation. For this reason, indenedione-type linkers and the reversibility of their conjugates do not imply spontaneous dissociation but instead a nucleophile-induced exchange, making their cleavage more controllable. This mechanistic distinction was effectively utilized in a recent study by Zhang et al., who developed a series of probes, namely **IDA** (**136**), **IDA-1** (**137**), and **IDA-2** (**138**), structurally analogous to indenedione electrophiles for the selective labeling of vicinal dithiol-containing proteins (VDPs) in live cells (Figure 12).^100^

**Figure 12:**
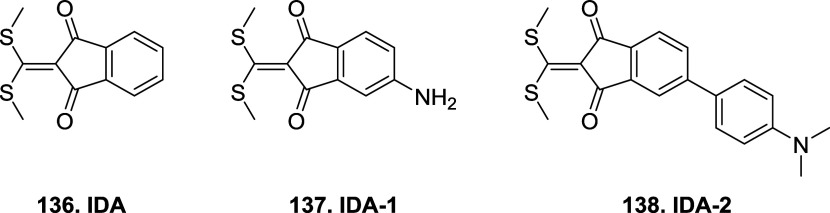
Indenedione probes for the selective labeling of vicinal dithiol-containing proteins.

These probes react chemoselectively with pairs of cysteines in proteins (in the case study, thioredoxin and glutaredoxin), forming stable conjugates that fluoresce upon binding. Importantly, the conjugation was shown to be fully reversible upon treatment with reducing agents such as DTT, restoring the native protein thiols (Scheme 22).

**Scheme 22:**
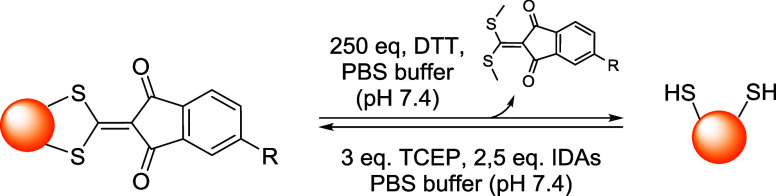
Reversible Conjugation of the IDA Series Triggered by a Reducing Agent

Among these indenedione-type probes, **IDA-1** and **IDA-2** exhibited significantly reduced reactivity compared to **IDA** toward primary amines, such as ethylenediamine (EDA), as monitored through UV–Vis spectra and thermodynamic analysis using an isothermal titration microcalorimeter. The binding constant of **IDA**-**1** with EDA was *K* = (1.84 ± 0.115) × 10^3^ M^–1^, lower than the value for **IDA** (*K* = (3.49 ± 0.365) × 10^3^ M^–1^). Meanwhile, the binding constant of **IDA**-**1** with ethylenedithiol (EDT) was calculated to be (392 ± 2.41) × 10^5^ M^–1^, much higher than that of **IDA**-**1** with EDA as above. Importantly, varying the R substituent affects the reversibility of thiol conjugation: sterically hindered amines, such as in **IDA**-**2**, destabilize the adduct and favor retro-substitution, whereas smaller amines (such as in **IDA-1**) enhance adduct stability.

### 3.3 Bromopyridazinediones

Another relevant example of addition–elimination reactions consists of the use of bromopyridazinediones (BrPDs) as bioconjugation linkers. Their versatile nature, depending on the mono/disubstitution with bromine atoms, allows them to generate different types of bioconjugates reflecting different applications of the same scaffold. Mechanistically, the first thiolate attack on BrPD yields a monothioether intermediate (with one bromide remaining on the pyridazinedione scaffold); in the case of dibromopyridazinediones (diBrPDs) and in the presence of a supplementary thiol (either coming from an excess of the first or from a different molecular source), the latter one then attacks the remaining C–Br, completing the bridge (Scheme 23).

**Scheme 23:**
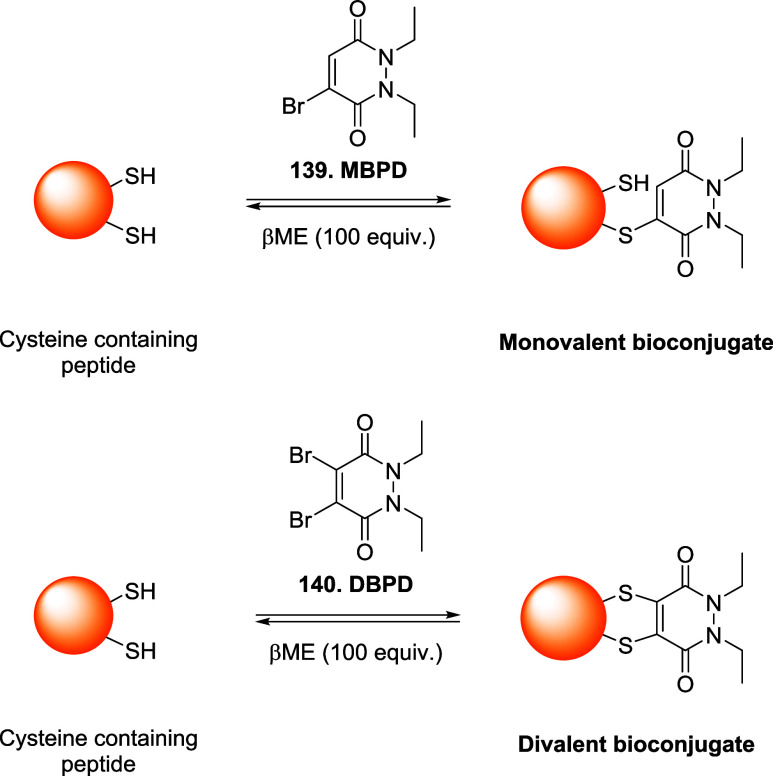
Bioconjugation Reactions Realized with a Bromo- or a Dibromopyridazinediones

Evidence of their usage in reversible bioconjugation chemistry was reported in 2011 by Chudasama and co-workers, demonstrating that BrPDs selectively modify cysteine residues in proteins with high efficiency and hydrolytic stability, triggering the reversibility under physiologically relevant reducing conditions (*e.g.*, 100 equiv βME).^101^ The authors, using a cysteine mutant of the Grb-2-SH2 domain as a model protein to assess the efficiency of the BrPD scaffold, identified the *bis­(N*-ethyl)­bromopyridazinedione (**139**, **MBPD**) as the most efficient tag at 37 °C for 1 h yielding a quantitative cysteine modification. The MBPD-protein adduct was completely stable at 37 °C for 5 h, addressing the potential hydrolysis liability. Reversibility was then introduced by treatment with βME or GSH (1 mM). These last findings suggest that this scaffold could be involved in the preparation of prodrug systems; in fact, the design could be optimized to cleave in the cytoplasm of mammalian cells that contain a relatively high concentration of GSH. Ellman’s test showed no residual free cysteines despite 8 lysine residues (high cysteine selectivity). The authors describe in the same study also labeling of disulfide bonds using diBrPDs (**140**, **DBPD**). DiBrPDs have emerged in the past decade as a class of disulfide-bridging reagents with interesting potential, as they are compatible with common mild reducing reagents. The reaction of a diBrPD with two thiols leads to a divalent conjugate; both bromides are displaced, and two thioether bonds are formed, allowing diBrPDs to bridge two cysteine residues. Unlike bromomaleimide linkers, diBrPD conjugates are generally nonlabile under physiological conditions; in fact, the resulting bis-thioether linkage is resistant to hydrolysis. Exploring the features of the diBrPD scaffold, Maruani et al. reported another relevant case study where they were integrated into an antibody to generate rebridged disulfide linkages that preserved antibody integrity while introducing orthogonal “click” handles.^54^ This led the authors to the generation of an antibody dual bioconjugate (Her-Astra-Dox-Cy5) containing both a cytotoxic drug (doxorubicin) and an imaging agent (Cy5) that retained the antigen binding. Similarly, Bahou et al. established an efficient and scalable route to a series of functionalized diBrPDs, and systematically applied them to trastuzumab disulfide rebridging.^30^ In this work, they developed a new synthetic route via a dibromopyridazinedione-NHS ester, aiming to install different amines on the *N*-substituted alkyl chain. Their approach yielded over 90% homogeneous conjugation with minimal disulfide scrambling and without requiring protein engineering or enzymatic modification. This level of control in the bioconjugation processes overcomes the heterogeneity typically observed with lysine- or cysteine-targeting maleimide linkers, which often result in variable drug-to-antibody ratios and inconsistent pharmacokinetic behavior. In a follow-up study by the same author, the biophysical performance of diBrPD-based ADCs was further assessed examining how disulfide rebridging can affect Fc-region functionality.^102^ Using trastuzumab as a model IgG1, they demonstrated that natively rebridged antibodies retain full thermal stability, target binding, and CD16a interaction (essential for maintaining the antibody half-life and effector function). Importantly, constructs with disrupted or misaligned rebridging exhibited diminished Fc activity. In both cases, the studies reported here by Bahou et al., the reversible nature of the BrPD scaffold was unchanged from the previous reports and always required reductive conditions to be triggered.

## 4 1,2-Addition Reactions

The 1,2-addition reactions represent a specific category of thiol-selective bioconjugation processes. In these reactions, the sulfur atom of a cysteine residue engages in a direct nucleophilic attack on an electrophilic carbon-heteroatom multiple bond, resulting in the formation of a covalent adduct. The reversibility of these systems is governed by the electrophilicity of the reaction center and the thermodynamic stability of the resulting adduct, which in turn are modulated by factors such as conjugation with electron-withdrawing groups, the presence of intramolecular coordination sites (*e.g.*, boron–nitrogen interactions), and the ability of the product to undergo tautomeric or prototropic rearrangements that facilitate thiol exchange or hydrolytic cleavage.^103–107^ Several innovative classes of bioconjugation reagents have been developed that exploit this mechanism; among the most representative are α-substituted nitriles, iminoboronates, isoxazolinones, and triazines (Figure 13).

**Figure 13:**
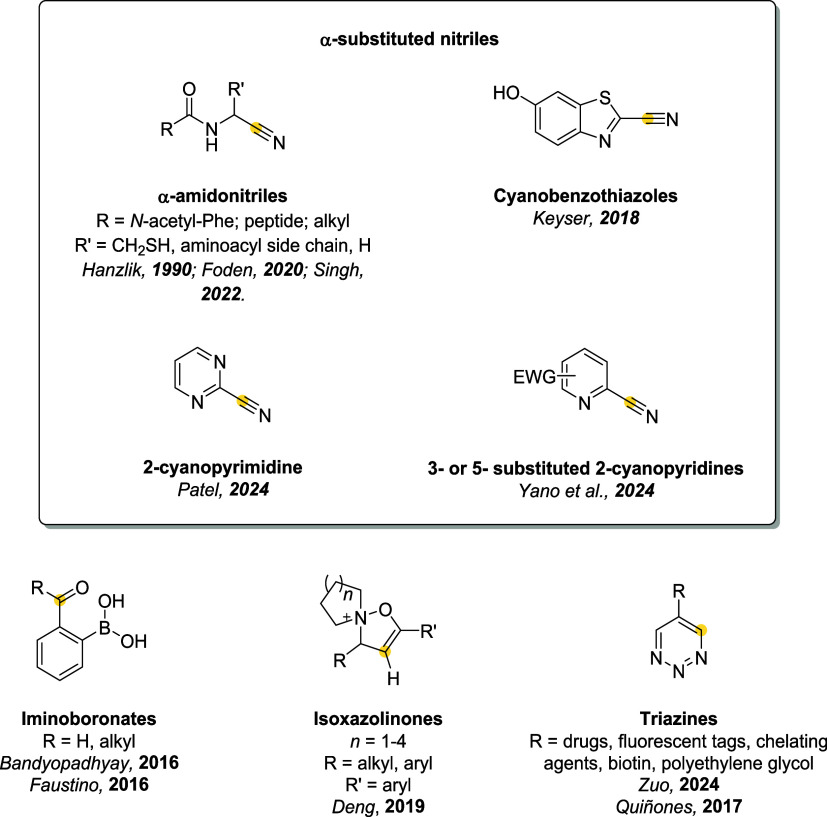
Different classes of thiol-selective scaffolds documented for reversible conjugation via a 1,2-addition mechanism. Bioconjugation sites are marked with a yellow dot.

Each of these linkers enables distinct applications ranging from selective peptide modification to reversible intracellular tracking.^103,105–107^ In the following subsections, each class of linker will be described in detail, with an emphasis on the underlying reaction mechanism, the factors influencing reactivity and reversibility, and their applicability to bioconjugation under physiological conditions.

### 4.1 α-Substituted Nitriles

The α-substituted nitriles are a widely studied class of thiol-selective linkers that exploit the formation of a thioimidate intermediate to impart reversibility to the system under different conditions (thioimidate intermediate in Scheme 24).

**Scheme 24:**
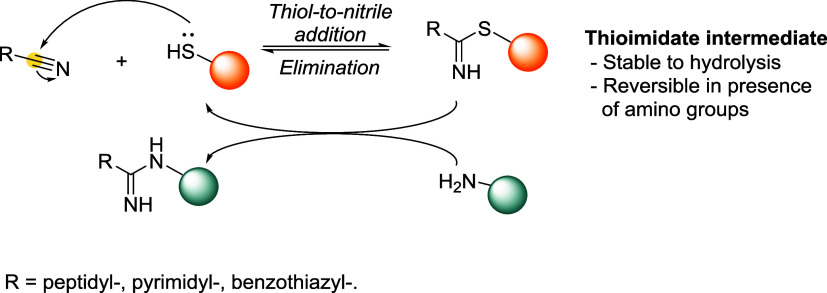
1,2-Addition general mechanism for α-substituted nitriles in the presence of amino groups

They have been reported as thiol-selective linkers originally by Hanzlik et al. in 1990, where the authors used a model cysteine protease (papain) as thiol-source to realize a 1,2-addition on the nitrile carbon of an α-substituted nitrile, generating a thioimidate intermediate (Scheme 24).^108^ This intermediate resulted in being a key component to impart reversibility to the system, thanks to the lability of the thioimidate intermediate toward spontaneous elimination reaction of the thiol. The authors tested 12 peptide-like nitriles and closely related non-nitrile analogues (as controls), reacting them with papain and extrapolating *K*_d_ values to assess the efficiency of the bioconjugation. Crucially, non-nitrile controls showed no measurable binding under identical conditions, highlighting the role of the nitrile moiety in imparting selectivity to the system. In terms of mechanism, the thiolate on papain performs 1,2-addition to the electrophilic carbon of the nitrile, forming the thioimidate intermediate, which reversibly dissociates to regenerate the free nitrile and enzyme. Another study by Keyser et al. in 2018 extended the biological relevance of α-substituted nitriles to cyanobenzothiazole (CBT) derivatives under physiological conditions.^109^ In this study, the authors set out a short encodable peptide motif containing a cysteine-lysine pair positioned so that after the initial cysteine conjugation with CBT (**141**), the lysine could attack intramolecularly through an S–N transfer, stabilizing the adduct and liberating the free thiol (Scheme 25).

**Scheme 25:**
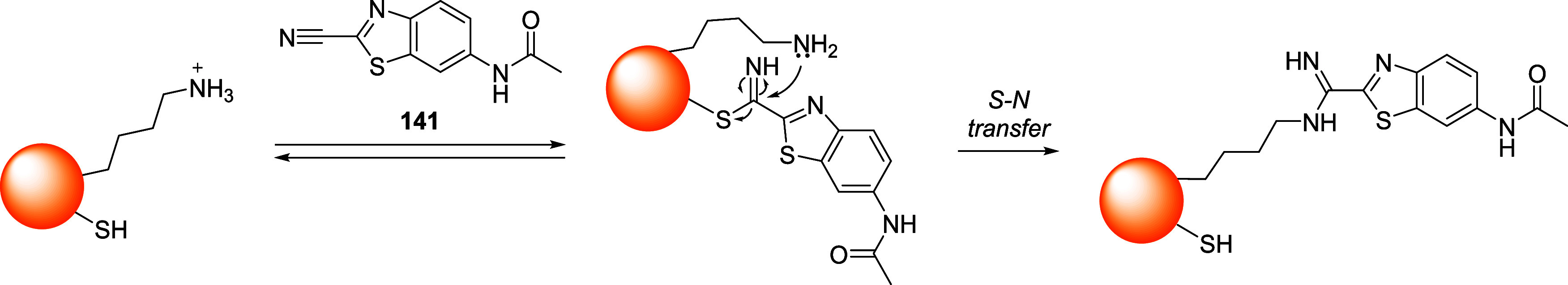
General Mechanism for the Cysteine–Lysine Interaction during the Conjugation of a Peptide with CBT

This second case study is not mechanistically different from the first one; it forms the already reported thioimidate intermediate while at the same time introduces a second type of reversibility given by the proximal lysine ε-amine that attacks the thioimidate-carbon, generating a stable amidine via nucleophilic substitution reaction (Scheme 25). Once the amidine is formed, in fact, the bioconjugation of lysine was ‘locked in’ and overall irreversible. Because the initial cysteine-nitrile adduct is reversible, it functions as a proofreading step: off-target cysteines dissociate, whereas only the Cys–Lys motif promotes intramolecular S→N transfer to an amidine, locking the modification. A comprehensive kinetic survey involving CBTs is provided by Proj et al., which maps how heteroaromatic α-substituted nitriles tune the bioconjugation under physiological buffer.^110^ As a matter of fact, 116 heteroaromatic α-substituted nitriles have been analyzed both in their aqueous stability (pH 7.4, 37 °C) and in their second-order rate constants for cysteine addition, revealing 3 orders of magnitude variation in reactivity. Electron-withdrawing substitution on the heteroaryl core accelerated the cys–nitrile addition, whereas electron-donating groups attenuated it; as a practical benchmark, achieving complete low-μM labeling within ∼30 min generally required *k*_2_ > 5 M^–1^s^–1^. Mechanistically, all series proceed by reversible thioimidate formation, but *N*-terminal Cys undergoes intramolecular capture to a thiazoline (irreversible under the assay), whereas internal Cys gives only the reversible thioimidate. Advancements in this regard have been reported by Foden et al. in 2020, while they proceeded to question whether cysteine played an important role as a catalyst in the nonenzymatic ligation of α-amidonitriles in prebiotic conditions.^111^ The authors identified high-yielding prebiotic routes to cysteine peptides, demonstrating that reversible thiol-nitrile chemistry could have powered early peptide bond formation through a process called Catalytic Peptide Ligation (CPL). This resulted to be a key component for understanding prebiotic life; in fact, the outcome of this cysteine-based catalysis would have been peptidyl amidines (when the nucleophile is amino acid) or peptides (when nucleophile is a peptide or amide) as will be demonstrated by Singh and co-workers in 2022.^112^ This follow-up study was necessary to justify the formation of ‘true’ peptide bonds from the chemistry previously described. Specifically, they demonstrated that if the nucleophile is a peptide, the S–N transfer could lead to an intramolecular amide-catalyzed hydrolysis, resulting eventually in a peptide bond formation (**148**). Interestingly, cysteines catalyze the ligation of α-amidonitriles with amino acids or amides under neutral (or mildly alkaline) aqueous conditions, and the reaction was specific for α-amidonitriles, whereas β- and γ-nitriles reacted poorly (Scheme 26).

**Scheme 26:**
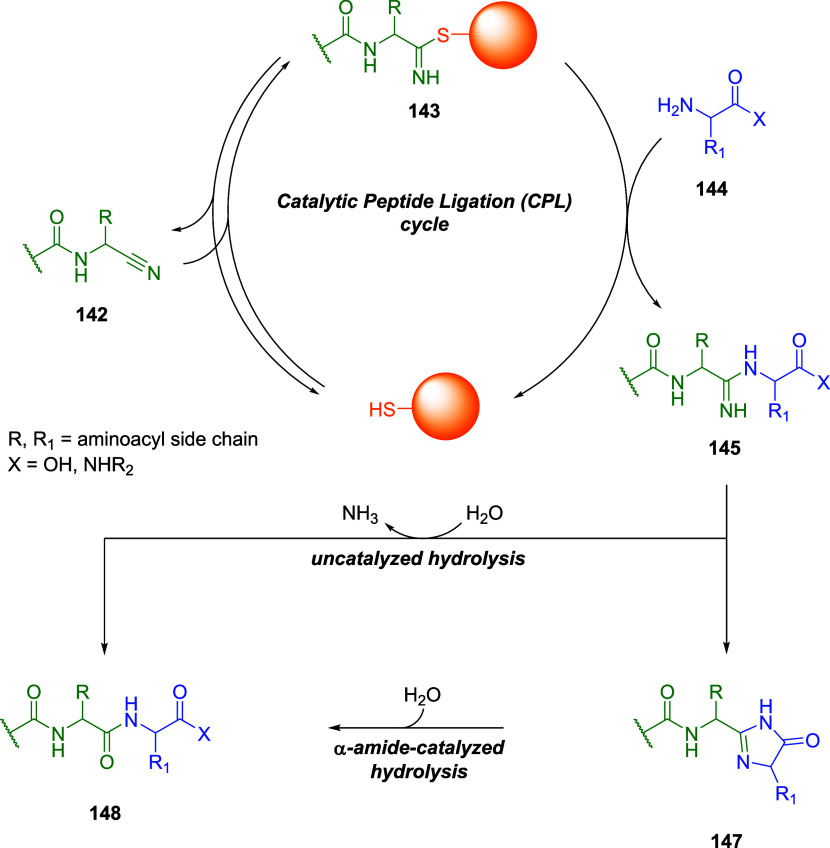
Graphical Representation of the Catalytic Peptide Ligation (CPL) Cycle

Another type of α-substituted nitriles consists in cyanopyrimidines, a class of heteroaryl nitriles that exhibit selective reactivity toward cysteine residues, particularly toward *N*-terminal cysteines.^113^ The mechanism evolves through two different steps: the 1,2-addition of the thiol group of cysteine to the electrophilic carbon of the cyano group, forming a thioimidate intermediate;^104^ then, in the presence of a neighboring thiol, a rapid intramolecular attack occurs, yielding a five-membered dithiolane ring (the cyclization step is crucial for product stability and selectivity). The reaction benefits from the electron-withdrawing nature of the pyridine ring, particularly when substituted at the 3- or 5-position with additional electron-withdrawing groups, which can enhance the reactivity of the cyano moiety toward thiols.^112^ Importantly, as reported by Patel et al., treating the isolated aminodithioacetal (ADTA, **151**) conjugate with a fast thiol scavenger, *N*-methylmaleimide (3 equiv), sequesters both thiols and drives full back-conversion to the heteroaryl nitrile (**149**) within ∼2 h in PBS pH 7.4 at 22 °C, underscoring that ADTA **151** formation is reversible and can be toggled chemically (Scheme 27).^114^ The described nitrile bis-thiol (NBT) chemistry was evaluated in terms of antibody conjugation. The work reports reactions of heteroaryl nitriles with reduced antibody disulfide bonds, efficiently pairing cysteine residues, leading to distinct conjugation outcomes depending on the structural design of the nitrile reagent. The formation of either cysteine-to-lysine transfer products or disulfide-bridged NBT conjugates was observed, depending on the environmental surroundings of the targeted cysteine residue, and stable in the presence of glutathione, demonstrating that the NBT reaction constitutes an efficient and tunable bis-thiol conjugation strategy.

**Scheme 27:**
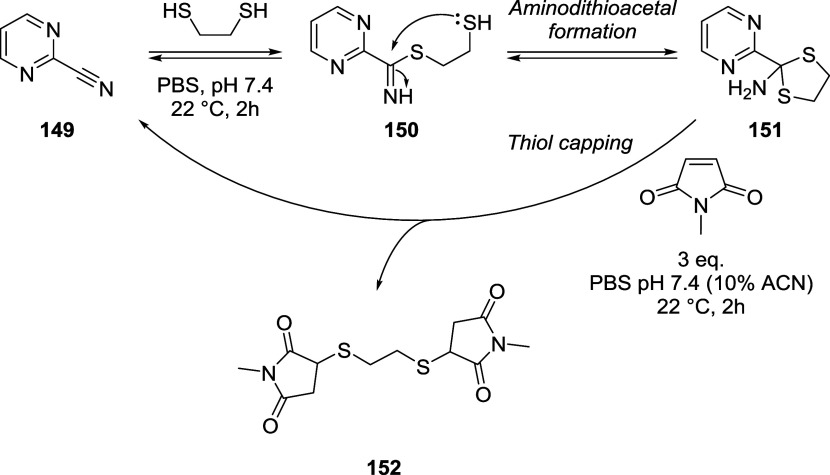
Representation of the Mechanism of ADTA Formation

Yano et al. reported a library of modified 2-cyanopyridine derivatives designed for site-selective conjugation to the *N*-terminal cysteine of glutathione.^113^ Their study revealed that the reaction proceeds under mild aqueous conditions with excellent chemoselectivity, resulting in a thiazoline adduct. Importantly, in the case of glutathione, the adduct undergoes hydrolytic cleavage at the peptide bond, indicating a potential application for controlled degradation.

### 4.2 Iminoboronates

Iminoboronates are efficient intermediates for selective, rapid, and reversible *N*-terminal cysteine functionalization.^105^ The mechanism proceeds via the condensation of an *o*-formyl (or *o*-acyl) aryl boronic acid with a 1,2-aminothiol moiety, forming a Schiff base intermediate that is subsequently stabilized through intramolecular B–N dative bonding (**155**). This interaction effectively improves the electrophilicity of the benzylic carbon while simultaneously locking the conformation, thus conferring high kinetic and thermodynamic stability. This chemistry proceeds under physiological conditions and displays excellent selectivity for *N*-terminal cysteine residues due to the spatial proximity of the amine and thiol functional groups. Mechanistic investigations, including spectrophotometric monitoring and ^11^B NMR studies, confirmed the reversibility of the B–N coordination and the influence of electronic effects on adduct stability.^114^ The reaction mechanism, featuring imine condensation followed by boron–nitrogen stabilization, is illustrated in Scheme 28.

**Scheme 28:**
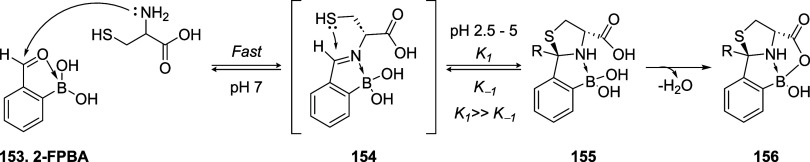
Representation of the Mechanism of Cysteine Functionalization with 2-Formylphenylboronic Acid (2-FPBA)

Although initially identified as useful tools in the lysine-based bioconjugation chemistry, iminoboronates were investigated by Bandyopadhyay and co-workers with regard to their chemoselectivity for the 1,2-aminothiol moiety (such as cysteine).^103^ The authors demonstrated that 2-formylphenylboronic acid (2-FPBA, **153**) derivatives react specifically and reversibly with *N*-terminal cysteine to give a thiazolidino-boronate (TzB) adduct (**155**); since ordinary internal cysteine residues lack the vicinal amino group, the reaction can be reversed, and the starting materials regenerated. Interestingly, the reaction was diastereoselective, yielding quantitatively compound **155**; this selectivity is thought, as stated in the work, to be due to the preorganization imparted to the structure by the B–N bond formation, favoring the attack of the imine from the top face to give the single diastereomer observed. In their work, they also evidenced the important role played by the boronic acid that promotes facile thiazolidine formation at neutral pH, an important feature in biorthogonal chemistry. Faustino et al. provided key insights into the chemoselectivity and reversibility of iminoboronate formation using FPBAs and model peptides bearing *N*-terminal cysteine residues.^105^ Their work established that iminoboronate conjugation can proceed quantitatively within minutes under physiological conditions with excellent site selectivity and minimal off-target reactivity. Combining NMR spectroscopy, mass spectrometry, and kinetic studies, the authors characterized the stability and dynamic reversibility of the formed adducts. They also demonstrated the compatibility of this chemistry with fluorogenic probes and biomolecule labeling, expanding the applications toward real-time monitoring and reversible bio-orthogonal tagging. Importantly, reversibility was shown to be pH-dependent and could be triggered under acidic conditions, highlighting the utility of this approach for intracellular delivery and release applications. Notably, this study showed that changes in the aryl boronic acid scaffold can affect reactivity and selectivity: fluorination of the aromatic ring had a negligible impact on the reaction rate (**158**), whereas replacement with a thiophenyl boronic acid reduced both efficiency and diastereoselectivity (**159**) (Figure 14).

**Figure 14:**
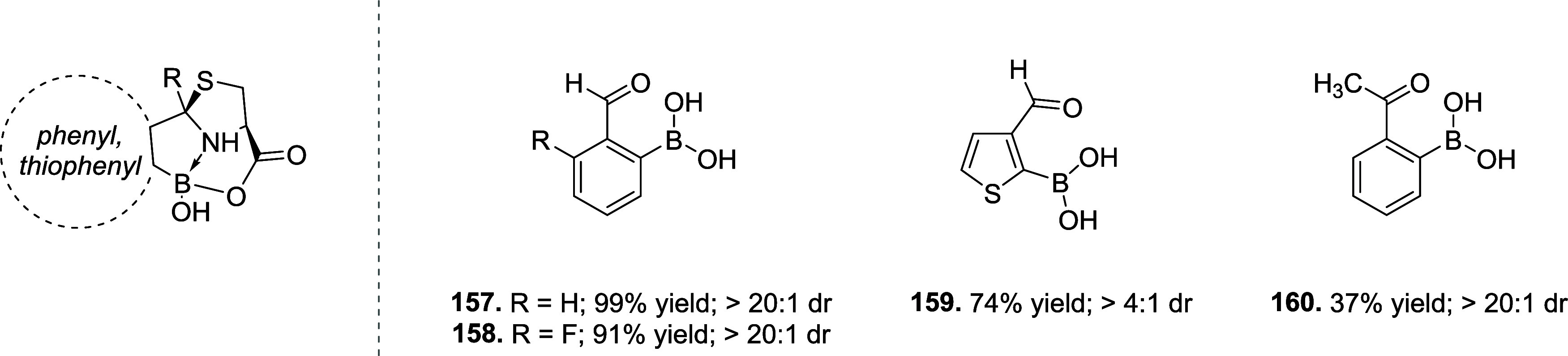
Left panel: representation of the main diastereoisomer formed upon reaction between substituted aryl boronic acids **157–160** and *N*-terminal cysteine; right panel: representation of the changes in the aryl boronic acid scaffold.

### 4.3 Isoxazolinones

Isoxazolinium-derived reagents have recently emerged as a novel class of heterocyclic electrophiles enabling chemoselective and reversible covalent modification of cysteine residues.^81^ In a seminal study, Deng and co-workers systematically developed and evaluated a comprehensive library of 25 structurally diverse isoxazoliniums, comprising 21 monofunctional derivatives, two fluorescently labeled variants, and two bis-reactive species for cysteine–cysteine macrocyclization, for their suitability as thiol-reactive bioconjugation linkers under physiologically relevant conditions (pH 7.4, 25 °C).^105^ The isoxazolinium scaffold is generated in situ from propargylamine *N*-oxides via silver­(I)-catalyzed oxidative cyclization using *m*-chloroperbenzoic acid, yielding an electrophilic center at the C4-position of the isoxazoline ring (Figure 15).

**Figure 15:**
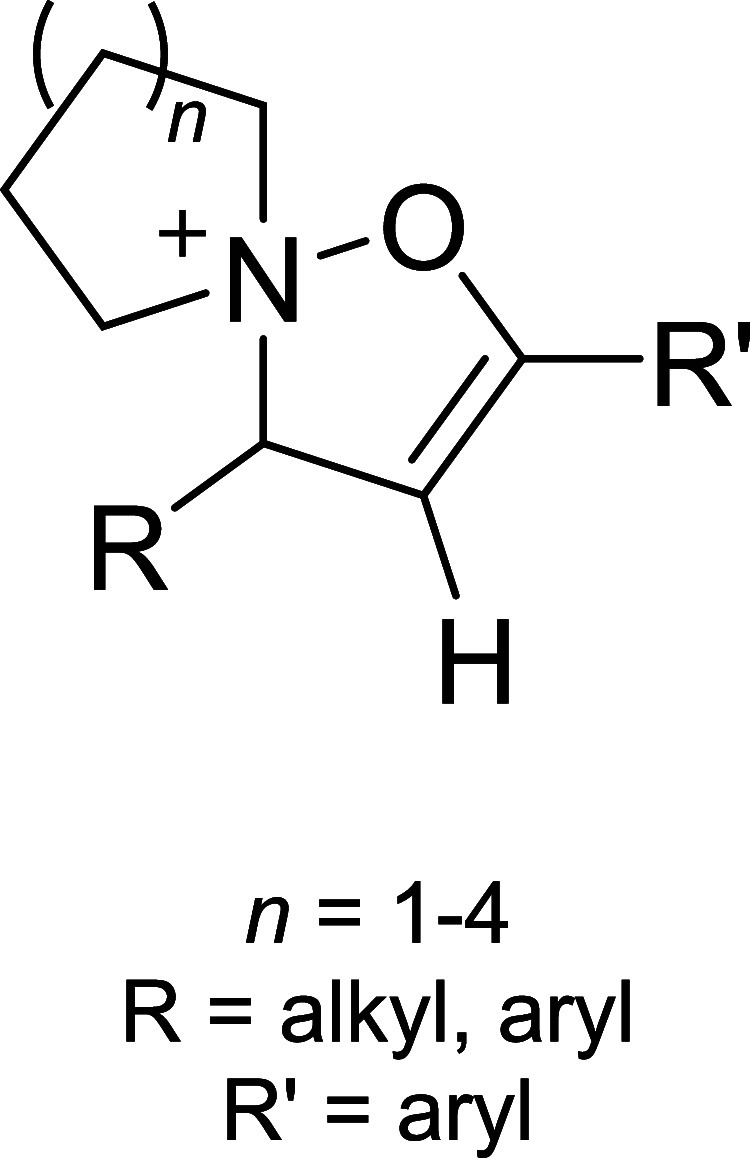
General structure for the isoxazolinones scaffold.

This position, flanked by a conjugated carbonyl and a ring nitrogen, exhibits enhanced electrophilicity, rendering it particularly susceptible to 1,2-nucleophilic addition by cysteine. Under optimized conditions, 5 mol % AgNO_3_ and stoichiometric amounts of isoxazoliniums in aqueous–organic buffer (PBS/CH_3_CN, 19:1), site-selective modification of cysteine residues proceeded with high efficiency (up to >99% conversion) and minimal side reactions. Notably, increasing the amount of AgNO_3_ from 1% to 5% corresponded to an increase of 10% in yield. Moreover, screening reactions in the same buffer at different pH values indicate excellent conversion from slightly acidic to basic media (pH from 5 to 9). As expected, peptides lacking cysteine remained unmodified, and no cross-reactivity was observed with other nucleophilic residues (*e.g.*, lysine, histidine, and methionine), underscoring the intrinsic chemoselectivity of the platform. Six-membered cyclic amines (*e.g.*, piperidine and cyclohexylamine) were identified as optimal for achieving high reactivity and stability (Scheme 29).

**Scheme 29:**
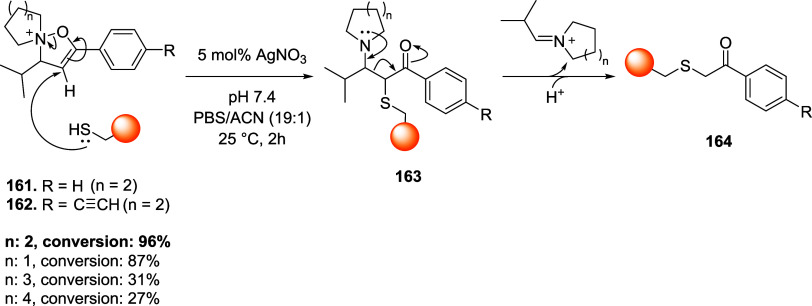
General Mechanism for the Formation of Conjugate **164**

Introduction of various substituents at the aryl *para* position permitted modulation of the conjugation rate and, critically, the kinetic lability of the resultant thioether adducts (**161** and **162**). Of particular interest were the alkyne-functionalized derivatives (**162**). In this regard, the authors used alkyne-functionalized reagents to insert a terminal alkyne directly at cysteine, creating a phenylacyl thioether that served as an orthogonal click handle for postlabeling and a built-in accelerator for UV-A photocleavage. During the photolysis experiments conducted at 365 nm, the alkynyl phenylacyl thioether cleaved much faster than its nonalkynyl analogue (alkynyl analogue >90% in 10 min; >99% in 15 min compared to nonalkynyl analogue: 41% in 15 min), and on proteins >60% of linkages were removed in 30 min. Mechanistic studies revealed that irradiation at λ = 365 nm (UV-A) initiates a Norrish type II cleavage at the aryl–sulfur bond, resulting in quantitative formation of a thioaldehyde intermediate **166** (Scheme 30). This intermediate could subsequently be reduced to the native thiol using NaBH_4_, thereby completing a traceless deconjugation sequence.

**Scheme 30:**
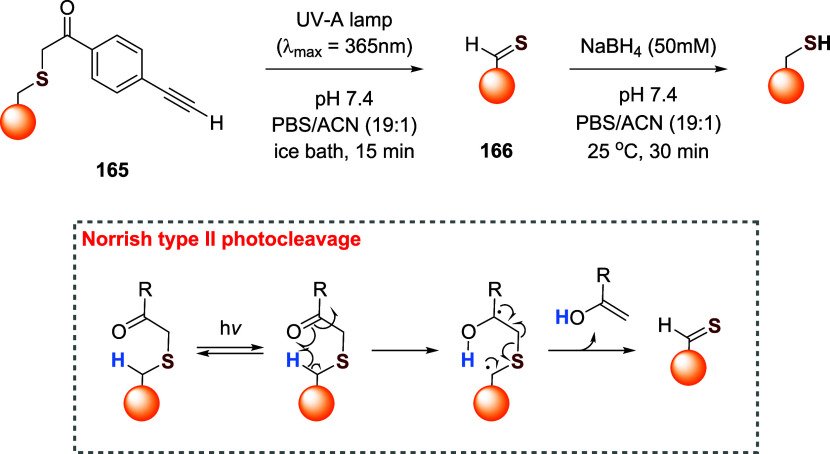
General Mechanism for the Norrish Type II Cleavage of Conjugate **165**

The cleavage efficiency was shown to be substituent-dependent: alkynylated conjugates such as **165** underwent >90% cleavage within 10 min, while unsubstituted analogues displayed only partial cleavage (ca. 24%) over the same interval. Critically, control experiments conducted in the absence of light showed no cleavage, confirming that the process is photoinduced. This two-step deconjugation mechanism, a photolytic fragmentation followed by chemical reduction, offers precise temporal control over protein labeling and release.

### 4.4 Triazines

The last class of linkers illustrated in this section is the one of 1,2,3-triazines, reported by Sun and Wang.^115^ These molecules are quite interesting in the field of protein bioconjugation due to their high selectivity toward cysteine and their versatility in multiple biorthogonal transformation. In fact, if properly designed, these compounds offer the possibility to perform singular or double bioorthogonal modification of biomacromolecules. The conjugation proceeds with the nucleophilic attack of thiol at position 4 of the triazine (**167**), forming a dihydrotriazine intermediate (**168**), which undergoes spontaneous nitrogen elimination to give an α,β-unsaturated imine (**169**) (Scheme 31). This intermediate can be isolated and detected by LC-MS analysis, but it is usually hydrolyzed to an aldehyde by the basic reaction medium.^116^ Since the product is an α,β-unsaturated aldehyde, the thiol source can be regenerated with an excess of an additional thiol, such as GSH, like in the case of the linkers that are conjugated via an addition–elimination mechanism.

**Scheme 31:**
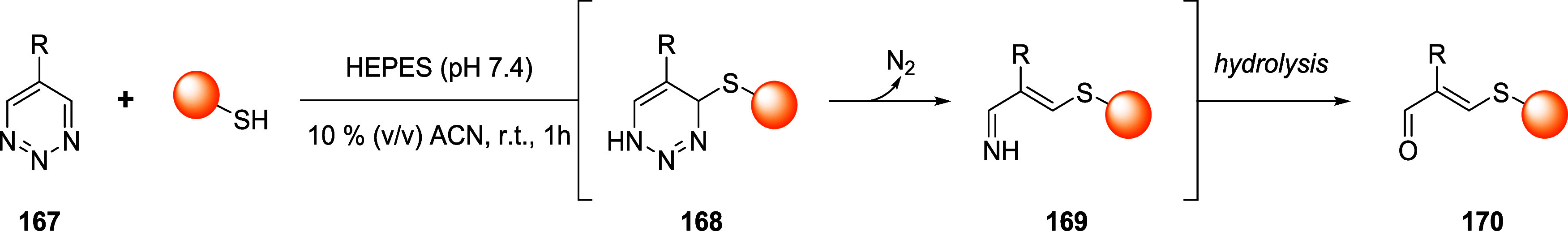
General Reaction Mechanism for Cysteine Bioconjugation by Means of 1,2,3-Triazines

The authors started this study with a triazine linker functionalized with an amide in position 5 (**171**), which increased in a highly electrophilic compound with low selectivity toward cysteine in a conjugation attempt with GSH (Figure 16). To improve thiol modification, a less electrophilic 5-phenol triazine (**172**) was synthesized and subjected to conjugation with GSH, under different reaction conditions. The best conditions were established by performing the reaction at room temperature in HEPES buffer at pH 7.4 with 10% of ACN as cosolvent. Changing the buffer (excluding Tris) did not affect the efficiency of the reaction, whereas switching to pure water decreased the yield to 14%. Moreover, different pH values were screened with the conjugation proceeding smoothly at neutral and weakly alkaline pH, in contrast with lower yields in slightly acidic media. After having assessed the optimal reaction conditions, triazine B was employed for the bioconjugation of several peptides, including short, long (up to 20mer), cyclic, bulky, and human health relevant peptides (one from the sequence of histone H2A, the HPV-E6-C peptide, and the WSCO2 peptide), showing excellent selectivity toward cysteine and good reaction yields. The authors then proceeded by designing different compounds (**173–183**) and testing them toward a cysteine-containing model peptide, observing that the reaction proceeds smoothly for all the tested triazines (Figure 16). Interestingly, all the tested linkers reacted with the model peptide, giving the change of introducing several important groups, such as drugs (**176**), fluorescent tags (**177**, **178**), chelating agents (**179**), endomorphin-2 (**180**), poly­(ethylene glycol) (**181**), biotin (**182**), and click handles like tetrazine (**183**). In addition, bitriazines **184–186** were employed for a double functionalization of cysteine, both for dimerization and cyclization of peptides.

**Figure 16:**
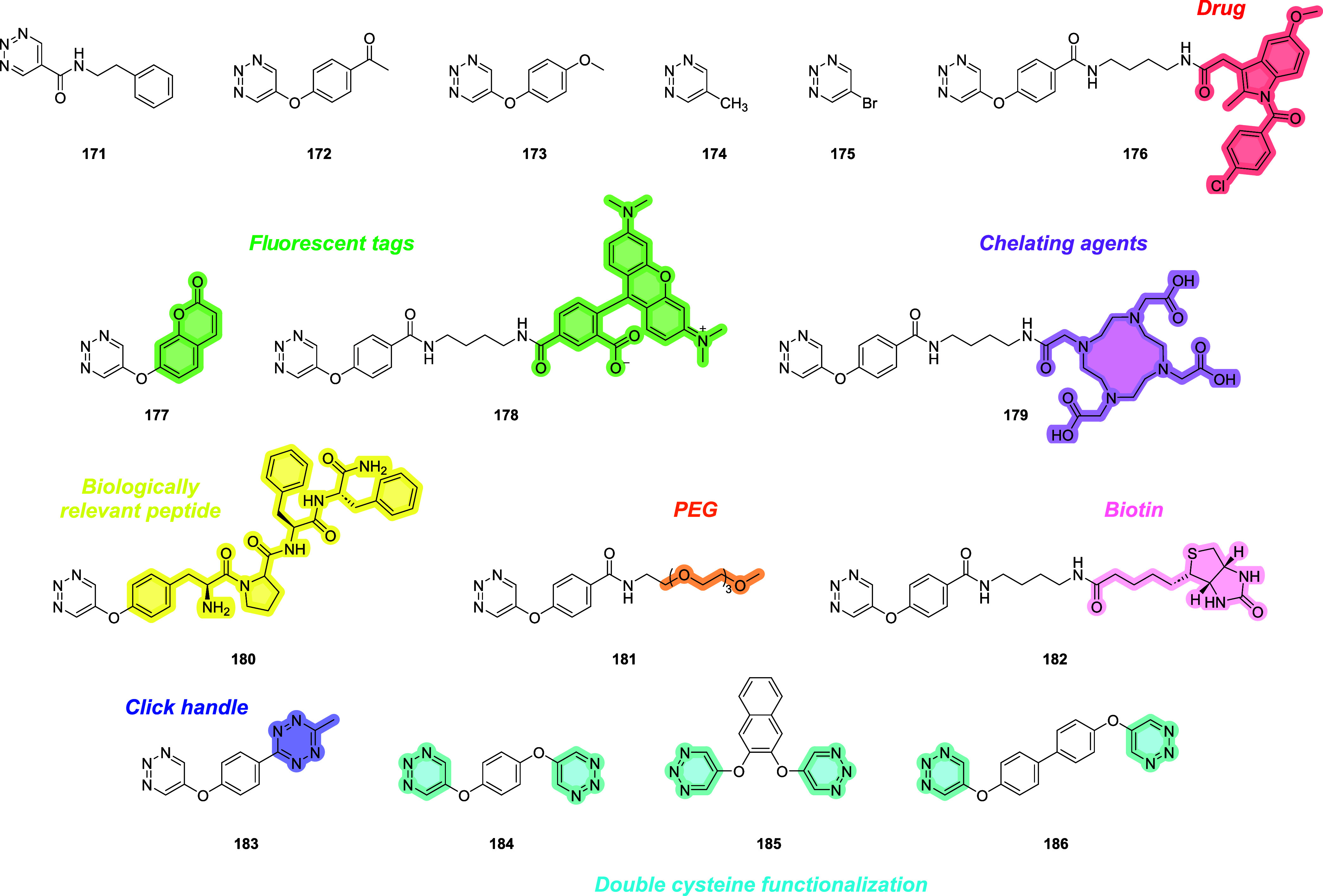
Structure of 1,2,3-triazine linkers **171**–**186**.

The stability of the modified peptides was assessed with the conjugate between **173** and the cyclic peptide *c*-(RGDFC), being stable for 72 h at pH between 3.0 and 7.4 and less stable at higher pH values (9 and 11). The conjugate resulted in being stable even in the presence of H_2_O_2_, showing only 15% decomposition after 24 h of incubation. As previously anticipated, incubation with excess GSH led to decomposition, completely regenerating the parent peptide after only 12 h. The most interesting application of the conjugation strategy studied by Sun and Wang consists in secondary and tertiary functionalization of the target thiol source. In this experiment, a short peptide from the sequence of Chorionic Gonadotropin-β was reacted in the optimized conditions with triazine **183** to obtain α,β-unsaturated aldehyde **187**, which can be further functionalized on the tetrazine moiety with BCN–OH by IEDDA to obtain double functionalized compound **188** (Scheme 32). The author then exploited the aldehyde handle to attach an affinity target such as biotin through hydrazone formation by biotin hydrazide, obtaining the triple functionalized compound **189** with a 46% yield over 3 steps.

**Scheme 32:**
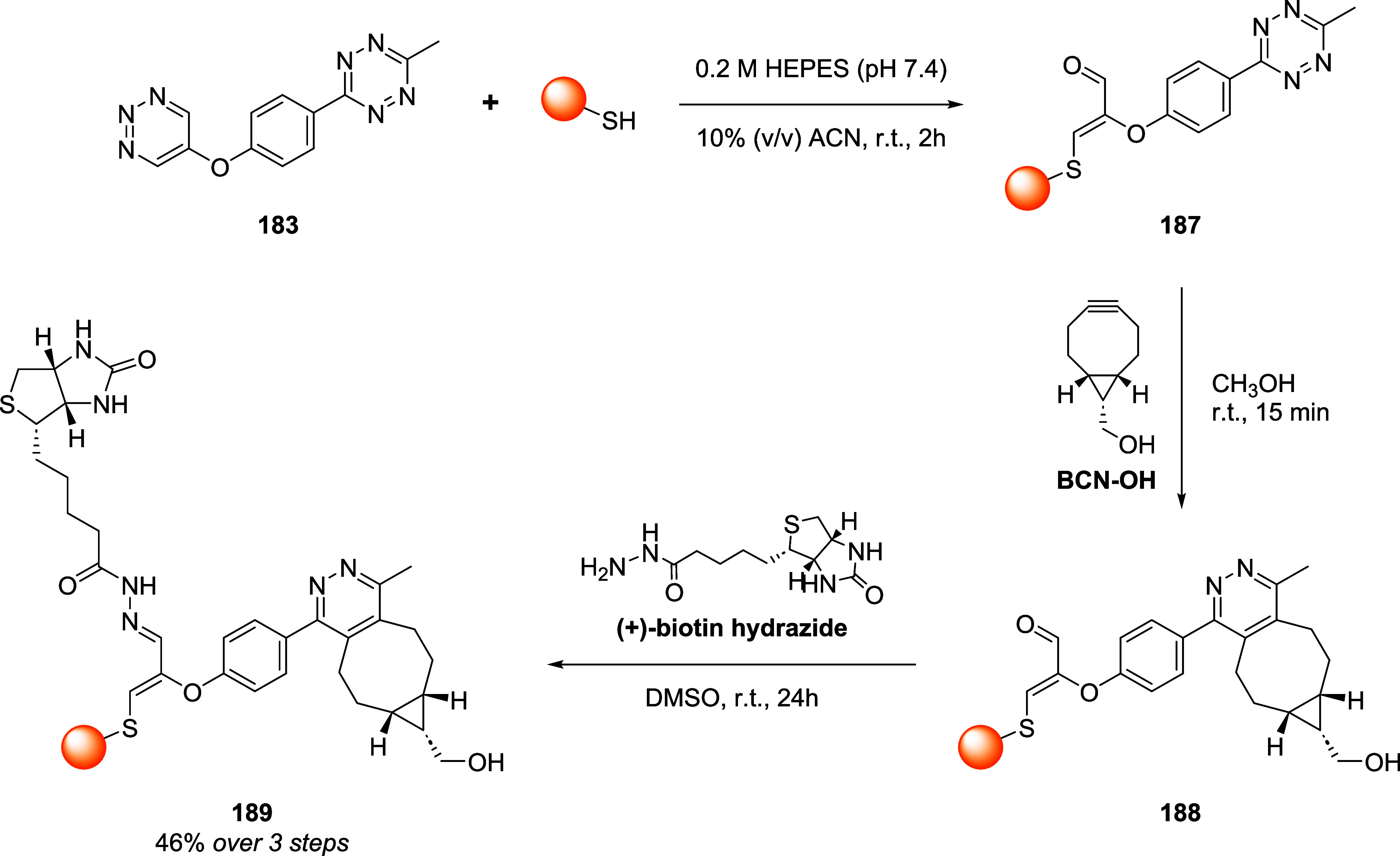
Triple Functionalization of Triazine **183** by Thiol Addition (**187**), IEDDA (**188**), and Hydrazone Formation (**189**)

## 5 Aromatic Nucleophilic Substitutions (S_N_Ar)

Aromatic nucleophilic substitutions are a class of highly versatile reactions for the functionalization of various aromatic substrates in organic synthesis. Almost uncharted until recently, arylation reactions were considered unsuitable for bioconjugation due to the lack of well-developed chemistry on biomolecules.^117^ In contrast, these reactions proceed under mild conditions, they are suitable for biorthogonal transformations, and they are very versatile since the reactivity of their substrates can be easily tuned, as will be shown in the next few examples. Several innovative classes of aromatic compounds that exploit this mechanism have been developed; the ones reported in this review are pyridinium ions, tetrazines, heteroaryl azoline thioethers, and aryl thioethers (Figure 17).

**Figure 17:**
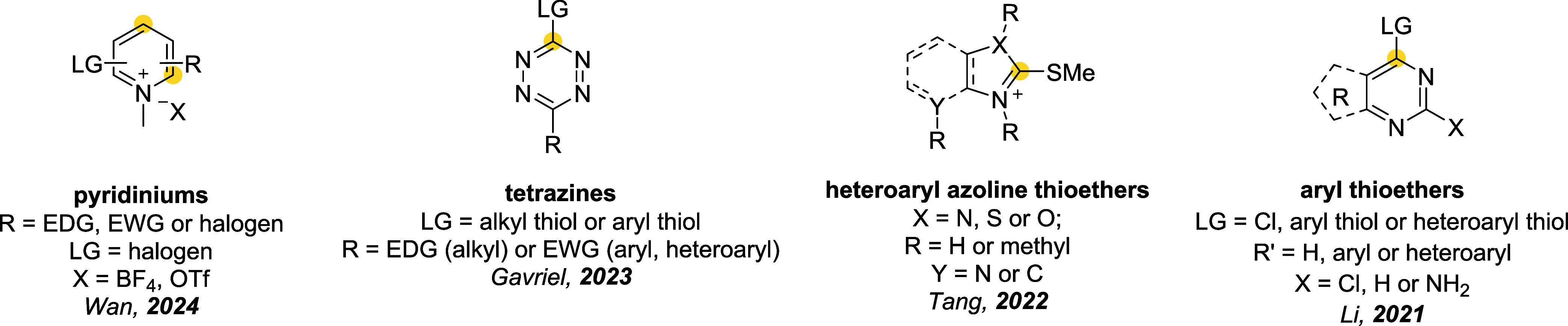
Different classes of thiol-selective scaffolds for the reversible conjugation via the S_N_Ar mechanism. Bioconjugation sites are marked with a yellow dot.

In this section, a general reaction mechanism for the addition-elimination mechanism (S_N_Ar) and the corresponding reversible deconjugation reaction is reported (Scheme 33) by using substituted *N*-methylpyridinium ions as substrates, which is the scaffold employed by Wan et al., whose work will be described later.^118^ In the reaction mechanism, the substrate undergoes nucleophilic attack by a nucleophile, in this case a thiol, forming an intermediate called *σ*-complex or Meisenheimer complex. In the case of neutral electrophiles, the Meisenheimer complex is an anionic species, whereas in the case of cationic electrophiles—like pyridinium ions—this intermediate is neutral. The aromaticity of the molecule is then restored by expulsion of the leaving group, affording the product. The substitution pattern of the electrophile is crucial for the kinetics of the reaction: electron-poor aryl compounds or heterocycles, with the exception of pyrrole, furan, and thiophene, stabilize the negative charge of the Meisenheimer complex, thus favoring the reaction; on the other hand, electron-rich aryl compounds do not stabilize the negative charge in the Meisenheimer complex, making the substrate unreactive toward S_N_Ar. For these reasons, the presence of EWG in the *ortho* or *para* positions with respect to the leaving group is very important.

**Scheme 33:**
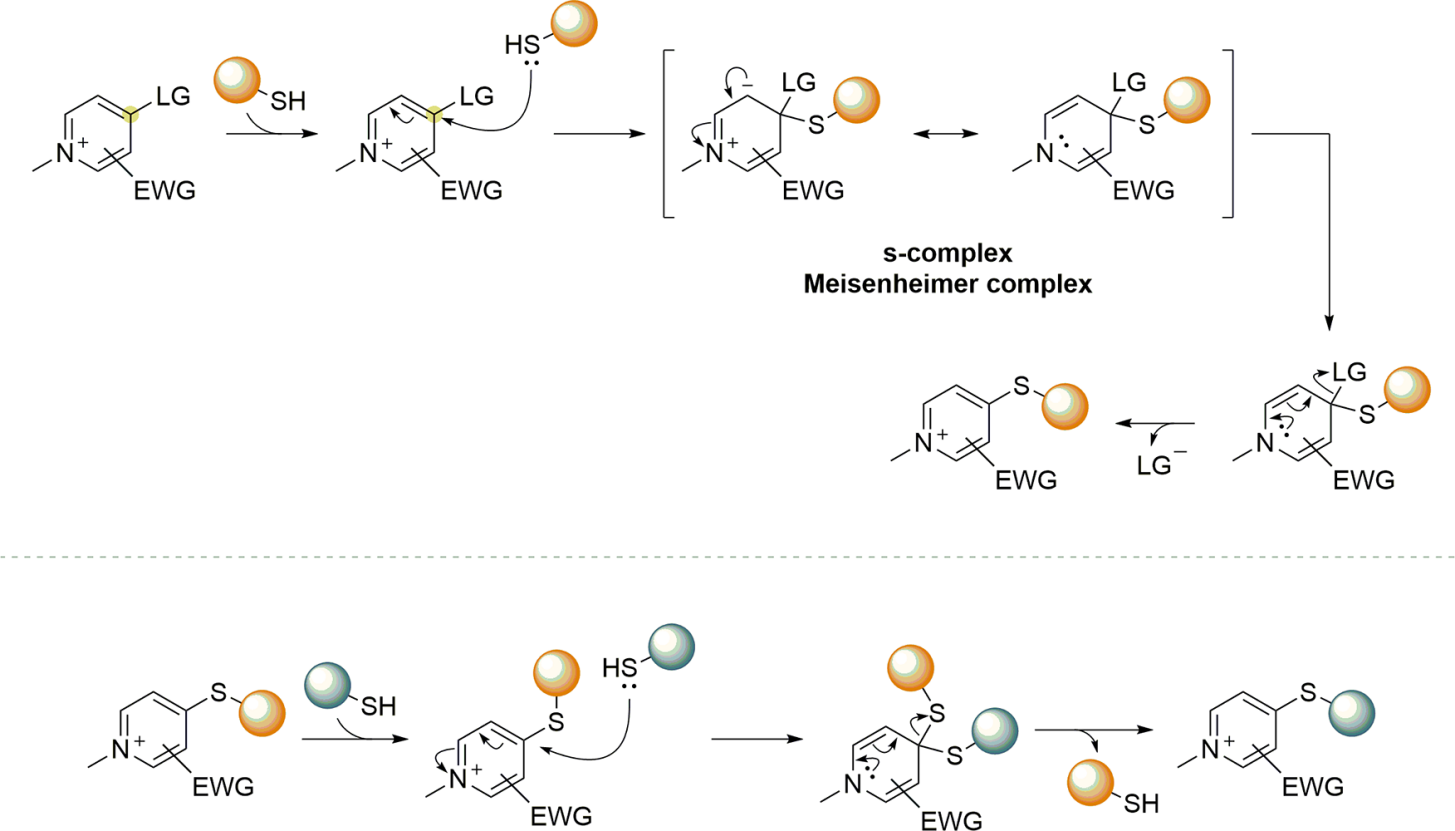
General S_N_Ar Reaction Mechanism for Thiol Bioconjugation^a^

a Bioconjugation site is marked with a yellow dot.

In their paper, Wan et al. reported the synthesis of several substituted mono- and dihalogenated pyridinium ions bearing EWG and EDG substituents, thus representing a perfect example of tunability of the S_N_Ar reaction.^118^ These compounds were employed to investigate the conjugation of peptides and proteins as well as the stapling of peptides by the dihalogenated linkers (Figure 18). The model study was carried out by reacting 2- and 4-halogenated pyridinium ions with acetylcysteine, single cysteine- and double cysteine-containing peptides, and native proteins, obtaining second-order rate constants *k*_2_ ranging from 1.6 × 10^5^ to 0.17 M^–1^ s^–1^. Electronic effects were evaluated by reacting 2-halogenated pyridinium ions bearing EWG or EDG substituents in position 5 and a model peptide. After measuring the *k*_2_ values, these have been plotted with the Hammett constants *σ_p_* for every substituent,^119^ giving a perfect linear correlation. The tunability of the S_N_Ar reaction can be easily followed with the Hammett plot as the negative charge resulting from the nucleophilic attack lies directly on the aromatic ring, enhancing the stabilizing or destabilizing electronic effect of the substituents. In fact, the magnitude of the slope of the correlation curve is moderate (*ρ* = 3.4), and the positive value accounts for the generation of a negative charge or the quenching of a positive charge, like in the case of pyridinium ions. As expected, the highest reaction rates were obtained with EWG substituents, whereas EDG substituents, *i.e.*, methoxy and amino, showed lower reactivity with reaction times of around 1 h at 37 °C.

**Figure 18:**
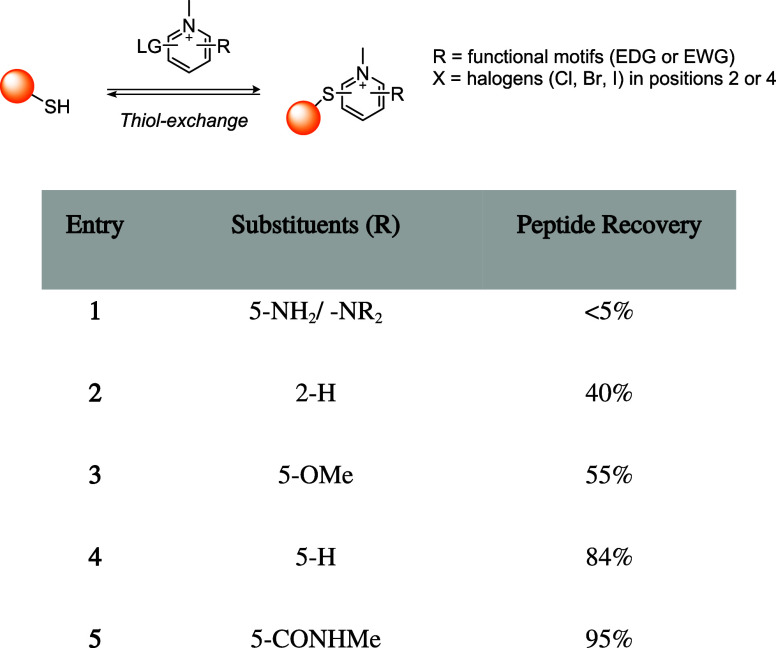
Reversible thiol bioconjugation by means of pyridinium ions and peptide recovery values.

The same results were obtained, as expected, for the cleavability of the thiol adduct in the presence of an additional thiol like GSH. In fact, the S_N_Ar reaction can be reversible in the presence of stronger nucleophiles that behave as a worse leaving group than the previously attached thiol (Scheme 33). The authors also highlighted that pyridinium-thiol adducts are quite stable to reducing agents unless thiol-based reductants are used, such as βME or DTT (TCEP is well tolerated). These compounds have been employed for different applications such as the conjugation of peptides, the stapling of peptides to mask or alter their functions, and the conjugation of BSA, for which we invite the reader to look up ref 118.

Another interesting class of linkers suitable for cysteine bioconjugation is the one of tetrazines, as reported by the group of Neumann.^120^ Successful reversible S_N_Ar was already reported by reacting symmetric 3,6-heteroatom bearing tetrazines with hydrogen sulfide, but no reaction was observed with biologically relevant thiols. For this reason, the group of Neumann turned its attention to more electron-deficient asymmetrical 3,6-disubstituted tetrazines bearing a methyl thioether as a leaving group. The authors studied the reaction of 2-(Boc-amino)­ethanethiol as a model nucleophile with *N*-Boc piperidinyl tetrazine derivative **190** in buffered aqueous solution (Scheme 34), obtaining 70% conversion determined via RP-HPLC.

**Scheme 34:**
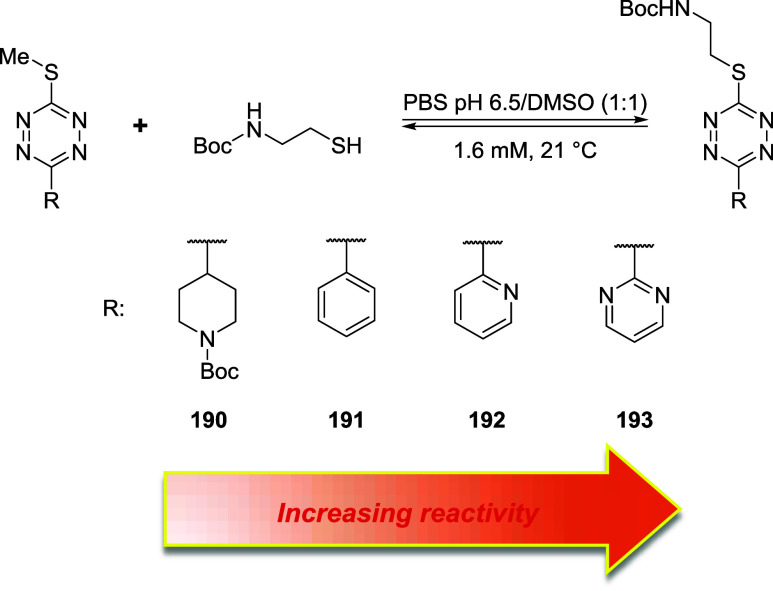
Conjugation of Substituted 3-Thiomethyl Tetrazines with 2-(Boc-amino)­ethanethiol

The authors then evaluated different parameters, *i.e.*, solvent, pH, and concentrations, highlighting that the reaction do not work in pure organic solvent without a base, suggesting that a mixture of organic solvent and aqueous solution is a good compromise for the reaction to proceed; as mentioned in the previous sections, pH is crucial as it increases the concentration of thiolate anion, highlighting that the reaction proceeds smoothly at pH values ranging from 4.5 and 8.5, above which tetrazine decomposition was observed (the best result was obtained with pH 6.5); high thiol concentration decreases the competition with methanethiol, which can be removed by the reaction medium by bubbling N_2_, shifting the equilibrium toward the formation of the product. After all of these evaluations, the authors highlighted that quantitative conversion can be obtained by bubbling the reaction mixture with N_2_, with equimolar amounts of tetrazine and thiol (200 *μ*M) at pH 6.5. Electronic effects on the conjugation reaction were evaluated by functionalizing the tetrazine ring with EDG or EWG substituents (**190–193**) (Scheme 34) and reacting them with a model peptide, resulting in higher reactivity for the more electron-poor derivatives **192** and **193**. The rate constants of this process were measured by exploiting the ability of tetrazine to quench fluorescence via Förster resonance energy transfer (FRET), monitoring the decay of the fluorescent signal upon treating derivatives **190–193** with a fluorescent thiol (*k*_2_: 2.6 M^–1^ s^–1^, 12.8 M^–1^ s^–1^, 20.1 M^–^ s^–1^, and 24.8 M^–1^ s^–1^, respectively). The authors also pointed out an interesting feature of these compounds, which can undergo inverse electron demand Diels–Alder reaction (IEDDA) to form a less electron-poor compound that is less prone to S_N_Ar than the parent tetrazine-thiol adduct. In fact, the authors demonstrated that treating **190** with a small peptide afforded a thiol adduct that underwent reversible S_N_Ar after the addition of DTT, in contrast with the result obtained in the presence of a bicyclononine compound, which yielded the corresponding stable pyridazinyl-thiol derivative.

The next two examples shed light on how the aromatic scaffold could influence the rate of the S_N_Ar reaction, more than the presence of electron-withdrawing substituents. In the first study, Li et al. explored the chemistry of several aromatic derivatives owning purine, pyrimidine, quinazoline, triazole pyrimidine, and pyrazolopyrimidine scaffolds.^121^ After benzoxazole sulfide was discovered to be the best leaving group for the purine scaffold, the authors screened different pH buffers and solvent systems, highlighting Tris buffer (pH 8.0)/DMSO (1% v/v) as the best combination to perform the reaction. To evaluate the importance of the aromatic scaffold, several benzoxazole sulfides bearing the above-mentioned aromatic moieties decorated with electron-withdrawing or electron-donating groups were designed and synthesized (Scheme 35).

**Scheme 35:**
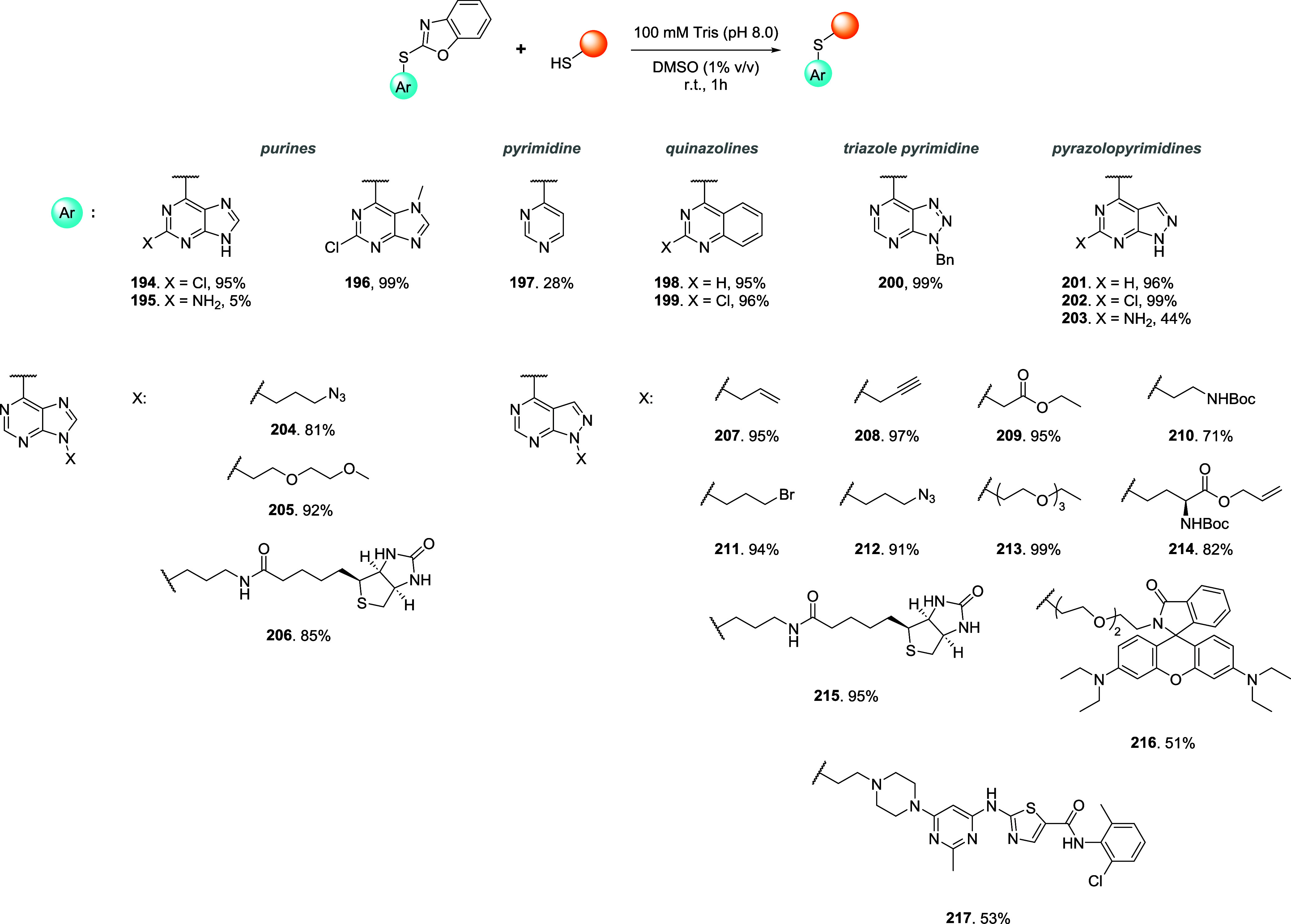
Synthesis of Benzoxazole Sulfides **194**–**217**, Featuring Purine, Pyrimidine, Quinazolines, Triazole Pyrimidine, and Pyrazolopyrimidine Scaffolds

As expected, the authors observed that electron-withdrawing groups accelerate S_N_Ar reaction with a model peptide (**194** vs **195**, **202** vs **203**). All derivatives reacted smoothly with the model peptides, but since purine and pyridazolopyrimidine-based benzoxazole sulfides were found to be more efficient, several derivatives containing these two scaffolds were synthesized, decorated with handles for biorthogonal transformations, or with recognition tags (**204–217**). Despite the absence of EWG or EDG substituents on the aromatic ring, pyrazolopyrimidine performed better than purine as a scaffold, highlighting that the nature of the scaffold is relevant in S_N_Ar reactions. The authors then evaluated the efficiency of derivative **201** toward the labeling of different peptides containing all nucleophilic naturally occurring amino acids. In any case, **201** smoothly labeled the cysteine residues in the selected peptides with no side labeling detected. Moreover, no reaction was observed for the peptide depleted of the cysteine residue, confirming the chemoselectivity of these linkers for thiols. To evaluate the stability of these conjugates, modified peptides were exposed to acidic, basic, and oxidizing media, resulting in low stability only in the presence of periodic acid solutions, resulting in complete oxidation in 24 h. The authors also observed that the cleavage of the thiol adduct is highly dependent on the nature of the aromatic moiety. In fact, treating the pyrimidine, triazole pyrimidine, pyridazolopyrimidine, and quinazoline-based thiol adducts with βME and—to a lesser extent—GSH resulted in regeneration of the parent peptide, except for the purine scaffold. Despite the increased reactivity caused by EWG substituents, GSH–promoted deconjugation was not as efficient as βME-promoted one due to the bulkiness of GSH. In the second study reported, Tang et al. describe the application of heteroaromatic azoline thioethers (HAT, **218**) for the reversible conjugation of cysteine (Scheme 36A).^122^ Unlike the previous examples, the thiol source is not regenerated by competitive S_N_Ar but by reduction of the probe. Moreover, degradation at pH 10.5 causes the detachment of the linker, leaving behind a dehydroalanine (DHA) amino acidic residue on the peptide or protein (**220**), giving the chance to perform further functionalization. The authors explored several aromatic scaffolds to investigate the feasibility and the rate of cysteine labeling without introducing any additional EWG substituents on the heterocycle. The reactivity of these probes was tuned by replacing the heteroatoms in the scaffold, changing the heterocyclic ring to azole, fusing additional aromatic rings to make heteroaromatic azoline thioethers, changing the oxidation state of the thioether moiety, and evaluating different degrees of methylation on the heterocyclic nitrogen atoms (Scheme 36B).

**Scheme 36:**
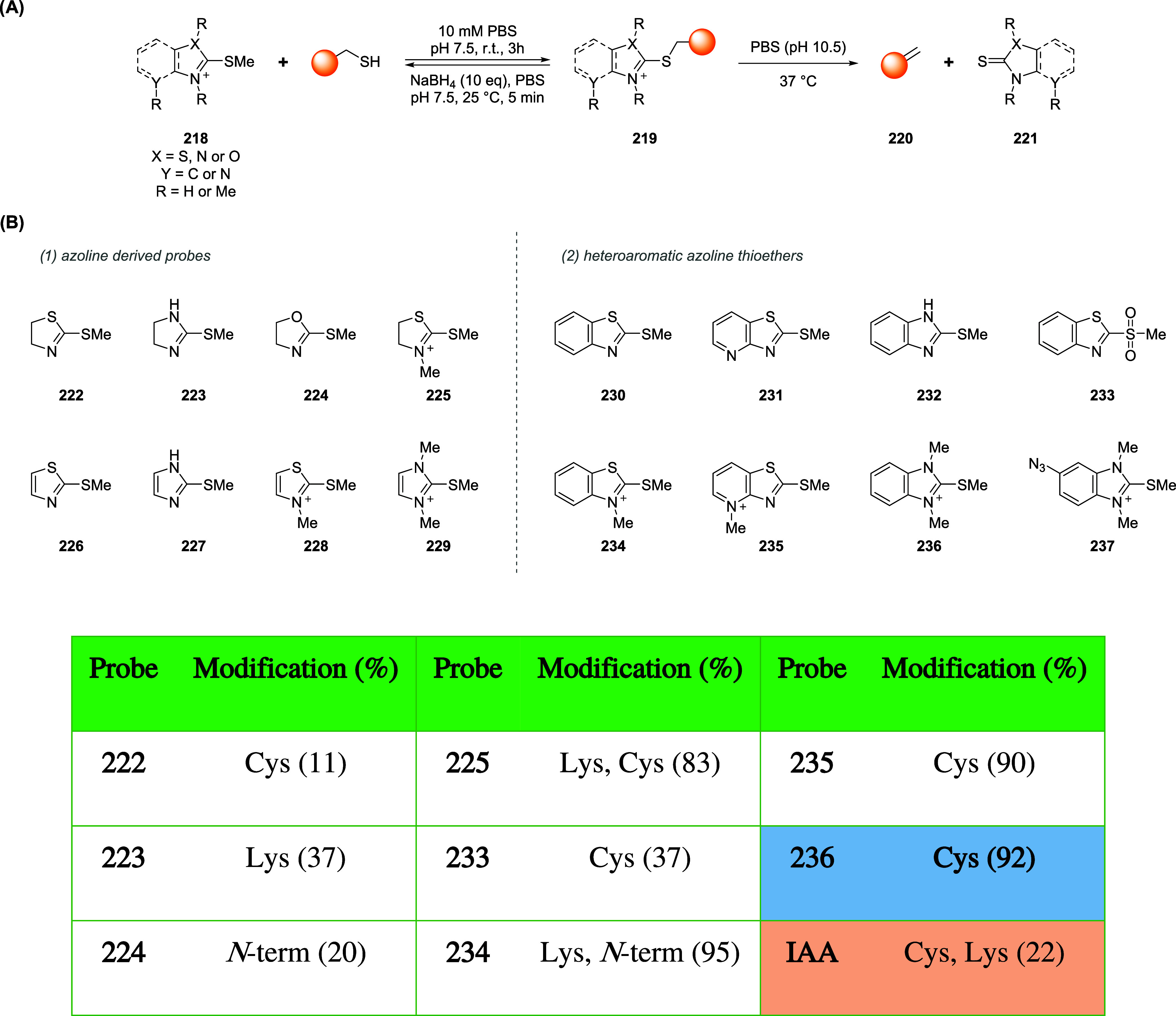
(A) Reversible Thiol Bioconjugation by Heteroaromatic Azoline Thioethers and Alternative Hydrolysis of the Thiol Adduct to Afford DHA-Containing Peptide or Protein. (B) Structures of azoline-derived thioethers and heteroaromatic azoline thioethers

The authors tested these derivatives toward a model peptide containing Cys, Lys, and free *N*-terminal as nucleophilic amino acid residues, and the results obtained revealed that changing the heteroatom modulates the chemoselectivity of the reaction, as shown for compounds **222–224**. Methylation of the nitrogen heteroatom of **222** yielded a derivatization with higher efficiency but low selectivity, as expected by the increased electrophilicity of **225**. However, in the case of linkers **226** and **227**, no conjugation was observed, even for their methylated forms. The authors also fused aromatic rings to compounds **222** and **223**, obtaining HAT probes **230–232** that feature low solubility in the employed PBS buffer, resulting in deprived reactivity. Oxidation of the thioether moiety of HAT **230** leads to the formation of water-soluble sulfone **233** capable of selective cysteine labeling. Methylation of the HAT probes yields electrophilic derivatives with enhanced reactivity toward cysteine conjugation, with **235** and **236** being the most efficient and selective compared with the well-known IAA linker. The authors evaluated the rate of cysteine modification by **236** and IAA, observing more than 80% of conjugation of a model peptide by **236** in 5 min, in contrast with the 30% of conversion obtained with IAA. The authors also compared the reactivity and stability of **236** and **233**, observing a greater rate constant (*k*_O_ = 236.77 M^–1^ s^–1^ vs *k*_L_ = 23.43 M^–1^ s^–1^) and higher water stability in the case of compound **236**. Finally, the authors compared probes **236** and its azido derivative, **237**, obtaining similar kinetics and stability, confirming that modulation of the scaffold is enough to obtain derivatives with predictable reactivity. It was also pointed out that HAT bioconjugates are stable at pH 3.5 (for 48 h at 25 and 40 °C), they showed low decomposition (10%) after 24 h at pH 7.5, and they did not react in 48 h in the presence of TCEP. To assess cysteine selectivity in protein bioconjugates, the authors tested all the active probes toward myoglobin (Mb) and used IAA for comparison. Since Mb lacks a cysteine residue, compounds **233** and **235–237** were unable to modify the protein, confirming the selectivity of these derivatives toward cysteine, in contrast to the remaining tested probe, which reacted with the lysine residues as highlighted with MS measurements after digestion. The same results were obtained with both native and reduced insulin (no free Cys vs 6 free Cys) in the presence of probes **235–237**, which reacted only with the reduced protein. In addition, to evaluate the possible regeneration of the protein, the *O*-insulin bioconjugate was treated with sodium borohydride, obtaining unmodified insulin chains A and B, as highlighted in LC-MS analyses. For further applications of HAT probes, *i.e.*, BSA bioconjugation, biotinylation via SPAAC (strain-promoted azide–alkyne cycloaddition), and umpolung of cysteine residues, we kindly invite the reader to look up ref 122.

## 6 Aliphatic Nucleophilic Substitutions

Aliphatic nucleophilic substitutions constitute a mechanistically distinct class of thiol-selective bioconjugation reactions that rely on direct nucleophilic attack by thiols on *sp* ^*3*^-hybridized electrophilic carbon centers bearing good leaving groups (Scheme 37).

**Scheme 37:**
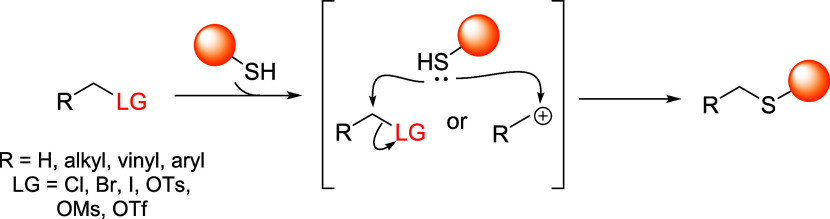
Aliphatic Nucleophilic Substitutions General Mechanism Representation

In contrast to 1,2-addition reactions, these reactions do not require conjugation or electron-deficient double bonds but instead depend on the leaving group and the lability of the electrophilic center. Importantly, the reversibility and modularity of these reactions can be triggered through modifications of the electrophilic site or triggered externally via light. Two major subtypes within this category are benzylic substitutions and α-haloamides, which differ in their electrophilic centers and their activation strategies but converge on a common mechanism of thiol-mediated substitution (Figure 19).

**Figure 19:**
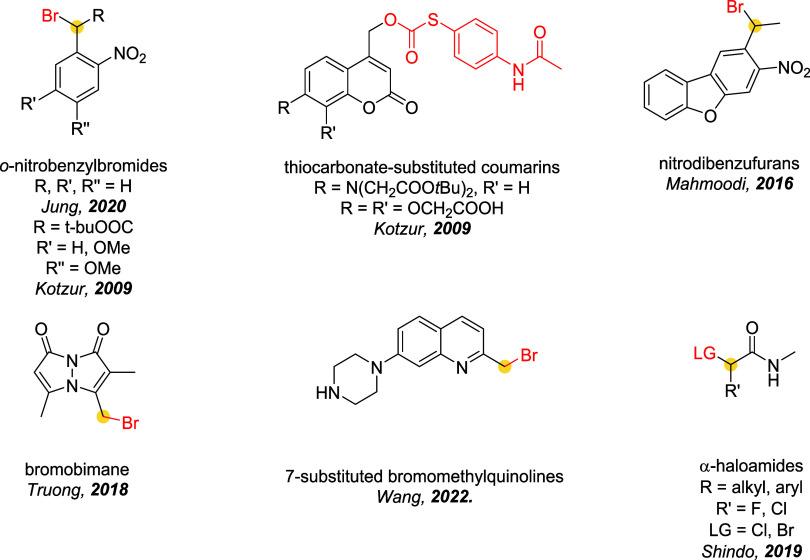
Different classes of thiol-selective scaffolds documented for reversible protein conjugation via an aliphatic nucleophilic substitution mechanism. Bioconjugation sites are marked with a yellow dot. Leaving groups are highlighted in red.

### 6.1 Benzylic Halides

Benzylic substitution-based thiol bioconjugation exploits the increased reactivity of benzylic carbons, which undergo nucleophilic substitution with thiolates, in some cases upon photoactivation. A relevant example of this bioconjugation strategy was reported by Hagen et al., who focused on the development of coumarin-based (**238**, **239**) and *o*-nitrobenzyl-based (**240**, **241**) systems capable of efficient thiol release upon light exposure (Figure 20).^123^

**Figure 20:**
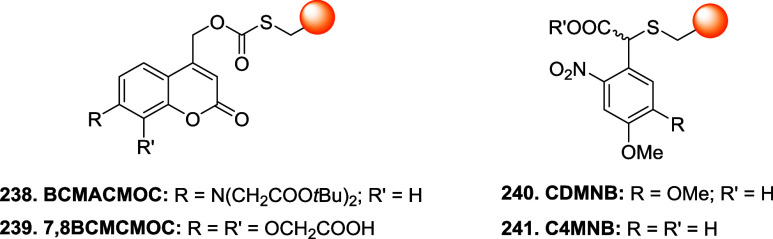
Two classes of photolabile protective groups (PPGs) caging a peptide.

The authors synthesized a series of Fmoc-Cys-OH derivatives bearing different PPGs: BCMACMOC (**238**), 7,8BCMCMOC (**238**) (coumarin-based), CDMNB (**240**), and C4MNB (**241**) (nitrobenzyl-based). In the case of the coumarin-based PPG, the scaffold was designed with polar groups (*e.g.*, carboxymethyl substituents) to enhance the water solubility and minimize aggregation. The removal of coumarin- and nitrobenzyl-derived thiol-protecting groups proceeds by distinct but related photochemical mechanisms that exploit the electronic properties of the chromophores to induce fragmentation of the C–S bond and liberation of the free cysteine residue. In the case of coumarinylmethyl thiocarbonates (**238**, **239**), irradiation in the long-wavelength absorption band around 430 nm promotes the chromophore to an excited singlet state that undergoes efficient π→π* excitation, producing a highly delocalized excited system. The sulfur leaves as a thiocarbonate, which is rapidly protonated in aqueous buffer to yield thiocarbonic acid, undergoing decarboxylation to give free thiol. The carbocation intermediate is quenched by solvent molecules, giving the corresponding coumarinyl alcohol (**242, 243**) as the stable byproduct.^123^ In some cases, this carbocation may undergo intramolecular rearrangement, leading to coumarin-3-yl thioethers, which account for the incomplete recovery of cysteine in certain derivatives (Scheme 38).

**Scheme 38:**
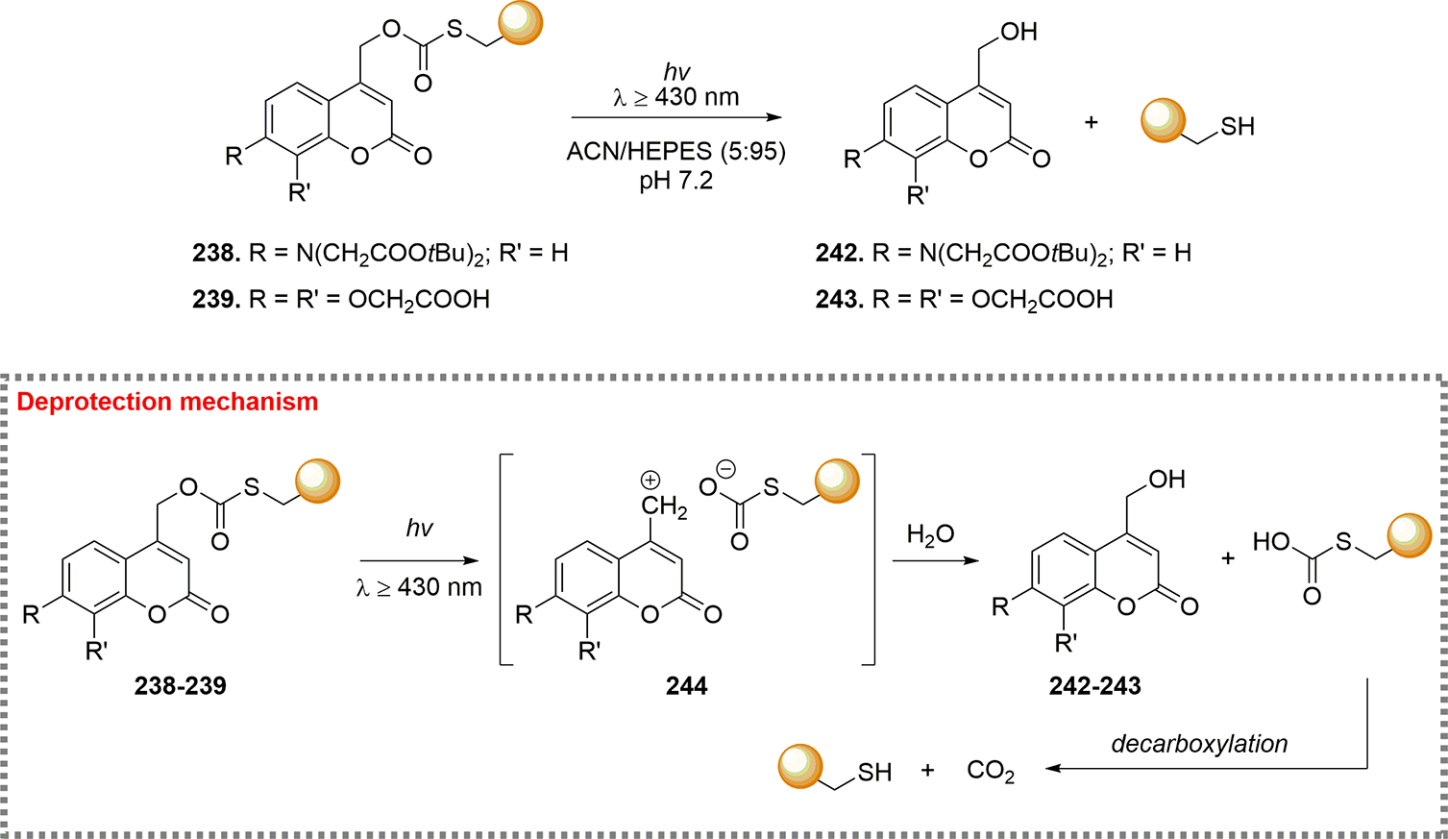
Photocleavage Mechanism of Coumarin-Protected Thiol Proceeds by Fragmentation of the C–S Bond and Liberation of the Free Cysteine Residue

On the other hand, the 2-nitrobenzyl group follows a different course upon excitation around 325–350 nm as it is converted into the *aci*-nitro derivative **247**; the latter is engaged in an intramolecular rearrangement affording nitroso derivatives **245** or **246**, thus releasing the unmodified thiol (Scheme 39).^124^

**Scheme 39:**
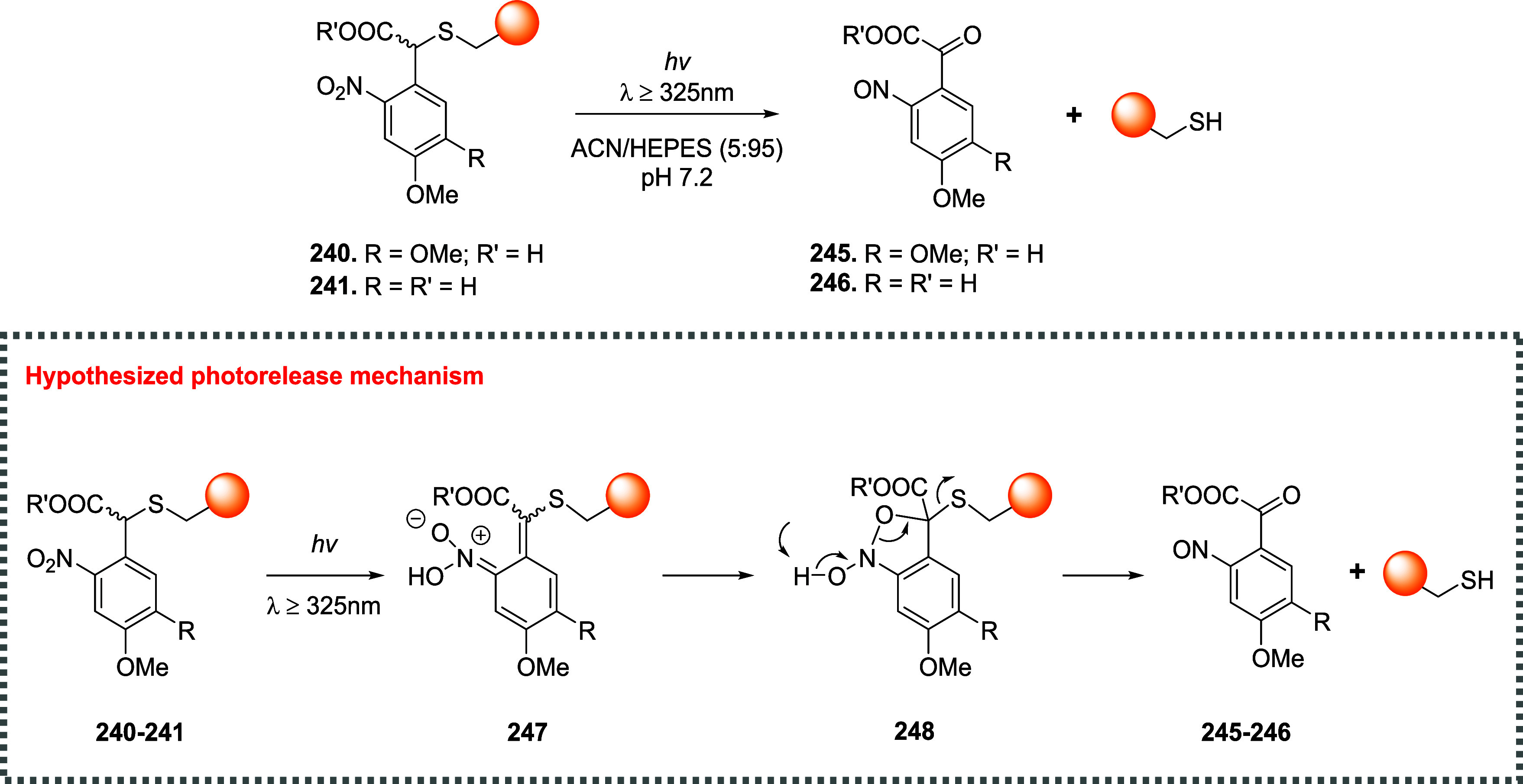
Photocleavage Mechanism of 2-Nitrobenzyl-Protected Thiol Proceeds by Fragmentation of the C–S Bond and Liberation of the Free Cysteine Residue

These derivatives exhibited distinct absorption maxima and photochemical quantum yields, permitting selective photolysis in mixtures. For instance, the extinction coefficient ratio between **238** and **239** at 402 nm exceeded 100, allowing the selective deprotection of one thiol over the other. These cleavable thiol cages were applied to the model peptide resact, a sperm-attractant peptide from *Arbacia punctulata* bearing two cysteine residues demonstrating the effective wavelength-controlled photocleavage of various s-protected *N*-Fmoc-cysteine mixtures. An innovative thiol-caging approach that relies on benzylic substitution was reported by Jung and co-workers, who developed thiol-substituted poly­(2-oxazoline)­s masked with a photoremovable protecting group, specifically a 2-nitrobenzyl moiety. The authors synthesized the oxazoline (called **NbMEtOxa** in the study), which was copolymerized with 2-ethyl-2-oxazoline (EtOxa) through a cationic ring-opening polymerization strategy. Upon UV irradiation (λ = 365 nm), the 2-nitrobenzyl group was cleaved, liberating reactive thiol groups that enabled in situ thiol–ene click cross-linking with tetraacrylate linkers, leading to the formation of covalently cross-linked polymer networks. This stepwise process represents an indicator of how benzylic substitution can be exploited for activation in soft-material engineering, particularly in light-triggered systems.^125^ In a similar study, Mahmoodi et al. reported a photoremovable protecting group based on nitrodibenzofuran (NDBF) for cysteine thiols, exploiting a benzylic substitution mechanism to generate stable thioether linkages amenable to controlled photolysis.^26^ At first, the authors explored coumarin-based cages such as brominated hydroxycoumarin (**249**) (Bhc) for thiol protection due to their strong one- and two-photon absorption properties. However, this study revealed that upon irradiation these groups frequently undergo photoisomerization to noncleavable thioethers rather than efficient uncaging, limiting their general applicability. Compared to Bhc, the NDBF cage showed a markedly reduced tendency toward photoisomerization, a common side reaction that leads to noncleavable thiol adducts via benzylic rearrangement (Scheme 40).

**Scheme 40:**
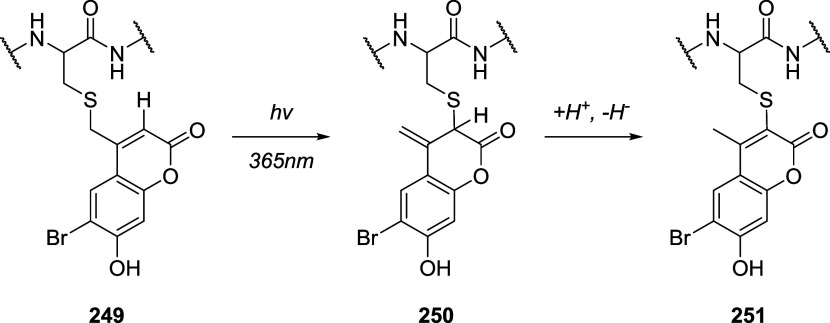
Bhc (249) Photoisomerization Mechanism

To overcome these drawbacks, Mahmoodi et al. investigated *o*-nitrobenzyl derivatives, and in particular NDBF. The NDBF group was installed *via* nucleophilic substitution of the benzylic bromide by the thiol group of Fmoc-Cys-OMe, affording a C–S bond at the benzylic position (**252** in Scheme 41).

**Scheme 41:**
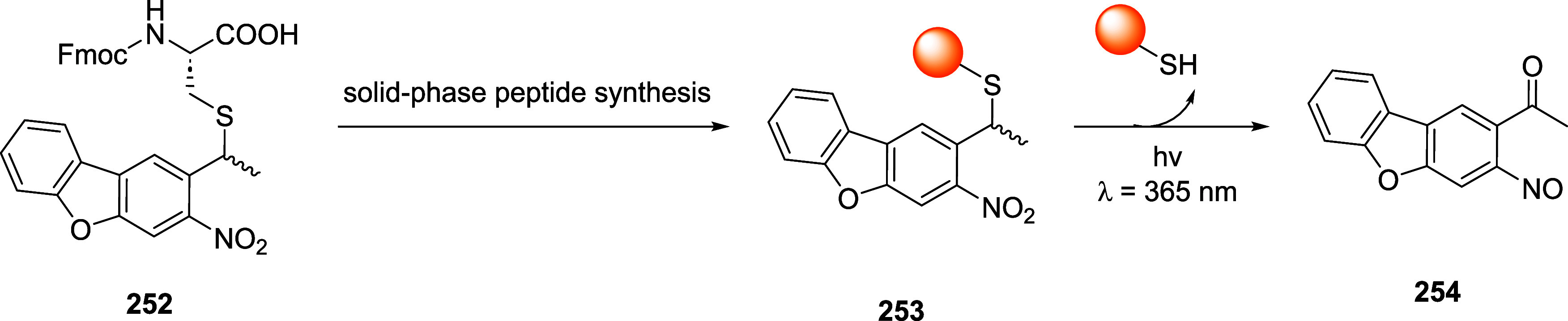
Solid-Phase Synthesis of the NDBF-Caged Peptide **253** and Consequential Photolysis at 365 nm

This strategy led to the formation of the caged cysteine and consequential peptide (**253**) through solid-phase synthesis without detectable side products, and subsequent deprotection could be induced with both one-photon (365 nm) and two-photon (800 nm) excitation. Mechanistically, photolysis proceeds through photoexcitation of the chromophore, followed by intramolecular electron transfer and heterolytic cleavage of the benzylic C–S bond, liberating the free thiol, similar to the previously reported *o*-nitrobenzyl thiol-photorelease mechanism. The practical utility of this system was demonstrated in both enzymatic and cellular contexts. A K-Ras4B-derived peptide bearing an NDBF-caged cysteine was efficiently uncaged under UV light and underwent site-specific enzymatic farnesylation by farnesyltransferase *in vitro*. Furthermore, a fluorescent cell-penetrating peptide equipped with the same protecting group was applied to SKOV3 ovarian carcinoma cells. Upon localized irradiation, the intracellular redistribution of the probe from cytosol/Golgi to the plasma membrane confirmed successful intracellular uncaging and subsequent palmitoylation by endogenous enzymes.

In another relevant example, Truong et al. reported a novel photocleavable bimane-derived linker capable of mediating reversible, traceless thiol exchange reactions under biologically compatible conditions.^126^ The authors designed a benzylic thioether architecture in which a bimane fluorophore is conjugated to a thiol through a benzylic carbon–sulfur bond. Upon visible-light irradiation (λ = 420 nm), the bimane core (**255**) undergoes photoinduced heterolytic cleavage at the benzylic C–S bond, affording a benzylic carbocation intermediate **256** and the unmodified thiol, that in the presence of an electrophile generates a new tioether (**259**) (Scheme 42).

**Scheme 42:**
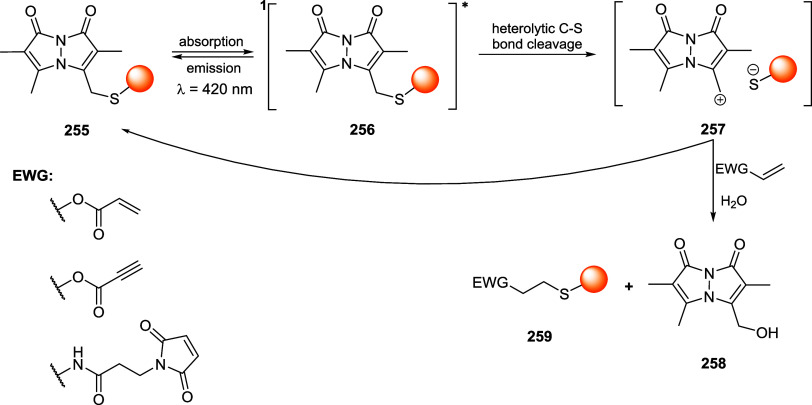
Photoinduced Heterolytic Cleavage at the Benzylic C–S Bond, Affording the Benzylic Carbocation Intermediate **257** and the Unmodified Thiol that can be Scavenged by EWG-Substituted Alkene

Carbocation **257** is highly reactive and can be rapidly intercepted by a second thiol nucleophile present in solution, leading to a new benzylic thioether through the completion of a thiol exchange reaction. The reaction is reversible and traceless, as the departing thiol is released unmodified, and no linker residues are transferred. Mechanistic studies ruled out radical intermediates by showing no effect of radical scavengers on photoreaction efficiency, and mass spectrometric analysis confirmed the exclusive formation of exchange products without fragmentation byproducts. Experimentally, the system was validated through a series of reactions involving bimane–peptide conjugates exposed to alternative thiol-containing nucleophiles (*e.g.*, glutathione, benzyl mercaptan, and cysteine derivatives). In a model reaction, a bimane-conjugated peptide was irradiated in the presence of βME, resulting in rapid cleavage of the initial C–S bond and formation of the new conjugate as confirmed by LC-MS. The extent of product formation was quantified by HPLC, and the reaction progress was monitored in real-time via the intrinsic fluorescence of the bimane chromophore. Importantly, the authors demonstrated that this thiol exchange process could be performed iteratively, with multiple sequential substitutions enabled by successive irradiation and the addition of alternative thiols. In one such experiment, a bimane conjugate bearing a model peptide was subjected to light-mediated substitution first with benzyl thiol and then with glutathione, cleanly affording the corresponding thioether conjugates in sequence without detectable side reactions.

Wang et al. expanded the concept by developing a quinoline-based photolabile protection system optimized for efficient, light-triggered thiol substitution.^127^ Specifically, they designed a photocage system based on a 7-piperazynil-quinoline scaffold that, upon irradiation at 365 nm, releases a reactive benzylic electrophile capable of undergoing rapid and site-specific substitution by cysteine thiolates. The core innovation resided in the use of the quinoline unit, which imparts water solubility and pH-responsiveness, combined with a photolabile linker that cleaves cleanly upon light activation to expose the benzylic site. In their experimental setup, the authors synthesized quinoline-caged precursor **260** (Scheme 43) and demonstrated that the uncaging reaction was highly efficient in an aqueous buffer, as confirmed by HPLC, UV–Vis spectroscopy, and mass spectrometry. They then applied this chemistry to site-specific bioconjugation of model peptides and proteins bearing terminal cysteine residues. Notably, the reaction was shown to proceed under mild conditions with excellent control over conjugation timing through light exposure (Scheme 43).

**Scheme 43:**
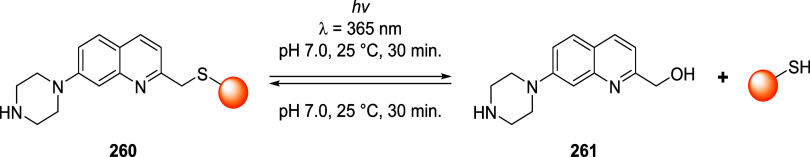
Quinoline-Caged Peptide and Subsequent Uncaging Reaction in an Aqueous Buffer

To validate biological applicability, the system was used to assemble dimeric and multimeric protein complexes in a stepwise, photoregulated manner. Gel electrophoresis and size exclusion chromatography confirmed the structural integrity and formation of the desired conjugates. The reversible nature of the thiol addition was not exploited in this system, but the modular design suggests that it could be adapted to include cleavable linkers if desired. Overall, their work represents a robust example of light-gated benzylic substitution suitable for orthogonal and spatiotemporally controlled protein engineering applications.

### 6.2 α-Haloamides

In a comprehensive study by Shindo and colleagues, α-chlorofluoroacetamide (CFA) was introduced as a novel electrophilic candidate for chemoselective, and partially reversible bioconjugation with cysteine residues in proteins.^128^ The choice of the CFA linker was driven by comparative reactivity assays showing that α-chloroacetamide-based linkers reacted too rapidly (*t*_1/2_ ≈ 0.04 h) and α-fluoroacetamides were too inert (*t*_1/2_ ≈ 42 h), while CFA displayed an intermediate reactivity (*t*_1/2_ ≈ 4.4 h) that allowed selective cysteine modification without excessive off-target labeling. Importantly, CFA adducts were found to undergo slow, environment-dependent hydrolysis, showing partial reversibility in solvent-exposed sites while remaining stable in the buried ATP-binding pocket of EGFR. Mechanistically, CFA reacts with cysteine thiols through a classical bimolecular nucleophilic substitution (S_N_2) pathway, whereby the thiolate attacks the α-carbon and displaces the chloride, forming a thioether bond while retaining the α-fluorine. The presence of fluorine was shown to fine-tune the electrophilicity of the center, striking a balance between sufficient reactivity toward target cysteines and reduced off-target alkylation under physiological conditions. Notably, the study demonstrated that CFA-based labeling is highly sensitive to the microenvironment of the target cysteine. When bound to cysteines located in solvent-exposed regions, such as in nonstructured protein domains, the fluoroacetamide-thiol adduct was observed to undergo hydrolysis under neutral aqueous conditions (pH 7.4, 37 °C), regenerating the free thiol and releasing a hydrated glyoxamide. This hydrolysis follows a proposed mechanism involving nucleophilic attack of water on the carbon adjacent to sulfur (**264**), facilitated by the electron-withdrawing nature of the fluorine and amide carbonyl (Scheme 44).

**Scheme 44:**
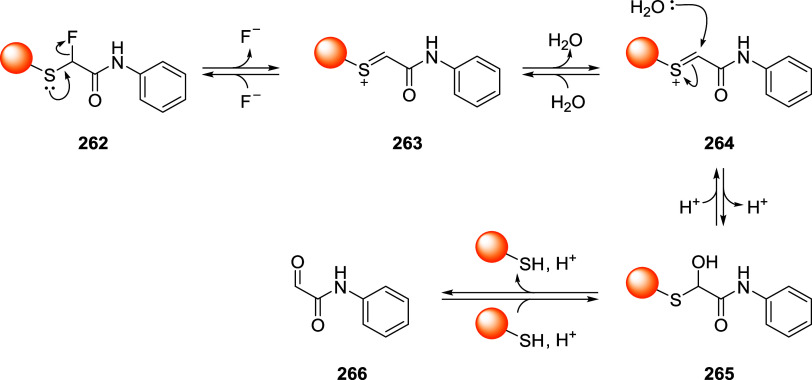
Fluoroacetamide-Thiol Adduct (**262**) Hydrolysis under Neutral Aqueous Conditions (pH 7.4, 37 °C), Regenerating the Free Thiol and Releasing a Hydrated Glyoxamide (**266**)

In contrast, when the CFA group reacted with cysteines buried within hydrophobic or sterically protected pockets, such as Cys797 in the ATP-binding site of EGFR, the thioether linkage exhibited high stability, resisting hydrolysis, even after prolonged incubation. This context-dependent reversibility allowed for a unique ″selective permanence″ whereby productive, high-affinity protein–ligand interactions yielded durable conjugates, while nonspecific or weak interactions could be erased over time through spontaneous hydrolysis. To explore this phenomenon, the authors synthesized a series of CFA-containing quinazoline derivatives targeting EGFR and systematically varied the linker structure between the CFA moiety and the kinase-targeting scaffold. They found that subtle structural changes, such as the inclusion of a *d* -proline linker, had profound effects on both the reactivity and the selectivity of the conjugation. In cellular assays, a CFA–quinazoline probe bearing a *d* -proline linker exhibited strong labeling of EGFR with minimal proteome-wide off-target reactivity, even at high micromolar concentrations. Stable Isotope Labeling by/with Amino acids in Cell culture (SILAC) quantitative proteomics further confirmed the narrow spectrum of CFA targets compared to conventional Michael acceptors such as acrylamides and 4-dimethylaminocrotonamide (DMAC). Importantly, in washout experiments using live cells, the EGFR–fluoroacetamide adduct persisted while off-target protein modifications gradually disappeared over time, supporting the concept of a hydrolysis-driven selective process. The mechanistic reversibility under aqueous conditions provides a built-in safety feature, potentially reducing long-term off-target effects.

## 7 Thiol–Disulfide Exchange Reactions

Another relevant class of reversible reaction in bioconjugation chemistry is represented by the thiol–disulfide exchange, a biologically relevant process that regulates the folding of proteins,^129^ the activity of enzymes that contain a cysteine in their catalytic site,^130^ and the cleavage of DNA by calichemicin and esperamicin.^131^ The mechanism involved in this type of bioconjugation consists of the nucleophilic attack of a thiolate anion on a disulfide bond, producing a new disulfide and a second thiolate anion (Scheme 45).

**Scheme 45:**
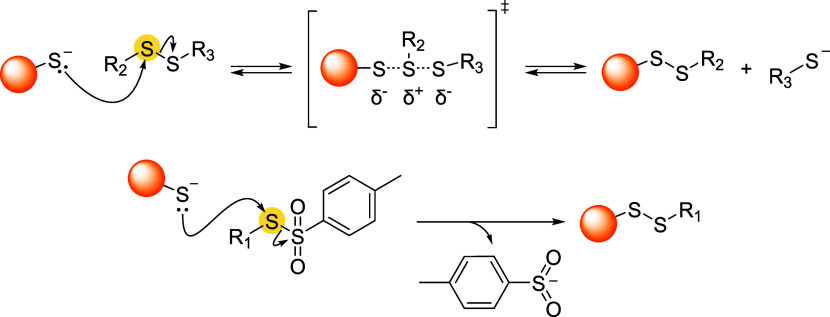
Generic Reaction Mechanism for Thiol Bioconjugation on Disulfides and Sulfonothioates by Means of Thiol/Disulfide Exchange Reaction

This process can be influenced by the redox environment, thiol p*K*_a_, and steric/electronic effects on the disulfide; specifically, the exchange is favored under reducing conditions, while the oxidative conditions (such as in the extracellular environment) confer greater stability to disulfide linkages. From a mechanistic point of view, the reaction proceeds through an S_N_2 mechanism, and the kinetics depends on the concentration of the thiolate anion. Moreover, the basicity and nucleophilicity of the thiolate are crucial for this reaction, as well as the pH of the medium. Hence, a thiolate with high p*K*_a_ values (*e.g.*, 9.0) is indeed a stronger nucleophile than a thiolate with a p*K*_a_ value of 4.0. However, proton dissociation of low p*K*_a_ thiols is favored at physiological pH, making thiolates the more reactive species in physiological conditions.^132^ In this section, the application of the thiol/disulfide exchange reaction in bioconjugation chemistry will be discussed. Various types of linkers (Figure 21) and the mechanistic aspects of the deconjugation processes will be analyzed, including the conditions that facilitate these reactions and the implications for practical applications in bioconjugation chemistry.

**Figure 21:**
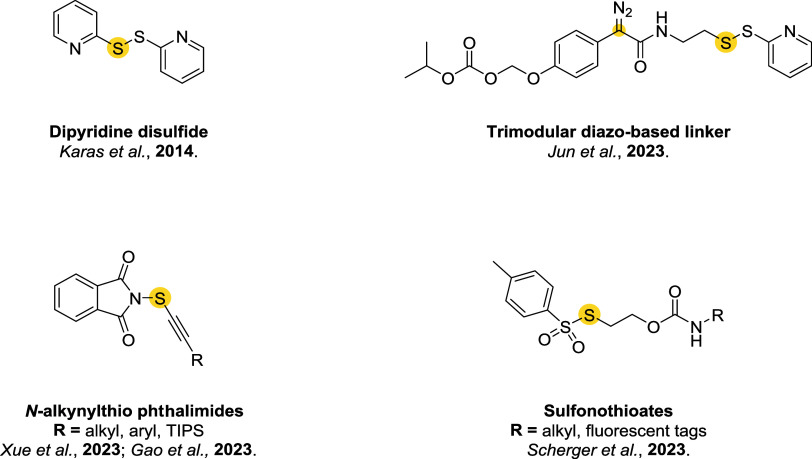
Different classes of thiol-selective scaffolds for reversible protein conjugation via a thiol/disulfide reaction. Bioconjugation sites are marked with a yellow dot.

The first example of this reaction was reported by Karas and colleagues, who combined photocleavable thiol protection with thiol/disulfide exchange to achieve disulfide bond formation in peptides by using 2-nitroveratryl bromide and dipyridine disulfide as protecting groups in solid-phase peptide synthesis (SPPS).^133^ The choice of the 2-nitroveratryl (*ortho*-nitroveratryl, *o*Nv) moiety as a protecting group was motivated by its high photolytic efficiency at 350 nm, essential to achieve a minimal degradation of sensitive amino acids such as tryptophan and tyrosine, which would be degraded at shorter wavelengths, like those employed for the cleavage of the more common 2-nitrobenzyl group. In this context, the authors exploited *o*Nv as a photocleavable protecting group of thiols in the synthesis of different peptides, such as oxytocin, α-conotoxin ImI, and human insulin. The synthesis of the target peptides proceeded through an iterative protection and deprotection strategy, allowing the authors to evaluate the stability of the *o*Nv scaffold in acidic media. As an example, the preparation of oxytocin is shown in Scheme 46. After selective deprotection of the *tert-*butyl group from the first cysteine residue and introduction of the *o*Nv group (compound **267**), the Mmt-protected cysteine was deprotected with a strong acid (triflic acid, TFMSA) and then protected with dipyridine disulfide, affording the (S-*o*Nv, S-Pyr)-protected peptide **268**. Photolysis of the *o*Nv protecting group by irradiation at 360 nm yielded a free cysteine thiolate (**269**), which rapidly engages in a thiol/disulfide exchange reaction with the disulfide group of the S-Pyr protected cysteine, affording the target peptide oxytocin (**270**).

**Scheme 46:**
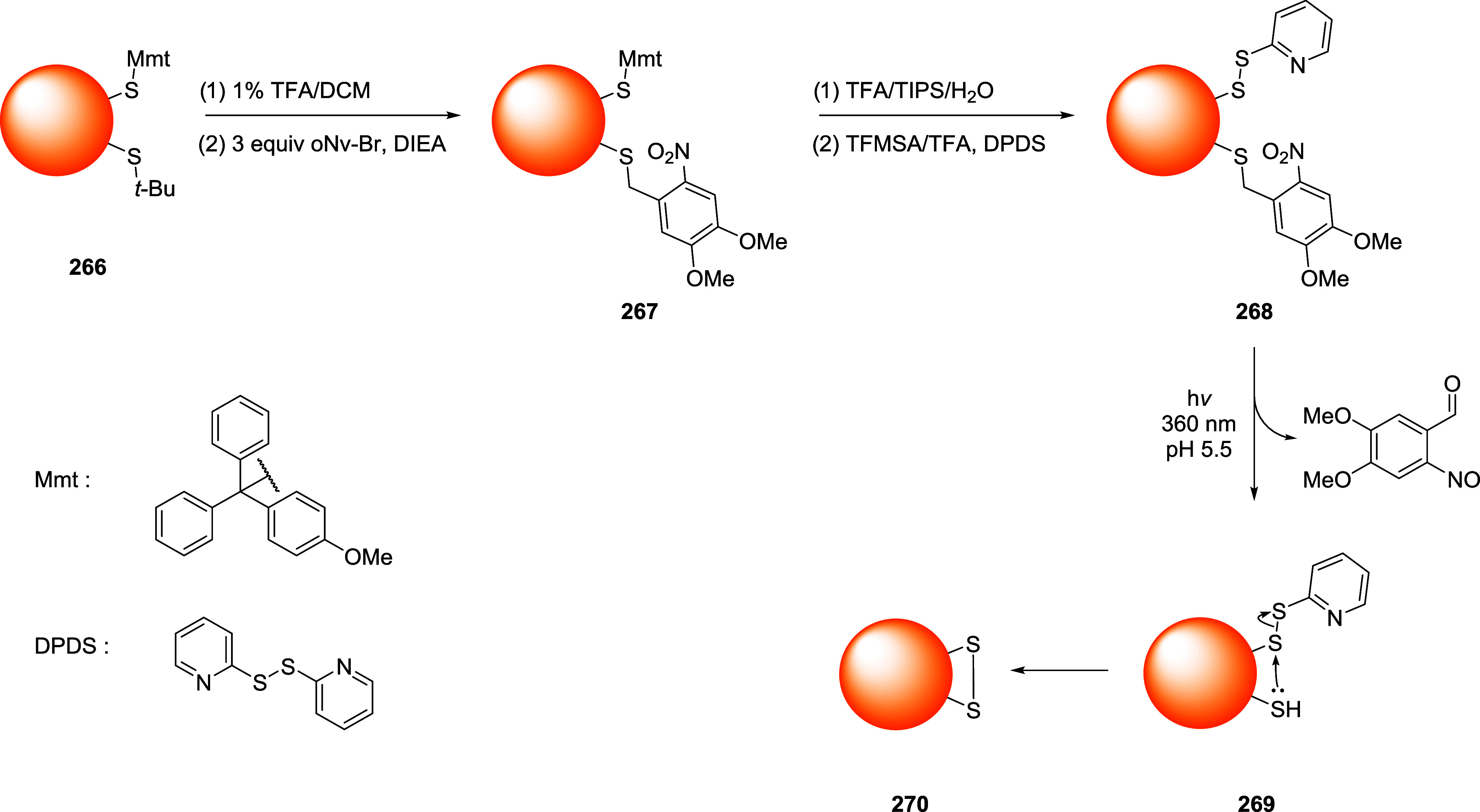
Use of 2-Nitroveratryl as a Photocleavable Thiol-Protecting Group for Directed Disulfide Bond Formation in the Chemical Synthesis of Oxytocin

In the case of α-conotoxin ImI and human insulin, the authors introduced S-*o*Nv cysteine during SPPS, evaluating the possibility of epimerization at the cysteine residue. After testing different conditions, the combination of DIC/HOBt resulted the best in minimizing racemization of the α-carbon to <0.5% at 25 °C, in contrast with HATU that gave 4% and 19% at 25 and 60 °C, respectively.

Jun and co-workers developed a diazo-based reagent that incorporates a thiol–disulfide exchange group into a modular system for the reversible modification and delivery of proteins.^134^ The principle was to merge three distinct reactive sites into a single molecule (**271**) to achieve the conjugation and the release of a biologically relevant cargo: a diazo group enables chemoselective esterification of protein carboxylates, a pyridyl disulfide moiety allows thiol-mediated functionalization, and an alkyloxycarbonyloxymethyl (AOCOM) group to provide traceless cleavage (Scheme 47).

**Scheme 47:**
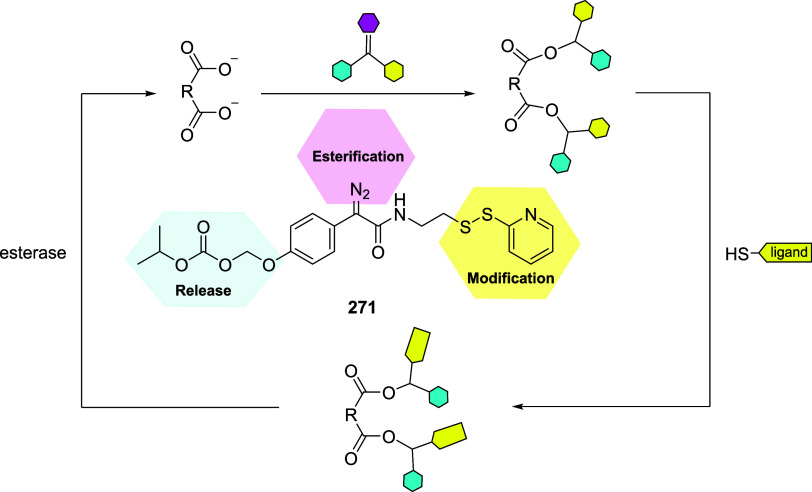
Conjugation Strategy for the Labeling of Proteins through the Trifunctional Compound **271**

The authors evaluated two bioconjugation strategies involving both cysteine-containing and cysteine-lacking proteins. In the first case, the pyridinyl disulfide moiety was reacted with a thiol source prior to the conjugation of the target protein, while in the second case, the target protein can be conjugated directly to the diazo moiety due to the lack of cysteine residues that could account for possible cross-conjugation. To validate these strategies for the delivery of proteins into mammalian cells, compound **271** was conjugated through the diazo moiety with human cytochrome *c* (Cyt *c*, a small cationic protein with no reactive cysteine residues) and with GFP (a large anionic protein with two cysteine residues), whereas it was modified on the disulfide site with several thiols such as 1-(2-mercaptoethyl)­guanidine (HS-guan), cyclic cell-penetrating peptide (CPP) deca-arginine (HS-cR_10_) and linear transactivator of transcription peptide (TAT) (HS-TAT), as well as a polysaccharide [HS-dextran­(110 K)] (Scheme 48).

**Scheme 48:**
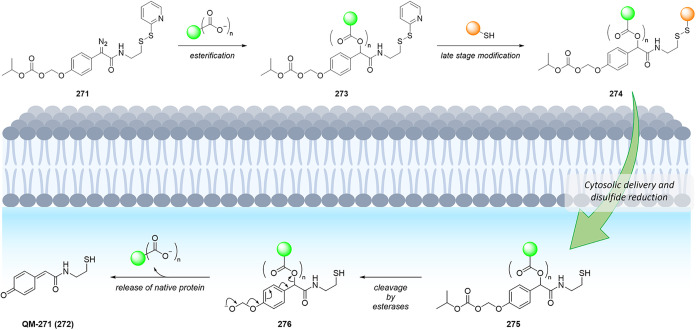
Mechanism of Delivery and Release for Proteins by Compound **271**

In all the cases, the extent of modification was confirmed by mass spectrometry underlining the selectivity of diazo moiety in **271**, which does not react with thiolates, amines, guanidines or *C*-terminal carboxylic groups, like in the case of HS-TAT. The reversibility of the system was then evaluated by exploiting the self-immolative nature of the AOCOM group in the presence of pig liver esterase (PLE), which hydrolyzes the carbonate moiety, regenerating the native protein and forming a quinone methide (QM-271) (**272**) byproduct upon elimination of formaldehyde. For these studies, model compound **277** was synthesized by esterifying **271** with *O*-(2-methoxyethyl)-glycolic acid and it was incubated with PLE in the presence of GSH (Figure 22)

**Figure 22:**
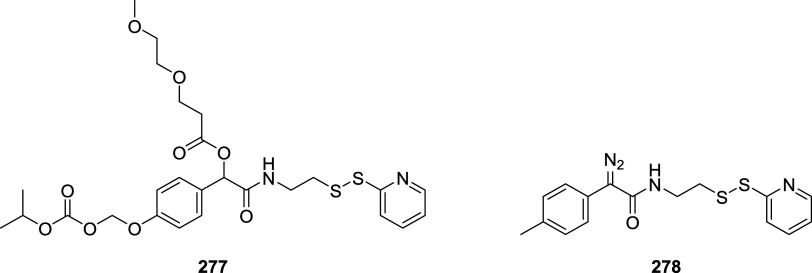
Structure of model compounds **277** and **278** designed for protein release studies.

MS analyses showed the presence of quinone methide adducts QM-**271**-OH, QM-**271**-GSH, and QM-**271**-pyS as proof-of concept for AOCOM-dependent hydrolysis and QM formation. The same experiment was also carried out with **278**, which showed slower release of labels, confirming the importance of the AOCOM group. GFP-**271**-cR_10_ and fluorescein-labeled Cyt *c*-**271**-cR_10_ conjugates, as well as the corresponding derivatives with **278**, were incubated with HeLa and M21 melanoma cell lines and analyzed by live-cell epifluorescence imaging, showing good internalization of the bioconjugates, in contrast with the unmodified proteins. Finally, a cell viability assay was carried out to assess the cytotoxicity of Cyt *c*-**271**-cR_10_ (Cyt *c* is a protein that induces apoptosis) to M21 cells, comparing its activity in the presence of cytosolic esterases with the Cyt *c*–cR_10_ conjugate prepared via irreversible lysine amidation. Despite the high cytosolic uptake of both the reversible and irreversible conjugates, only the former showed a dose-dependent toxicity, confirming the advantage and the efficiency of the trifunctional linker for both internalization and release of biologically relevant cargos.

Another example of this bioconjugation strategy was described by Xue and co-workers, who reported the use of *N*-alkynylthio phthalimides as efficient electrophilic sulfur-transfer reagents for the direct conversion of thiols into disulfides under mild, acid-catalyzed conditions (Scheme 49).^135^ The authors reported that 10% mol of trifluoroacetic acid is necessary to catalyze the reaction, enhancing the electrophilicity of phthalimide-masked alkynylthiols (**279**). Hence, optimization of the reaction has been carried out both in the presence and in the absence of TFA, resulting in 84% conversion within 1 min when the acid catalyst was employed. Then, a huge variety of thiols were employed for the subsequent thiol/disulfide exchange reaction—only a few examples are reported in this review—ranging from primary to tertiary thiols (**281–290**), cysteine-containing peptides (**291–295**), and thiol-containing drugs (**296–298**), obtaining alkynyl disulfides in good to excellent yields, rendering *N*-alkynylthio phtalimides an efficient and useful platform for bioconjugation (Scheme 49).

**Scheme 49:**
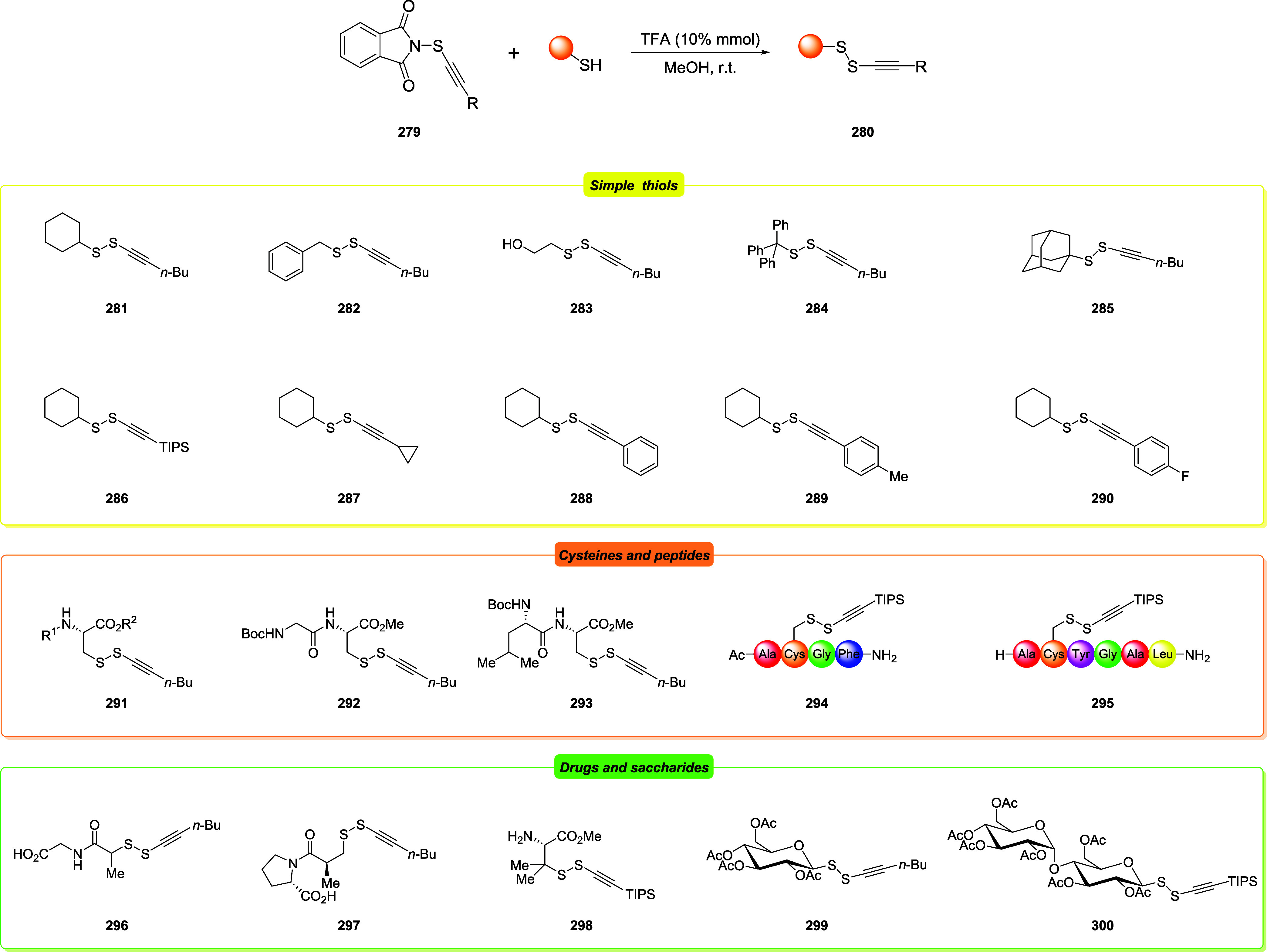
Synthesis of Alkynyl Disulfides **281–300** via Conjugation of *N*-Alkynylthio Phthalimides

Like in the previous examples, the reversibility of bioconjugation is achieved through thiol/disulfide exchange upon reaction with an external thiol, as GSH. The alkynyl disulfides still bear an alkyne moiety, which could be exploited as a handle for subsequent bio-orthogonal transformations, like click-type ligations, simultaneously preserving the disulfide bond. In fact, the authors also reported a protocol for metal-catalyzed azide–alkyne cycloaddition (AAC) by means of *N*-alkynylthio phtalimides. Unlike common AACs, in this case, copper is not suitable for catalyzing the reaction, leading to the decomposition of the starting materials. The authors overcame the problem by employing ruthenium and iridium as metal catalysts, which afforded *N*-triazolylthio phtalimides in moderate to good yields. Although thiols are well-known to poison metal catalysts, in the case of Ir-AAC the interaction between sulfur and iridium did not affect the reaction, allowing the authors to develop a one-pot protocol for this functionalization. However, in this procedure, adamantyl and *tert*-butyl thiols are the most tolerated, peptides are well tolerated with yields ranging around 50–65%, whereas primary thiols afford an undesired disulfide derived from the homocoupling as the major product.

The one-pot procedure was also employed for the modification of trifunctional molecules (**301**), featuring three independent reaction sites (Scheme 50).^136^

**Scheme 50:**
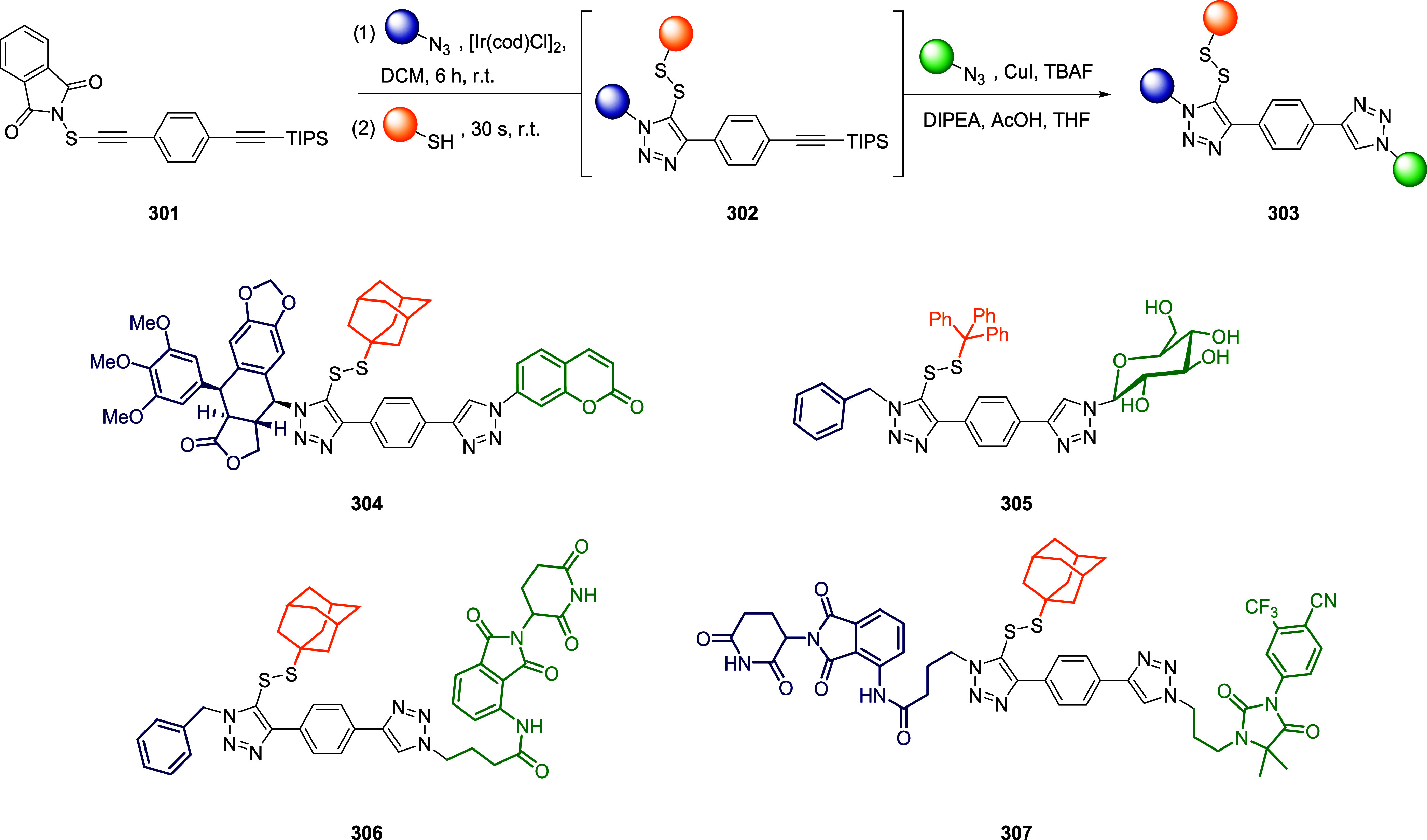
Synthetic Strategy for the Conjugation of Trifunctional Compound **301**

The authors exploited the reactivity of the different sites in *N*-alkynylthio phtalimides to achieve selective modification, by introducing the first triazole ring by IrAAC, selective for the alkynyl moiety proximal to the sulfur atom; successively, after the thiol/disulfide exchange (**302**), the second click reaction was performed in standard conditions using CuI as catalyst, obtaining compound **303**. This protocol rendered possible the conjugation of anticancer drug podophyllotoxin with fluorescent coumarin in compound **304**. It is also possible to exploit the adamantyl moiety in compounds **304**, **306**, and **307** to mimic the partial unfolding of a target protein, leading to its proteasomal degradation.^137,138^ This strategy was used by the authors to synthesize a proteolysis-targeting chimera (PROTAC) (**307**) decorated with a cereblon (CBRN) ligand and an androgen receptor (AR) ligand, considering that AR-dependent transcription is a major drive for prostate tumor cell proliferation.^139^

The last example of reversible conjugation via thiol/disulfide exchange is represented by the study of Scherger et al., who developed sulfonothioate-based self-immolative linkers (SIL) to achieve site-specific and reversible modification of nanobodies for the delivery of pharmaceutically relevant cargos.^140^ This strategy was applied on nanobodies engineered with a single *C*-terminal cysteine, avoiding any alteration on the secondary structure by leaving the internal disulfide linkages untouched and ensuring a site-specific conjugation to an accessible amino acidic residue. Since monomeric nanobodies tend to dimerize over time during storage, and conjugation occurs via a thiol/disulfide exchange, the authors developed a one-pot protocol that avoids the use of thiolated reducing agents (Scheme 51). The terminal cysteine was first reduced to its thiol form using TCEP, then the unreacted reducing agent was quenched in a Staudinger reaction using an excess of 4-azidobenzoic acid (ABA).^141^ The reduced nanobody (*e.g.*, α-MMR Nb) (**312**) was conjugated to the sulfonothioate **309** (see Scheme 51), decorated with the SIL for traceless release and with tetramethylrhodamine (TAMRA)-cadaverine (**310**) for imaging analyses, obtaining α-MMR Nb-SIL-TAMRA bioconjugate **313**. Reduction of the disulfide bond by thiols (*e.g.*, GSH or βME) releases the conjugated nanobody through thiol/disulfide exchange and triggers the traceless cleavage of **310** by self-immolation.

**Scheme 51:**
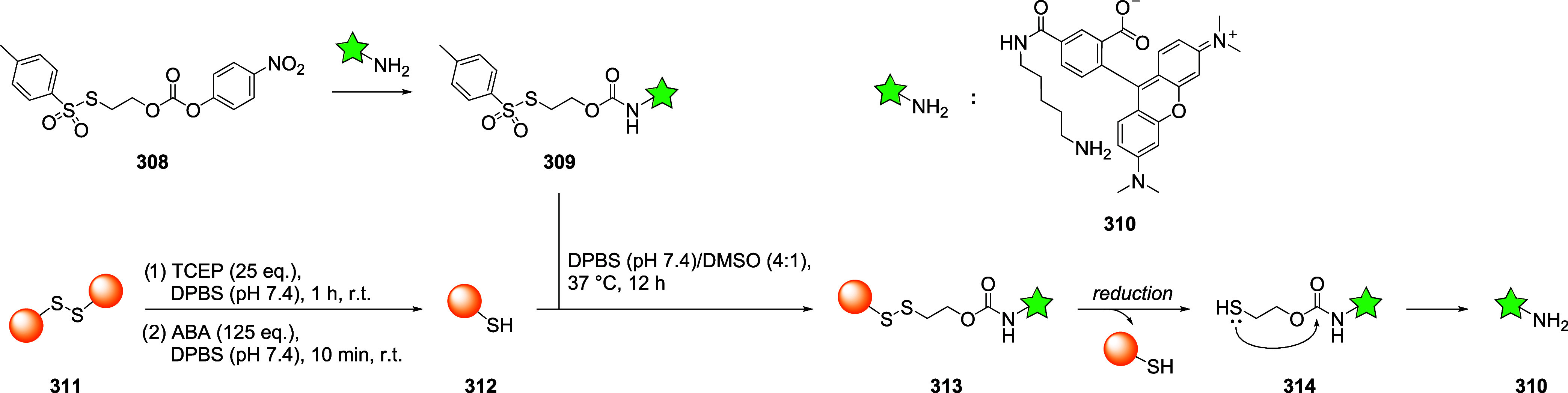
Reversible Conjugation of α-MMR Nb **312** with Sulfonothioate **310**

To verify the activity of the modified nanobodies, the authors investigated the internalization of **313** inside Chinese Hamster Ovary cells genetically modified to express MMR receptor (CHO^MMR+^). Flow cytometry analysis showed nanobody uptake inside the cells, in contrast to the negative control (CHO^MMR–^), confirming that bioconjugation does not affect the activity of the nanobody. This protocol was developed for fluorescent probes (like **310**), different nanobodies, and different bioactive small molecules (*e.g.*, IMDQ, a TRL7/8 agonist), confirming that the binding affinities of nanobodies and their pharmacological activities were preserved throughout modification and release.

## 8 Radical Thiol–Ene Reactions

Thiol–ene chemistry represents an important strategy in biomedical applications thanks to its high chemoselectivity, rapid kinetics, and cytocompatibility.^142^ It was first described in 1905 ^143^ and during the last century two distinct reactions emerged: the radical addition electron-rich/poor carbon–carbon double bonds and the catalyzed ionic addition of thiolates to electron-poor carbon–carbon double bonds, the thiol-Michael reaction. Even though it was described in the last century, the application of radical thiol–ene reaction to bioconjugation chemistry is quite recent since reactive alkenes are rarely found in nature.^144^ Radical thiol–ene chemistry is classified as a click reaction as it meets all the required criteria; high yields with low amounts of easily removable byproducts; regiospecificity and stereospecificity, mild reaction conditions; compatibility with aqueous media and oxygen; orthogonality with other common reactions.^33^ Like thiol-Michael addition, radical thiol–ene reactions exploit the nucleophilicity of thiyl radicals toward carbon–carbon double bonds to form covalent thioether linkages (Scheme 52). The reaction can be thermally initiated or photoinitiated under mild conditions, but light initiation is more advantageous in bioconjugation because of its cytocompatibility.^145^ After initiation, the reaction proceeds with a propagation step, where the thiyl radical attacks the alkene, producing a new radical, which takes a hydrogen atom from another thiol molecule, regenerating a thiyl radical (Chain transfer). Termination of the reaction can occur between any radical species produced during the reaction.

**Scheme 52:**
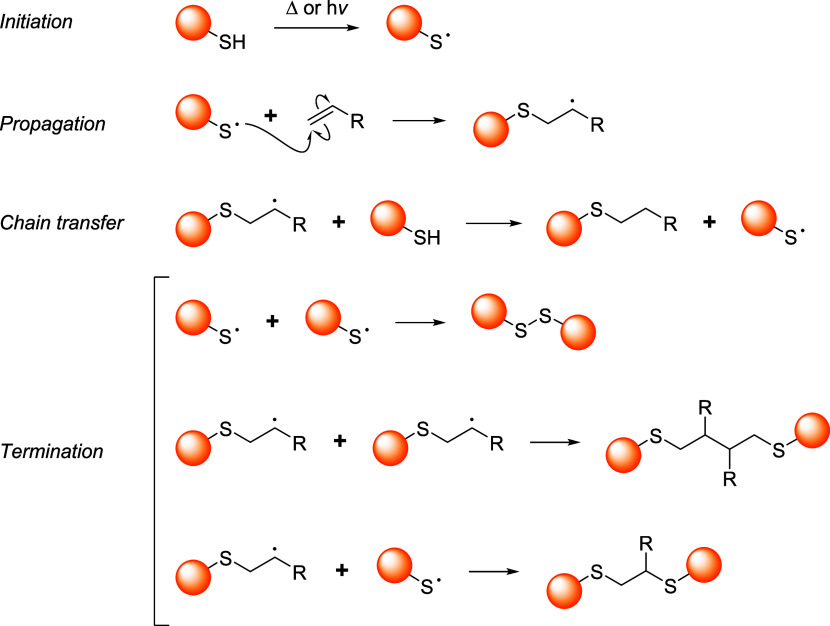
General Reaction Mechanism of Thiol Bioconjugation through Radical Thiol–Ene Reaction

Interestingly, the reaction can be performed with any thiol and is suitable for both electron-rich and electron-poor olefins. However, electron-rich alkenes (terminal olefins, norbornenes, and vinyl ethers) are the most reactive, whereas electron-poor alkenes (methacrylates, acrylonitriles, styrene, maleimides, and conjugated olefins) are the less reactive—note the reversed reactivity compared to thiol-Michael addition (Section 2).^146^ Thus, the nature of both thiol and olefin defines the reaction kinetics, which is first order overall in the monomer concentration. However, the rate-limiting step is not clearly defined: thiols with less abstractable hydrogen atoms will lead to rate-limiting chain transfers, whereas less reactive alkenes will cause rate-limiting propagations.^147^ Since propagation and chain transfer processes have large kinetic constants (from 10^5^ to 10^6^ M^–1^ s^–1^), side homopolymerization reaction, when the alkyl radical reacts with the olefin, does not occur in radical thiol–ene reactions.

The example described in this section is the work reported by Anseth, and it is a fully reversible and repeatable thiol–ene system for the patterning of proteins in hydrogels.^148^ The key chemical breakthrough lies in incorporating allyl sulfide moieties into the hydrogel backbone, enabling the regeneration of a reactive site upon the thiol–ene reaction. Thus, upon reaction with a photoinitiator, the thiol is converted into a thiyl radical (**315**), which is engaged in an addition to the allyl sulfide (**316**), producing the most stable radical (**317**). Finally, β-fragmentation releases the benzyl thiyl radical, restoring the allyl sulfide moiety (**318**) that could react in a second thiol–ene reaction (Scheme 53). Moreover, the authors already demonstrated that this strategy can be applied for the reversible patterning of peptides in hydrogels^149^ and the goal of this work is to explore a potential application with proteins. In terms of application, the authors synthesized hydrogels based on 8-arm PEG macromers bearing pendant allyl sulfide moieties, assembled via SPAAC click chemistry.

**Scheme 53:**
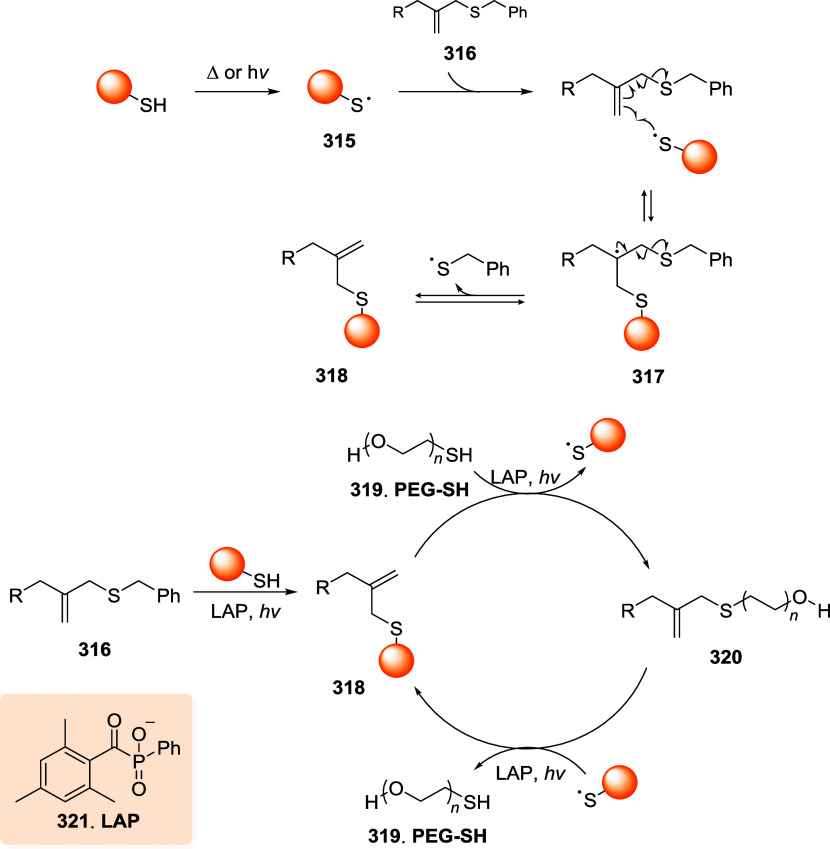
Left: Mechanism of the Bioconjugation between Thiyl Radical-Containing Protein **315** and the Allyl Sulfide **316**. Right: Strategy proposed by Anseth for the reversible tethering of proteins to hydrogels^a^

a “R” refers to the hydrogel backbone.

LAP was chosen as photoinitiator due to its rapid initiation kinetics upon irradiation at 365 nm and cytocompatibility^150^ and they identified the best conditions for transferrin tethering on allyl sulfide hydrogels (*i.e.*, laser intensity of 5 mW/cm^2^ for 180s with equimolar ratio of LAP and fluorescently labeled transferrin). Remarkably, the reaction could be tuned by changing the concentration of LAP, as shown by the linear correlation between normalized fluorescence and the photoinitiator to protein ratio, suggesting that radical propagation is indeed inhibited at low LAP concentrations. Once the optimal conditions for protein tethering were assessed, the authors attempted the release of transferrin from the hydrogel by using PEG_1K_-SH (**319**) as the scavenging thiol. The hydrogel was placed on a chrome photomask and, after irradiation, a >97% decrease in fluorescence signal was observed relative to the original pattern signal, confirming transferrin release. The use of a laser and a photomask enabled a precise spatial control over the tethering and release processes. Thus, the author managed to tether different proteins through iterative thiol–ene reactions, reproducing different 3D shapes within the hydrogel, but the most interesting application is the monitoring of cell response to changes in protein patterning. For this study, transforming growth factor-β1 (TGF-β1) was chosen as the signaling protein of interest, which activates a signaling cascade when it binds to the TGF-β1 receptor on the surface of cells, causing the transient translocation of the transcription factor Smad3 from the cytosol to the nucleus.^151^ The authors aimed to exploit Smad3 translocation as a real-time readout for TGF-β1 signaling. Thus, after both TGF-β1 and fluorescent transferrin were tethered to the hydrogel, mouse embryonic fibroblast (MEF) cells were seeded on top of the gels. MEFs were engineered to express GFP-Smad3 at endogenous levels, essential for monitoring through confocal microscopy. GFP signal was measured within the nucleus both on and off the patterned hydrogel, showing a ∼1.2 increase of fluorescence in the presence of TGF-β1, confirming the activation of the signaling pathways through the tethered protein. As a countercheck, irradiation in the presence of LAP and **319** caused the release of TGF-β1 and an ∼2.0-fold decrease of mean nuclear GFP intensity. Finally, cell viability assays showed no decrease in viability before and after irradiation, demonstrating the cytocompatibility of thiol–ene-based bioconjugation.

## 9 Conclusions and Future Perspectives

Over the past decade, thiol-selective bioconjugation chemistry has evolved from a niche methodology to a cornerstone of modern chemical biology, materials science, and biomedical engineering. The increasing variety of reversible and stimuli-responsive linkers has not only expanded the chemical toolbox but also deepened our understanding of how to precisely control the architecture and function of biomolecules under physiological conditions. Among the various strategies available, cysteine-selective approaches remain appealing due to their tunability, mild reaction conditions, and compatibility with a wide range of biomolecular systems. Importantly, this review compiled various examples of dynamic thiol bioconjugations, encompassing a wide range of linkers, bioconjugate reactions, and distinct cleavage mechanisms. The collected case studies were focused on structural diversity in linker design and the variety of stimuli that can enable deconjugation processes. However, despite the progress made in this field, several challenges still need to be addressed. One key limitation lies in achieving predictable and selective cleavage under biologically relevant conditions, especially within the complex environments of living systems. In this context, stable conjugation is especially critical in ADC development when using thiol-based chemistries, which can be inherently reversible. Several FDA-approved second-generation ADCs (*i.e.*, brentuximab vedotin—2011, enfortumab vedotin and trastuzumab deruxtecan—2019, sacituzumab govitecan, and belantamab mafodotin—2020) were mainly modified via cysteine chemistry, demonstrating the effectiveness and widespread clinical adoption of this approach. However, thiol–maleimide linkages may undergo exchange reactions or hydrolytic cleavage, leading to a partial loss or redistribution of the payload. These issues reflect broader challenges, including conjugate instability, suboptimal reaction kinetics, and nonspecific labeling, which remain primary concerns despite the clinical success of cysteine-based conjugation. This susceptibility underlines the need for more robust and long-lasting conjugation strategies to maintain the ADC integrity, pharmacokinetics, and overall therapeutic performance. These considerations are particularly relevant when discussing the translation of reversible thiol-based conjugation into clinical applications, where the dynamic behavior of such linkages must be carefully balanced with the stringent requirements of safety, reproducibility, and *in vivo* reliability. Although reversible systems hold considerable promise for controlled release, targeted delivery, and adaptive therapeutic platforms, their successful clinical implementation will depend on advances in linker design, improved mechanistic understanding, and comprehensive validation in physiologically relevant models. The inclusion of dynamic conjugation with cutting-edge technologies, such as bio-orthogonal reactions, advanced imaging, personalized and precision medicines, and diagnostics, remains underexplored, with only a few reports having been published. Future developments are expected to focus on the rational design of reversible linkers to improve biological environmental responsiveness. Advances in computational technologies and machine learning may accelerate the discovery of next-generation dynamic systems with predictable reactivity, biocompatibility, site selectivity, and tunable cleavage kinetics, thereby bridging the gap between proof-of-concept studies and clinical translation. Moreover, the choice of dynamic linkers can improve the synthesis of both new and existing polymers, enabling modular, reversible, and stimuli-responsive structures that are difficult to achieve by traditional methods. These linkers enable controlled bond formation and exchange, helping to explore the relationships among chemical structure, network dynamics, and function. This review supports the development of dynamic polymer systems in fields such as biomaterials, drug delivery, and adaptive materials, where tunable functionality is essential.
